# *Ganoderma lucidum* Polysaccharides Induce Macrophage-Like Differentiation in Human Leukemia THP-1 Cells via Caspase and p53 Activation

**DOI:** 10.1093/ecam/nep107

**Published:** 2011-01-04

**Authors:** Jia-Wei Hsu, Hsuan-Cheng Huang, Shui-Tein Chen, Chi-Huey Wong, Hsueh-Fen Juan

**Affiliations:** ^1^Institute of Molecular and Cellular Biology, Department of Life Science, Graduate Institute of Biomedical Electronics and Bioinformatics, Center for Systems Biology and Bioinformatics, Institute of Biochemical Sciences, National Taiwan University, Taiwan; ^2^Institute of Biomedical Informatics and Center for Systems and Synthetic Biology, National Yang-Ming University, Taiwan; ^3^Institute of Biological Chemistry and the Genomics Research Center, Academia Sinica, Taipei, Taiwan; ^4^Department of Chemistry and The Skaggs Institute for Chemical Biology, The Scripps Research Institute, La Jolla, CA, USA

## Abstract

Differentiation therapy by induction of tumor cells is an important method in the treatment of hematological cancers such as leukemia. Tumor cell differentiation ends cancer cells' immortality, thus stopping cell growth and proliferation. In our previous study, we found that fucose-containing polysaccharide fraction F3 extracted from *Ganoderma lucidum* can bring about cytokine secretion and cell death in human leukemia THP-1 cells. This prompted us to further investigate on how F3 induces the differentiation in human leukemia cells. We integrated time-course microarray analysis and network modeling to study the F3-induced effects on THP-1 cells. In addition, we determined the differentiation effect using Liu's staining, nitroblue tetrazolium (NBT) reduction assay, flow cytometer, western blotting and Q-PCR. We also examined the modulation and regulation by F3 during the differentiation process. Dynamic gene expression profiles showed that cell differentiation was induced in F3-treated THP-1 cells. Furthermore, F3-treated THP-1 cells exhibited enhanced macrophage differentiation, as demonstrated by changes in cell adherence, cell cycle arrest, NBT reduction and expression of differentiation markers including CD11b, CD14, CD68, matrix metalloproteinase-9 and myeloperoxidase. In addition, caspase cleavage and p53 activation were found to be significantly enhanced in F3-treated THP-1 cells. We unraveled the role of caspases and p53 in F3-induced THP-1 cells differentiation into macrophages. Our results provide a molecular explanation for the differentiation effect of F3 on human leukemia THP-1 cells and offer a prospect for a potential leukemia differentiation therapy.

## 1. Introduction

*Ganoderma lucidum* (Reishi or Ling-Zhi) is more than just an ordinary fungus; it has long been used in traditional Chinese medicinal remedies and for promotion of health and longevity, in many Asian countries. In addition, recent studies showed that *G. lucidum* is used to prevent or treat various human diseases such as allergy, hepatopathy, hypertension and cancer [1–3]. Previous evidences showed that polysaccharides extracted from *G. lucidum* have potential anti-cancer effects through the inhibition of cancer cell growth, induction of cell differentiation and suppression of angiogenesis [2, 3]. In this study, the biologically active compounds originally isolated and purified from *G. lucidum* were identified as polysaccharides, and the main fraction was designated as F3. Studies have reported that F3 is capable of inducing cytokine expression such as tumor necrosis factor-*α* (TNF-*α*) and interleukin-1 (IL-1), immunoglobulin production and cell death [4–6]. Since F3 activates a wide range of cellular responses, a systematic investigation into its molecular mechanisms would require a large-scale and genome-wide technology, for example, using microarray analysis to explore the cellular alteration and molecular disturbances induced by F3.

Previous studies showed that specific gene mutations, including lineage-specific genes and transcription factors involved in normal hematopoietic differentiation, also participate in the pathogenesis of leukemia [7, 8]. Hence, one approach for treating leukemia is differentiation therapy that outlines a treatment plan to eliminate maturation blockage and allow cell differentiation to take place [9]. The induction of terminal differentiation represents an alternative approach to the treatment of leukemia, which would generate leukemia cells with limited replicate capacity that ultimately undergo apoptosis [10]. Recently, a number of compounds were screened; for example, differentiation inducer *all-trans* retinoic acid (ATRA) is effective for leukemia treatment [11]. It was found that arsenic trioxide (As_2_O_3_) synergizes with ATRA to enhance terminal differentiation, and tyrosine kinase inhibitor gefitinib markedly activates ATRA-induced differentiation of myeloid cell lines [12–14]. Other differentiation inducers of myeloid leukemia cells have been well documented for leukemia therapy, such as 12-*O*-tetradecanoylphorbol-13-acetate (TPA) and vitamin D_3_ [15, 16].

Here, we intend to find out whether polysaccharides F3 extracted from Chinese herb *G*. *lucidum* also have similar differentiation effect on leukemia cells. THP-1, human monocytic leukemia cells, have been used as an *in vitro* macrophage differentiation model for a long time. In this study, we considered dynamic gene expression and protein activation in F3-treated THP-1 cells at different time points. Through a systems biology approach, we gathered data on possible effects induced by F3, which were verified and confirmed by various experiments. Finally, we combined the results of this study with our previous work to demonstrate plausible regulations involved in F3-induced leukemia cells, including cell death and cell differentiation.

## 2. Methods

### 2.1. Antibodies and Chemicals

The tested mouse monoclonal antibodies (mAb) include CASP9 and p21 from Upstate (Lake Placid, NY, USA), p53 from Zymed (Paisley, UK). Rabbit polyclonal antibodies include CASP6 and *β*-actin from Cell Signaling (Beverly, MA, USA), CASP8 from Santa Cruz Biotechnology (Santa Cruz, CA, USA), myeloperoxidase (MPO) from Upstate. Goat polyclonal antibody against the human matrix metalloproteinases-9 (MMP-9) was purchased from Santa Cruz Biotechnology. Secondary antibodies conjugated horseradish peroxidase raised against mouse, rabbit, and goat were purchased from Abcam (Cambridge, UK). PE-conjugated monoclonal antibodies to CD11b and CD14 and PE-conjugated isotypic control were purchased from BD Pharmingen (San Diego, CA, USA). The pan-caspase inhibitor z-Val-Ala-Asp-fluoromethyl ketone (Z-VAD-FMK; R&D Systems, Abington, UK) and the p53 inhibitor pifithrin-*α* (PET-*α*; Sigma-Aldrich, St Louis, MO, USA) were dissolved in dimethylsulfoxide (DMSO).

### 2.2. THP-1 Cell Culture, Purification of Reishi Extract, Isolation of the F3 Fraction of Reishi Polysaccharides, and Microarray Gene Expression Profile Analysis

F3 was isolated from the water soluble residue of Reishi polysaccharide and purified by gel filtration chromatography using a Sephacryl S-500 column (95 × 2.6 cm) with 0.1 N Tris buffer (pH 7.0) as the eluent. The carbohydrate composition analyses of F3 indicated that glucose (58.1%), mannose (15.1%) and Galactose (13.5%) exist as the major components together with smaller amounts of other sugars, including fucose, N-acetylglucosamine, xylose and rhamnose. Detailed experiments were performed according to our previous report [6].

### 2.3. Significant Functional Networks and Biological Pathway Annotations

The differentially expressed genes were annotated by Ingenuity Pathway Analysis (IPA; Ingenuity Systems, Redwood City, CA, USA). In brief, the identified genes were mapped onto available functional networks and specific biological pathways, and then ranked by score. The score is based on a *P*-value calculation; for example, if the score is 3, then the corresponding *P*-value is 10^−3^, meaning there is a 1/1000 chance that the focus genes are in a network due to random chance. The significance of the association between the data set and the canonical pathway was measured in two ways, ratio and *P*-value. Ratio is displayed as the number of genes from the data set that map to the pathway divided by the total number of genes that map to the canonical pathway. Fischer's exact test was used to calculate a *P*-value determining the probability that the association between the genes in the dataset and the canonical pathway is explained by chance alone. The statistical analysis of biofunctions and disturbed pathways were sorted on the order of matching significance by using IPA web tool.

### 2.4. Morphologic Changes and Quantification of Adherent Cells

Under normal culture conditions THP-1 cells remain in suspension; however, after treatment with F3 a subpopulation of cells becomes adherent within 24 or 48 h. Morphologic changes of the adherent cells were assessed by Liu's staining under phase contrast microscopy. In addition, the supernatants were then collected in 15-ml tubes and the plates were washed three times with PBS and combined with the supernatant. The unattached cells were centrifuged, resuspended in 200 *μ*l, and counted in order to determine the adherence with a hemacytometer.

### 2.5. NBT Reduction Assay

Nitroblue tetrazolium (NBT; Sigma) can be used to determine the amounts of intracellular O^2−^ produced by phagocytic cells. Thus, this assay is sensitive enough to measure, quantitatively, even the small amounts of O^2−^ produced in macrophages. For NBT assay, cells were seeded in 24-well plates at a density of 2 × 10^5^ cells/ml and incubated with or without F3. Different numbers of cells attached to a 24-well culture plate were incubated with 200 *μ*l RPMI-1640 medium containing 1 mg/ml NBT and 4 *μ*g/ml phorbol 12-myristate 13-acetate (PMA; Sigma) and then incubated at 37°C for 1 h. After incubation, cells were washed twice with warm PBS, and then pelleted. The pellets were resolved in 100 *μ*l DMSO. Absorbance at 570 nm was detected with spectrometer.

### 2.6. Cell Cycle Analysis

A total of 1 × 10^6^ cells were collected into ice-cold PBS, and fixed with ice-cold 70% ethanol in PBS for at least 24 h at 4°C. Cells were stained using 10 *μ*g/ml propidium iodide (PI) solution (Santa Cruz Biotechnology) and RNaseA (Santa Cruz Biotechnology) in PBS in the dark for at least 30 min at room temperature. The percentages of cells in the different phases of the cell cycle was measured with a FACS flow cytometer (Becton Dickinson, San Jose, CA, USA) and analyzed by using CellQuest software (Becton Dickinson).

### 2.7. Western Blot

Western blot was performed as previously described [6]. Briefly, F3-treated and untreated THP-1 cells were washed with PBS twice. Cell pellets (1 × 10^7^ cells) were solubilized in lysis buffer containing 7 M urea, 4% CHAPS, 2M thiourea and 0.002% bromophenol blue. Lysates were centrifuged at 13 200 g for 30 min. Proteins were loaded into 10% SDS-PAGE and transferred onto polyvinylidene difluoride membranes (Millipore, Bedford, MA, USA) at 150 V for 1.5 h. After blocking in 5% non-fat milk in PBST containing 0.05% Tween 20 (Sigma) at room temperature overnight with gentle rocking, membranes were probed with antibodies. Membranes were incubated with corresponding primary antibody overnight at 4°C and then incubated with secondary antibodies. After incubation with secondary antibodies, immunoblots were visualized with the ECL detection kit (Amersham Biosciences) and exposed to X-ray film. *β*-actin was used as an internal loading control.

### 2.8. Primer Design

Primers were designed based on the published sequences in NCBI. We used Beacon Designer to design the primers. Primers were designed to meet the following requirements: 75–150 bp in length, 50–60% in CG content, less than 5 degrees Tm difference between forward and reverse primers, limited GC repeats, amplicons between 80–120 bp, and limited dimer and hairpin formation. The genes and primers used for RT-PCR and real-time PCR are performed using the following primer sets: CD11b (forward: 5′-CAA GGA AGC CGG AGA GGT CAG A-3′; reverse: 5′-CGG AGT CCA GAG CCA GGT CAT AAG-3′); CD14 (forward: 5′-CAC AGC CTA GAC CTC AGC CAC AAC-3′; reverse: 5′-CCA GCC CAG CGA ACG ACA G-3′); CD68 (forward: 5′-CAC CTC CAA GCC CAG ATT CAG AT-3′; reverse: 5′-CCT TGG TTT TGT TGG GGT TCA GTA-3′); MPO (forward: 5′-CTT CGT CAC TGG CGT CAA CTG-3′; reverse: 5′-AGG AGC GGA AGA ACG GGA TG-3′); MMP-9 (forward: 5′-ACC AAG TGG GCT ACG TGA CCT ATG-3′; reverse: 5′-GTA TCC GGC AAA CTG GCT CCT T-3′).

### 2.9. Quantitative Real-Time PCR

Detection of gene expression by qualitative real-time PCR (Q-PCR) was determined essentially. Briefly, total RNA were isolated from cell lines and first-strand cDNA synthesis was generated. Extracted first-strand cDNAs were analyzed using a BioRad iCycler iQ Real-Time Detection System with SYBR Green dye (Molecular Probes, Eugene, OR, USA). SYBR Green yields a strong fluorescent signal on binding double-stranded DNA enabling the quantification of gene expression by measurement of the intensity of the fluorescent light. mRNA expression of these genes were normalized to RNA content for each sample by using *β*-actin gene products as internal controls. A standard curve was also run in each PCR reaction. Fold changes were calculated using the ΔΔCt method with *β*-actin as the reference gene amplified from the samples. All reactions were run in triplicate. The reaction products were separated on 2% agarose gel and stained with ethidium bromide for product length check.

### 2.10. Flow Cytometry

The surface expression of macrophage differentiation marker CD11b and CD14, or binding of PE-conjugated mouse IgG2a was determined by flow cytometric analysis as previously described [17]. The harvested cells were washed with PBS followed by incubation with the PE-conjugated anti-human CD11b and CD14 for 1 h. For each marker, a total of 10 000 events were collected from the gated area detecting viable cells. The results were collected as either percentage of positive cells or mean fluorescence intensity (MFI) values subtracted by the MFI of isotype control antibody-stained cells. The flow cytometric analysis was performed on the FACS system, and the data were analyzed using CellQuest software. The isotype control PE-conjugated mouse IgG2a mAb-stained cells were used as the background control in all experiments.

### 2.11. Statistical Analysis

Results are shown as mean ± SD from more than three independent experiments. The two-tailed Student's *t*-test was used to compare different groups. Values of *P* < .05 were considered significant.

## 3. Results

### 3.1. Significant Functional Networks and Biological Pathways Involved in F3-Induced THP-1 Cells

**Figure 1:**
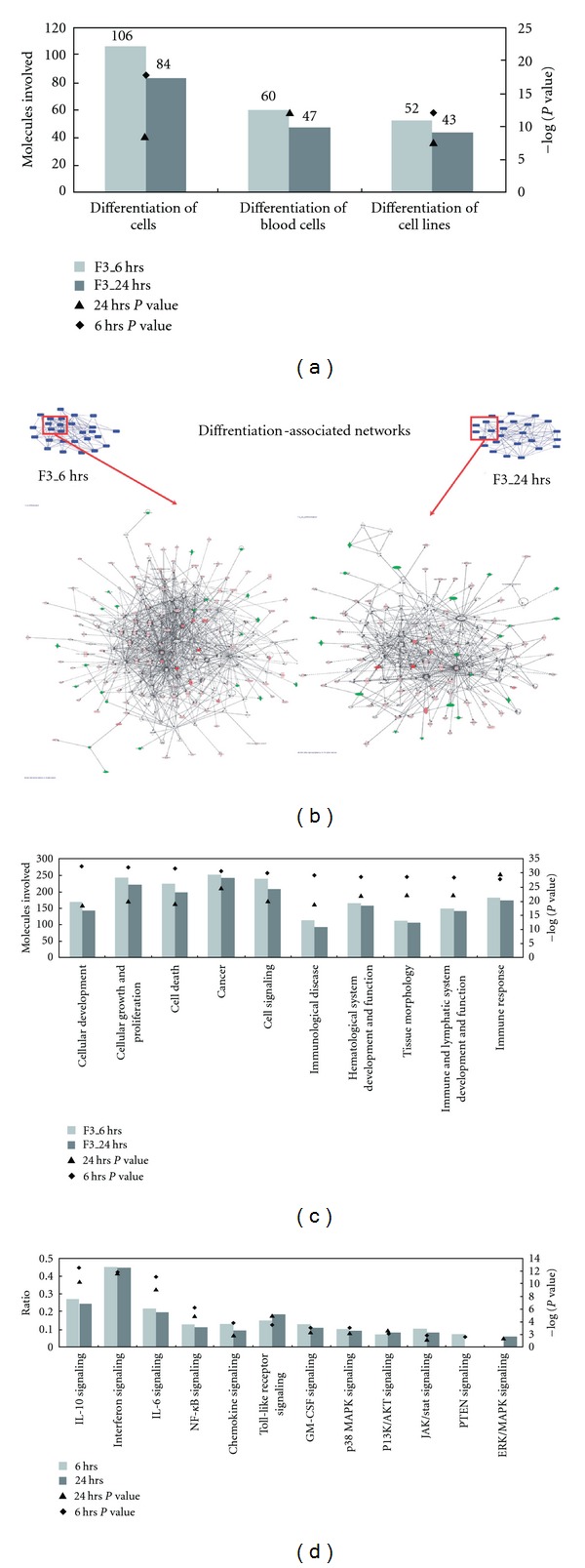
F3-induced gene expression profiles in THP-1 cells associated with cell differentiation were analyzed by the IPA tool. (a) Molecules involved in the process of cell differentiation are presented. (b) Interaction network analysis as a framework for the interpretation of cell differentiation was performed. This shows the major functional networks (c) and biological pathways (d) that were found to be significantly associated with cell differentiation (*P* < .05).

In a prior study, *G. lucidum* polysaccharides have been used as active compounds for immuno-modulation and to display anti-tumor activity; little attention has been paid to its effect on the process of cancer cell differentiation [18]. Here, we used high-throughput microarray to screen and investigate the dynamic patterns of gene expression and analyzed possible biological functions and physiological role of F3-treated leukemia cells through a bioinformatics approach. All the differentially expressed genes were identified and are showed in Supplementary Tables S1 and S2. We have submitted the array data to the GEO database and the series record is GSE16014. In order to study the effects induced by F3, we identified the gene expression patterns by oligonucleotide microarray analysis. Genes with more than 2-fold change in expression levels between control and F3-treated THP-1 cells were sorted into categories according to their annotation in IPA. The IPA knowledge-based database provides annotations regarding biological functions and signaling. There were 115 and 96 significant genes associated with cell differentiation after F3 treatment for 6 and 24 h, respectively (Figure 1(a)). Furthermore, these significant genes were selected for mapping significant functional protein-protein interaction networks (Figure 1(b)). The top five significant protein-protein interaction networks affected by F3 were chosen for further analysis (Supplementary Table S3). These networks describe the functional relations between gene products based on known interactions. The observed significant networks were associated with cell death and cellular development. On the basis of these selected networks, a total of 10 biological functions were found to be highly significant, the main one being cellular development, which depicts functions associated with the development and differentiation of cells (Figure 1(c)). Moreover, we used the IPA tool to connect these networks with known biological pathways. Significant biological pathways highly associated with cell differentiation are shown in Figure 1(d). For example, IL-6 signaling regulates cell differentiation as well as cell growth arrest through the activation of transcription factor STAT3 [19]. NF-*κ*B signaling plays a critical role in the development and function of all hematopoietic cell types [20]. Induction of GM-CSF signaling effectively differentiates monocytes to a more macrophage-like phenotype [21]. Interestingly, the disturbed pathways in F3-treated THP-1 cells closely resembled the canonical pathways found out in differentiated macrophages derived from GM-CSF-treated blood monocytes [22]. Therefore, the dynamic gene expression profiling provides a feasible approach to discover cell differentiation pathways which involved in F3-induced leukemia cells. 

### 3.2. Leukemia Cells Differentiation into Macrophage-Like Cells Induced by F3

**Figure 2:**
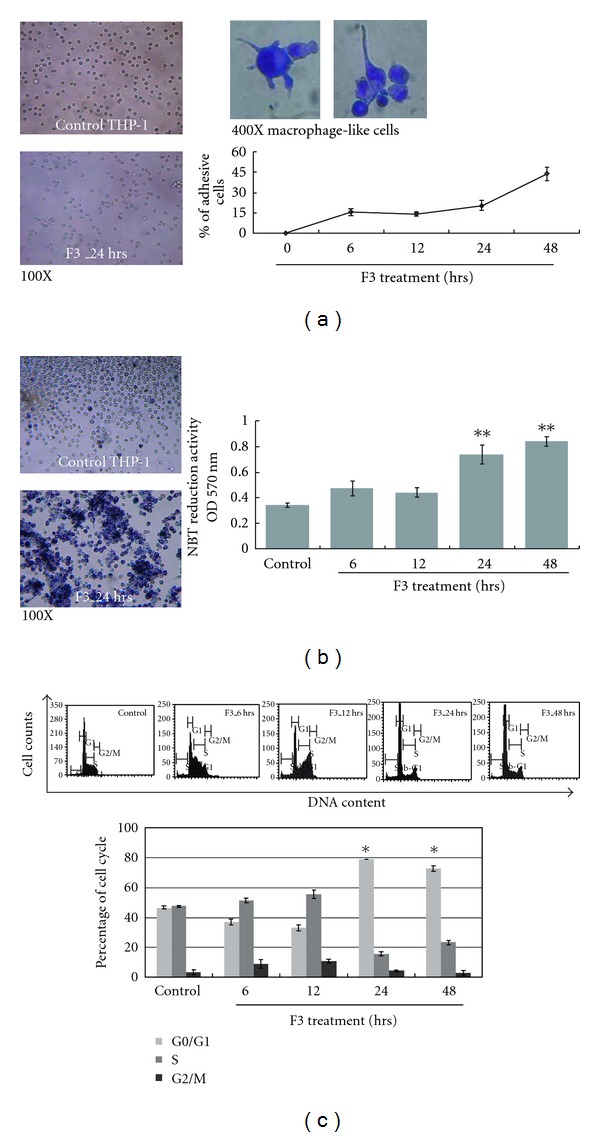
Potential of F3-induced leukemia cells to differentiate into macrophages. (a) Effect of F3 on cell adherence is shown under phase-contrast microscope. THP-1 cells were incubated with/without 30 *μ*g/ml of F3, and macrophage-like cells were shown by Liu's stain. Quantification of cell adherence is shown in the lower panel where the percentage of adhesive THP-1 cells is presented. The data were shown as mean ± SD from three independent experiments. (b) F3 treatment was able to increase NBT reduction significantly. NBT reduction assay showed insoluble and visible blue formazan precipitates in F3-treated THP-1 cells. The blue formazan precipitates in F3-treated THP-1 cells were measured by absorbance at 570 nm. The data were shown as mean ± SD from three independent experiments. Individual Student's *t*-test was performed between F3-treated leukemia cells and untreated control (**P < .001). (c) Summary of cell cycle analysis is shown in this panel where the percentage of leukemia cells in each cell-cycle phase is shown. The data were shown as mean ± SD from three independent experiments.

The potential role of *G. lucidum* polysaccharides F3 in cell differentiation was also studied. THP-1 cells were treated with 30 *μ*g/ml of F3 for different time courses. Cell adhesion, a hallmark of macrophage differentiation, was examined under a phase-contrast microscope. Adhesive and non-adhesive cells were counted by trypan blue exclusion. The treatment of F3 greatly changed the cell morphology and increased leukemia cell adherence (Figure 2(a)). In the presence of F3, about 45% of THP-1 cells were observed to be attached to the culture wells, and macrophage-like cell patterns were observed by Liu's staining (Figure 2(a)). These data provide a perspective that *G. lucidum* polysaccharides F3 might have the potential to induce differentiation. To further examine the effects of F3 on leukemia cell differentiation, we applied the following assays. First, F3-induced differentiation of leukemia cells was determined by NBT reduction assay, a functional marker to evaluate the ability of superoxide production during macrophage differentiation [23]. Our data show that F3 greatly enhances superoxide production after 24 h of treatment, as demonstrated by the presence of intracellular purple formazan deposit (Figure 2(b)). Furthermore, time-course study observed significant increase in the levels of NBT reduction, indicating that F3 was capable of enhancing NBT reduction (Figure 2(b)). Previous studies revealed that irreversible arrest in the cell cycle is a hallmark of terminally differentiated myeloid cells [24]. Therefore, we analyzed the effect of F3 on cell cycle progression in leukemia cells. As shown in Figure 2(c) with F3 treatment, THP-1 cell population at G0/G1 phase increased from 46 to 80%. These data demonstrate that F3 is able to induce cell cycle arrest in leukemia cells. These results showed that F3 can induce leukemia cells differentiation to macrophage-like cells.

### 3.3. F3-Induced Expression of Specific Macrophage Differentiation Markers

**Figure 3:**
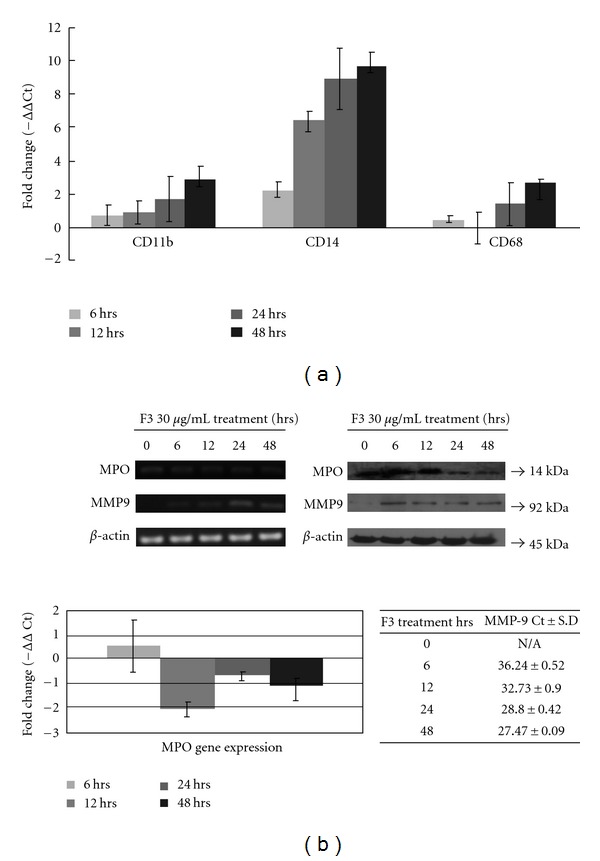
Expression of macrophage differentiation markers in F3-induced THP-1 cells were examined by Q-PCR and western blotting. (a) Gene expressions of classical markers of macrophage differentiation, CD11b, CD14 and CD68, were determined by Q-PCR. (b) Other macrophage differentiation markers, MPO and MMP9, were also determined by Q-PCR and western blotting. mRNA expression of these genes was normalized to RNA content for each sample by using *β*-actin as internal controls; *β*-actin was also used as internal loading controls in western blotting.

**Figure 4:**
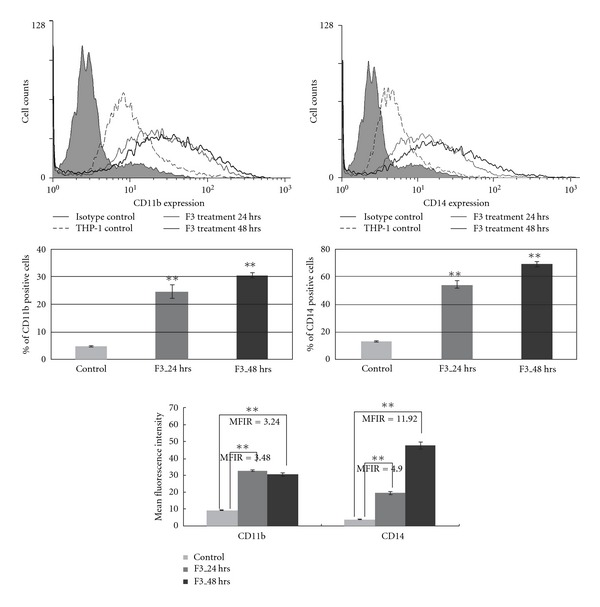
Flow cytometry detection of CD11b and CD14 expression in leukemia cells upon treatment with F3. The results were collected as either percentage of positive cells or MFI values subtracted by the MFI of isotype control antibody-stained cells. The results are the representation of three independent experiments showing similar results. Values were shown as mean ± SD and individual Student's *t*-test was performed between F3-treated THP-1 cells and untreated control (**P < .001). MFIR: mean fluorescence intensity ratio.

To make certain that F3 alone was sufficient for macrophage differentiation to occur, we analyzed specific cell markers and enzyme activity related to macrophage differentiation. As shown in Figure 3(a), the gene expression levels of CD11b, CD14 and CD68 were markedly elevated in F3-induced THP-1 cells. The enzyme MPO is synthesized only in myeloid and monocytic cells; therefore, downregulation of MPO activity is a characteristic feature in macrophage differentiation, making it an important marker of the myeloid lineage [25]. Here, F3 treatment reduced both overall MPO mRNA level and MPO protein activity in THP-1 cells (Figure 3(b)). Previous reports indicated that matrix metalloproteinase-9 (MMP-9) is produced during the macrophage differentiation process [26]. Initially, both MMP-9 mRNA expression and MMP-9 protein activity were not detected in THP-1 control; however, MMP-9 mRNA and protein production were identified in F3-treated THP-1 cells (Figure 3(b)). Furthermore, we examined the expression levels of the specific macrophage markers by flow cytometric analysis. Our data showed that treatment of THP-1 cells with F3 for 24 and 48 h could induce a significant increase in the expression of CD11b and CD14 (Figure 4). Taken together, these data indicate that leukemia cells would differentiate into macrophages after F3 treatment. These results were also consistent with the gene expression profiles explored using the IPA tool.

### 3.4. Regulation of F3-Induced Macrophage Differentiation via Caspase Cleavage and p53 Activation

**Figure 5:**
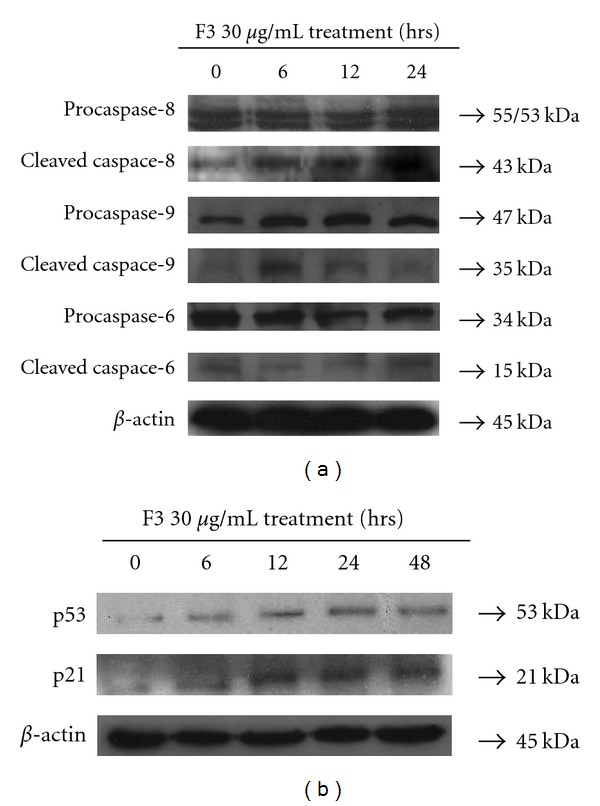
Cells were treated with F3, and then cell lysates were obtained for western analyses to observe the expressions of macrophage differentiation-associated protein. Detection of (a) caspase cleavage and (b) p53/p21 was determined by western blotting.

Our previous studies demonstrated that F3 is able to induce apoptosis via the death receptor pathway. Caspase 3 and 7 were cleaved into their active form in F3-treated THP-1 cells, while several lines of evidence indicated that active caspase cleavage also contributes to the differentiation of monocytes into macrophages [6, 27, 28]. However, caspase activation does not involve the differentiation into dendritic cells, indicating that the differentiation is lineage-specific [27]. To investigate whether caspases were activated during the differentiation process in this study, we studied the time-dependent appearance of caspase cleavage fragments by western blotting. Combining our previous data, we also observed the presence of caspase-8 and caspase-9 in their active forms in F3-treated THP-1 cells, indicating that caspases cleavage might be involved in this differentiation process (Figure 5(a)). One thing worth noting was that the cleavage of caspase 6 was not noticeably seen, possibly implying its minor contribution to macrophage differentiation. Moreover, several *in vitro* and *in vivo* assays have shown that p53 expression in undifferentiated cells could result in cells moving into a differentiated state [29, 30]. Generally, p21 can be transcriptionally activated by wild-type p53, and the induction of p21 expression has been shown in several systems to lead to *in vitro* differentiation [31]. In this study, we examined the expression of p53 and its downstream molecule p21 by western blotting. The result showed that treatment of THP-1 cells with F3 could elevate p53 and p21 expressions and thus lead to macrophage differentiation (Figure 5(b)). Here, we identified the activation of p53 and several caspases cleavage in THP-1 cells that contribute to the differentiation into macrophages in response to F3.

**Figure 6:**
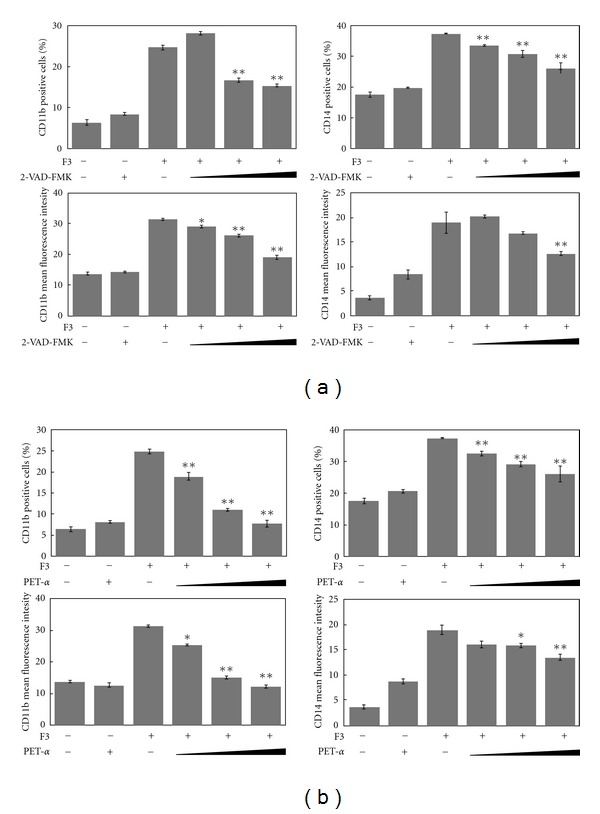
The effects of caspase inhibitor (Z-VAD-FMK) or p53 inhibitor (PET-*α*, and F3 alone or in combination with inhibitors, on the differentiation of THP-1 cells into macrophages were observed by flow cytometry analysis. (a) Caspase inhibitors (6, 20, 50 *μ*M) or (b) p53 inhibitors (5, 50, 100 *μ*M) were added to either the control or F3-treated cultures after 24 h of incubation. The percentage of CD11b and CD14 positive cells and MFI of CD11b and CD14 positive cells were shown as the mean ± SD, and individual Student's *t*-test was performed between F3-treated THP-1 cells and caspase inhibitor combined with F3 co-cultured groups (*P < .01, **P < .001) in comparison to F3-treated cells.

To further elucidate the effects of caspase cleavage and p53 activation on macrophage differentiation in F3-treated THP-1 cells, caspase and p53 inhibitors were applied.  Figure 6(a) shows that the presence of general caspase inhibitor Z-VAD-FMK alone did not induce the expression of differentiation markers while it reduced the appearance of F3-induced CD14 positive cells and blocked CD11b expression levels in a dose-dependent manner. Our previous study showed that apoptotic events were initiated via the death receptor signaling after F3 treatment in THP-1 cells. The function of F3 is rather complex as it may involve various biological effects. Our published work suggested that F3 might mimic/induce death receptor ligands, such as TNF-*α* and tumor necrosis factor-related apoptosis inducing ligand (TRAIL), to initiate signaling in THP-1 cells, and a recent study clarified that caspase activation mediates TRAIL cytotoxicity and monocytic maturation of HL-60 cells [32]. According to our study, F3 could in fact enhance macrophage differentiation of leukemia cells THP-1 and mimic/induce TRAIL to trigger apoptosis through the caspase cascade. The effects of PET-*α*, a chemical inhibitor of p53 transcriptional activity, on macrophage differentiation were similar to the presence of caspase inhibitor (Figure 6(b)). PET-*α* significantly reduces the percentage of THP-1 cells expressing cell surface markers, that is, CD11b and CD14, in response to F3 in a dose-dependent manner; PET-*α* also decreases the expression levels of CD11b and CD14 in F3-treated THP-1 cells. These results showed that caspase cleavage and p53 activation can contribute to the macrophage differentiation in F3-treated THP-1 cells.

## 4. Discussion

For understanding more about molecular regulation and gene expression profiles, we used the high-throughput screening method, microarray chip and bioinformatics tools, to investigate the dynamic gene expression patterns and related molecular mechanisms. In our previous study, functional networks associated with cell death and the pathway of apoptosis induction through DR3 and DR4/5 death receptors were found to be significant in F3-treated THP-1 cells for 6 and 24 h [6]. We further observed irregular and rough edges of cell membrane under the phase-contrast microscope, incremental to the chromatin condensed cells, and signs of phosphatidylserine release. These results indicated that cell shrinkage happened and somehow apoptosis followed. Recent studies also showed *G*. *lucidum* to induce apoptosis in many cancer cells such as lung cancer cells, colon carcinoma cells, prostate cancer cells and breast cancer cells. In our Q-PCR gene expression results, CASP8 showed a significant up-regulated expression. Caspase 8, 3, 7 were also cleaved into their active forms after F3 treatment. Our previous results suggested that F3 might mimic/induce death receptor ligands such as TNF-*α* and TRAIL to initiate signaling via death receptor oligomerization, recruitment of specialized adaptor proteins and the activation of caspase cascade, followed by cell shrinkage and apoptosis [6].

In addition to cell death effect, cell differentiation was also important in F3-treated THP-1 cells (Figure 1). Such an effect of F3 on cell differentiation was confirmed by change of cell morphology from monocytic phenotypes to macrophage-like cells and the elevated cell adherence percentage as well (Figure 2(a)). Another two assays, NBT reduction and cell cycle analysis, suggest the effect of differentiation to macrophage-like cells. F3 treatment for 24 and 48 h increased NBT reduction activity markedly (Figure 2(b)) and together with arrest in the G0/G1 phase of the cell cycle (Figure 2(c)). Crucially, up-regulation of CD11b, CD14, CD68 and MMP-9, and downregulation of MPO, strongly suggested the effect of macrophage differentiation in F3-treated THP-1 cells (Figures 3 and 4).

Previous reports showed that protein cleaved by caspase contributes to macrophage differentiation process [27, 28]. However, caspase activation does not involve in differentiation into dendritic cells, indicating that specific lineage differentiation is determined by these enzymes [27]. Here, caspase cleavage was identified during macrophage differentiation in F3-treated THP-1 cells (Figure 5(a)) and general effector caspase inhibitor blocked the expression of macrophage differentiation markers (Figure 6(a)). Interestingly, caspase activation is required for the differentiation of monocytes into macrophages and occurs in the absence of apoptosis feature, while our previous studies showed apoptotic events were initiated via death receptor signaling after F3 treatment in THP-1 cells. Therefore, F3-induced cell differentiation associated with cell death gives unlike account from TPA induction of macrophage differentiation without apoptotic features [27]. Our published work has suggested that F3 might mimic/induce death receptor ligands such as TNF-*α* and TRAIL to initiate signaling in THP-1 cells, and a recent study has clarified that caspase activation mediates TRAIL cytotoxicity and monocytic maturation of HL-60 cells [32]. We suggest that F3 could in fact mimic/induce TRAIL to trigger apoptosis and macrophage differentiation of leukemia cells THP-1 through caspase cascade.

It was indicated in several experimental models that wild-type p53 and p21 facilitate cellular differentiation of various cell types, including the regulation of monocytic maturation as well [29, 30]. Treatment of F3 in THP-1 cells could gradually enhance p53 and p21 expressions during macrophage differentiation (Figure 5(b)), and inhibition of p53 activity could reduce the expression of macrophage differentiation markers (Figure 6). Additionally, p53 and p21 activities might partially contribute to the accumulation of cell cycle progression in the G0/G1 phase. The previous study indicated that cell cycle arrest and cell differentiation are determined via the p53-dependent pathway [30].

**Figure 7:**
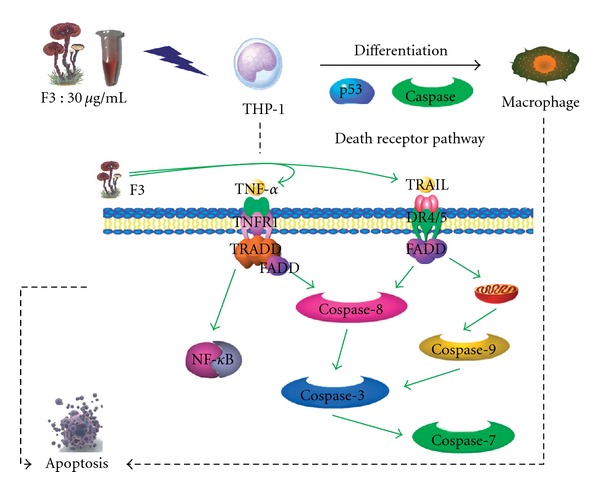
A proposed model to characterize the intricate machinery in F3-induced THP-1 cells. F3 might mimic/induce death receptor ligands such as TNF-*α* and TRAIL to trigger apoptosis and macrophage differentiation of leukemia cells THP-1 through caspase cascade and p53-dependent pathway.

Studies have reported that F3 is capable of inducing cytokine expression such as TNF-*α* and IL-1 and immunoglobulin production [4, 5]. In our study, we have examined two dominant effects caused by F3, cell death and cell differentiation. We proposed a model to characterize the intricate machinery in F3-induced THP-1 cells, as illustrated in Figure 7. Our study showed that F3 might exhibit anti-tumor activity based upon the induction of differentiation and apoptosis. The anti-tumor activity of F3 may be similar to the TPA-differentiated HL-60 cells followed by subsequent cell death [33]. Apoptosis might be observed as a consequence of terminal differentiation.

In summary, we integrated dynamic gene expression patterns and protein activation profile to infer possible regulations involved in F3-induced macrophage differentiation. We suggest that F3 could induce leukemia cells differentiation to macrophages, as demonstrated by enhancing the percentage of cell adherence, superoxide production, cell cycle arrest and expression of differentiation markers. The results in this study revealed the molecular regulatory mechanism of F3 involved in human leukemia cell differentiation and F3 can be a potent natural compound for leukemia differentiation therapy.

## Supplementary Data

Supplementary data are available at *eCAM* online.

## Funding

National Science Council of Taiwan (96-2627-M-002-010- & 96-2311-B-002-018-), NTU Frontier and Innovative Research Projects (NTUPFIR-96R0107) and The Thematic Research Program, Academia Sinica, Taiwan.

## Supplementary Material

Supplementary Table 1: The differentially expressed genes in F3-treated THP-1 cells after 6 hours.
Supplementary Table 2: The differentially expressed genes in F3-treated THP-1 cells after 24 hours.
Supplementary Table 3: Significantly genetic networks affected by F3.
