# Supplementary material for: Ganoderma lucidum Polysaccharides Induce Macrophage-Like Differentiation in Human Leukemia THP-1 Cells via Caspase and p53 Activation
Source: Evid Based Complement Alternat Med. 2011 Jan 4;2011:358717. doi: 10.1093/ecam/nep107 (PMC3135330; doi:10.1093/ecam/nep107)
Supplement: Supplementary file 1 — Supplementary Table 1: The differentially expressed genes in F3-treated THP-1 cells after 6 hours. Supplementary Table 2: The differentially expressed genes in F3-treated THP-1 cells after 24 hours. Supplementary Table 3: Significantly genetic networks affected by F3. [file 358717.f1.pdf]

supplementary Table 1. The differentially expressed genes in F3-treated THP-1 cells after 6 hours.

**Gene Symbol**	**Chip ID**	**Fold Change**	**Genbank**	**UniGene**	**Description**
CCL20	205476_at	421.7	NM_004591	Hs.75498	chemokine (C-C motif) ligand 20
CCL4	204103_at	351.6	NM_002984	Hs.75703	chemokine (C-C motif) ligand 4
BCL2A1	205681_at	228.6	NM_004049	Hs.227817	BCL2-related protein A1
ISG20	204698_at	202.1	NM_002201	Hs.459265	interferon stimulated exonuclease gene 20kDa
CCL8	214038_at	139.1	AI984980	Hs.271387	chemokine (C-C motif) ligand 8
CXCL1	204470_at	115.1	NM_001511	Hs.789	chemokine (C-X-C motif) ligand 1 (melanoma growth stimulating activity, alpha)
IL7R	205798_at	111.6	NM_002185	Hs.362807	interleukin 7 receptor ; interleukin 7 receptor
BCL3	204908_s_at	104.2	NM_005178	Hs.31210	B-cell CLL/lymphoma 3
GBP1	202269_x_at	99.99	BC002666	Hs.62661	guanylate binding protein 1, interferon-inducible, 67kDa
SLAMF7	219159_s_at	98.3	NM_021181	Hs.517265	SLAM family member 7
CXCL10	204533_at	93.05	NM_001565	Hs.413924	chemokine (C-X-C motif) ligand 10
IL8	211506_s_at	91.31	AF043337	Hs.624	interleukin 8
IL8	202859_x_at	85.26	NM_000584	Hs.624	interleukin 8
THBS1	201110_s_at	84.5	NM_003246	Hs.164226	thrombospondin 1
EBI3	219424_at	82.87	NM_005755	Hs.501452	Epstein-Barr virus induced gene 3
SAT	213988_s_at	76.47	BE971383	Hs.28491	spermidine/spermine N1-acetyltransferase
USP18	219211_at	76.22	NM_017414	Hs.38260	ubiquitin specific peptidase 18
LAMP3	205569_at	75.89	NM_014398	Hs.518448	lysosomal-associated membrane protein 3
CCL2	216598_s_at	71.33	S69738	Hs.303649	chemokine (C-C motif) ligand 2
IL1B	205067_at	65.51	NM_000576	Hs.126256	interleukin 1, beta
IL1B	39402_at	64.08	M15330	Hs.126256	interleukin 1, beta
CXCL13	205242_at	61.28	NM_006419	Hs.100431	chemokine (C-X-C motif) ligand 13 (B-cell chemoattractant)
CXCL11	211122_s_at	59.72	AF002985	Hs.518814	chemokine (C-X-C motif) ligand 11
TNFAIP6	206025_s_at	57.53	AW188198	Hs.437322	tumor necrosis factor, alpha-induced protein 6
IGFBP5	211959_at	56.92	AW007532	Hs.369982	insulin-like growth factor binding protein 5
CXCL3	207850_at	55.61	NM_002090	Hs.89690	chemokine (C-X-C motif) ligand 3
TNFAIP6	206026_s_at	51.77	NM_007115	Hs.437322	tumor necrosis factor, alpha-induced protein 6
THBS1	201109_s_at	46.24	AV726673	Hs.164226	thrombospondin 1
CCL3	205114_s_at	44.61	NM_002983	Hs.514107	chemokine (C-C motif) ligand 3 ; chemokine (C-C motif) ligand 3-like 1
GPR132	221140_s_at	40.77	NM_013345	Hs.532504	G protein-coupled receptor 132
DUSP6	208893_s_at	40.29	BC005047	Hs.298654	dual specificity phosphatase 6
CALCRL	210815_s_at	40.19	U17473	Hs.470882	calcitonin receptor-like
IGFBP3	210095_s_at	39.14	M31159	Hs.450230	insulin-like growth factor binding protein 3
SOD2	215223_s_at	38.22	W46388	Hs.487046	superoxide dismutase 2, mitochondrial
IL23A	220054_at	37.77	NM_016584	Hs.98309	interleukin 23, alpha subunit p19
PTGS2	204748_at	37.69	NM_000963	Hs.196384	prostaglandin-endoperoxide synthase 2 (prostaglandin G/H synthase and cyclooxygenase)

IL12B	207901_at	37.1	NM_002187	Hs.674	interleukin 12B (natural killer cell stimulatory factor 2, cytotoxic lymphocyte maturation factor 2, p40)
FCAR	211307_s_at	34.64	U43677	Hs.193122	Fc fragment of IgA, receptor for
IFIT1	203153_at	34.37	NM_001548	Hs.20315	interferon-induced protein with tetratricopeptide repeats 1
HESX1	211267_at	31.14	U82811	Hs.171980	homeo box (expressed in ES cells) 1
LRRC50	222068_s_at	29.96	AW663632	Hs.310164	leucine rich repeat containing 50
IFIT5	203596_s_at	29.41	NM_012420	Hs.252839	interferon-induced protein with tetratricopeptide repeats 5
IGFBP3	212143_s_at	28.33	BF340228	Hs.450230	insulin-like growth factor binding protein 3
CXCL2	209774_x_at	28.14	M57731	Hs.75765	chemokine (C-X-C motif) ligand 2
TNF	207113_s_at	27.69	NM_000594	Hs.241570	tumor necrosis factor (TNF superfamily, member 2)
IL15RA	207375_s_at	27.67	NM_002189	Hs.524117	interleukin 15 receptor, alpha
CXCL11	210163_at	25.9	AF030514	Hs.518814	chemokine (C-X-C motif) ligand 11
KITLG	211124_s_at	25.64	AF119835	Hs.1048	KIT ligand
ISG20	33304_at	25.61	U88964	Hs.459265	interferon stimulated exonuclease gene 20kDa
MAFB	218559_s_at	25.46	NM_005461	Hs.169487	v-maf musculoaponeurotic fibrosarcoma oncogene homolog B (avian)
CCL7	208075_s_at	24.26	NM_006273	Hs.251526	chemokine (C-C motif) ligand 7 ; chemokine (C-C motif) ligand 7
SLAMF8	219386_s_at	23.8	NM_020125	Hs.438683	SLAM family member 8
SLC2A6	220091_at	23.03	NM_017585	Hs.244378	solute carrier family 2 (facilitated glucose transporter), member 6
GCH1	204224_s_at	22.89	NM_000161	Hs.86724	GTP cyclohydrolase 1 (dopa-responsive dystonia)
FEZ1	203562_at	22.65	NM_005103	Hs.224008	fasciculation and elongation protein zeta 1 (zygin I)
NFKB2	209636_at	22.44	BC002844	Hs.73090	nuclear factor of kappa light polypeptide gene enhancer in B-cells 2 (p49/p100)
CXCL6	206336_at	22.12	NM_002993	Hs.164021	chemokine (C-X-C motif) ligand 6 (granulocyte chemotactic protein 2)
ICAM1	202638_s_at	21.72	NM_000201	Hs.515126	intercellular adhesion molecule 1 (CD54), human rhinovirus receptor
MSC	209928_s_at	21.44	AF060154	Hs.442619	musculin (activated B-cell factor-1)
APOBEC3A	210873_x_at	21.13	U03891	Hs.348983	apolipoprotein B mRNA editing enzyme, catalytic polypeptide-like 3A
SOD2	216841_s_at	21.03	X15132	Hs.487046	superoxide dismutase 2, mitochondrial
CALCRL	206331_at	20.96	NM_005795	Hs.470882	calcitonin receptor-like
STX11	210190_at	20.57	AF071504	Hs.118958	syntaxin 11
HERC6	219352_at	19.79	NM_017912	Hs.529317	hect domain and RLD 6
IL1RN	212657_s_at	19.4	U65590	Hs.81134	interleukin 1 receptor antagonist
MX1	202086_at	19.26	NM_002462	Hs.517307	myxovirus (influenza virus) resistance 1, interferon-inducible protein p78 (mouse)
PML	211012_s_at	18.98	BC000080	Hs.534573	promyelocytic leukemia ; hypothetical protein LOC161527
MGC5618	221477_s_at	18.92	BF575213		hypothetical protein MGC5618
IFI44L	204439_at	18.56	NM_006820	Hs.389724	interferon-induced protein 44-like
CD83	204440_at	18.36	NM_004233	Hs.484703	CD83 antigen (activated B lymphocytes, immunoglobulin superfamily)
IL6	205207_at	17.81	NM_000600	Hs.512234	interleukin 6 (interferon, beta 2)
CD40	205153_s_at	17.68	NM_001250	Hs.472860	CD40 antigen (TNF receptor superfamily member 5)
BIRC3	210538_s_at	17.58	U37546	Hs.127799	baculoviral IAP repeat-containing 3
IFIT5	203595_s_at	16.78	N47725	Hs.252839	interferon-induced protein with tetratricopeptide repeats 5
SERPINE2	212190_at	16.45	AL541302	Hs.38449	serpin peptidase inhibitor, clade E (nexin, plasminogen activator inhibitor type 1), member 2

STAT4	206118_at	16.25	NM_003151	Hs.80642	signal transducer and activator of transcription 4
SN	219519_s_at	16.08	NM_023068	Hs.31869	Sialoadhesin ; Sialoadhesin
IFIT3	204747_at	16.03	NM_001549	Hs.47338	interferon-induced protein with tetratricopeptide repeats 3
CRIM1	202551_s_at	15.74	BG546884	Hs.332847	cysteine rich transmembrane BMP regulator 1 (chordin-like)
TNFAIP3	202643_s_at	15.14	AI738896	Hs.211600	tumor necrosis factor, alpha-induced protein 3
IFI44	214453_s_at	14.59	NM_006417	Hs.82316	interferon-induced protein 44
CASP10	205467_at	14.46	NM_001230	Hs.5353	caspase 10, apoptosis-related cysteine peptidase
OAS1	205552_s_at	14.22	NM_002534	Hs.524760	2',5'-oligoadenylate synthetase 1, 40/46kDa
POU2F2	211771_s_at	14.16	BC006101	Hs.515429	POU domain, class 2, transcription factor 2 ; POU domain, class 2, transcription factor 2
EHD1	209037_s_at	14.03	AW182860	Hs.523774	EH-domain containing 1
IL1RN	212659_s_at	13.99	AW083357	Hs.81134	interleukin 1 receptor antagonist
PDE4B	203708_at	13.8	NM_002600	Hs.198072	phosphodiesterase 4B, cAMP-specific (phosphodiesterase E4 dunce homolog, Drosophila)
STAT2	217199_s_at	13.59	S81491	Hs.530595	signal transducer and activator of transcription 2, 113kDa
STAT1	209969_s_at	13.51	BC002704	Hs.470943	signal transducer and activator of transcription 1, 91kDa
OASL	210797_s_at	13.49	AF063612	Hs.118633	2'-5'-oligoadenylate synthetase-like
IFIH1	219209_at	13.48	NM_022168	Hs.163173	interferon induced with helicase C domain 1
TNFAIP3	202644_s_at	13.45	NM_006290	Hs.211600	tumor necrosis factor, alpha-induced protein 3
SP110	208392_x_at	13.22	NM_004510	Hs.145150	SP110 nuclear body protein
LOC51334	220014_at	13.19	NM_016644	Hs.157461	mesenchymal stem cell protein DSC54
ZC3H12A	218810_at	13.07	NM_025079	Hs.471918	zinc finger CCCH-type containing 12A
EHD1	209038_s_at	13.07	AL579035	Hs.523774	EH-domain containing 1
IRF7	208436_s_at	13.06	NM_004030	Hs.166120	interferon regulatory factor 7
IL1RN	216243_s_at	12.99	BE563442	Hs.81134	interleukin 1 receptor antagonist
G1P2	205483_s_at	12.98	NM_005101	Hs.458485	interferon, alpha-inducible protein (clone IFI-15K)
LILRB2	210225_x_at	12.86	AF009635	Hs.534386	leukocyte immunoglobulin-like receptor, subfamily B (with TM and ITIM domains), member 2
NFKBIA	201502_s_at	12.8	AI078167	Hs.81328	nuclear factor of kappa light polypeptide gene enhancer in B-cells inhibitor, alpha
IL10RA	204912_at	12.77	NM_001558	Hs.504035	interleukin 10 receptor, alpha
MX2	204994_at	12.5	NM_002463	Hs.926	myxovirus (influenza virus) resistance 2 (mouse)
TNFSF10	202687_s_at	12.39	U57059	Hs.478275	tumor necrosis factor (ligand) superfamily, member 10
TAP1	202307_s_at	12.19	NM_000593	Hs.352018	transporter 1, ATP-binding cassette, sub-family B (MDR/TAP)
DDX58	218943_s_at	12.18	NM_014314	Hs.190622	DEAD (Asp-Glu-Ala-Asp) box polypeptide 58
PBEF1	217739_s_at	12.1	NM_005746	Hs.489615	pre-B-cell colony enhancing factor 1
IFIT2	217502_at	11.81	BE888744	Hs.437609	interferon-induced protein with tetratricopeptide repeats 2
PTX3	206157_at	11.78	NM_002852	Hs.127657	pentraxin-related gene, rapidly induced by IL-1 beta
BTG2	201235_s_at	11.63	BG339064	Hs.519162	BTG family, member 2
OASL	205660_at	11.59	NM_003733	Hs.118633	2'-5'-oligoadenylate synthetase-like
GBP1	202270_at	11.56	NM_002053	Hs.62661	guanylate binding protein 1, interferon-inducible, 67kDa
TNFSF10	202688_at	11.5	NM_003810	Hs.478275	tumor necrosis factor (ligand) superfamily, member 10
EHD1	222221_x_at	11.43	AY007161	Hs.523774	EH-domain containing 1

NCF1	214084_x_at	11.4	AW072388	Hs.551786	neutrophil cytosolic factor 1 (47kDa, chronic granulomatous disease, autosomal 1)
TNFSF10	214329_x_at	11.21	AW474434	Hs.478275	Tumor necrosis factor (ligand) superfamily, member 10
KYNU	217388_s_at	11.04	D55639	Hs.470126	kynureninase (L-kynurenine hydrolase)
NCF1	204961_s_at	10.9	NM_000265	Hs.551786	neutrophil cytosolic factor 1 (47kDa, chronic granulomatous disease, autosomal 1)
ICAM1	202637_s_at	10.88	AI608725	Hs.515126	intercellular adhesion molecule 1 (CD54), human rhinovirus receptor
BRDG1	220059_at	10.7	NM_012108	Hs.435579	BCR downstream signaling 1
BTG2	201236_s_at	10.65	NM_006763	Hs.519162	BTG family, member 2
SAT	210592_s_at	10.35	M55580	Hs.28491	spermidine/spermine N1-acetyltransferase
RSAD2	213797_at	10.32	AI337069	Hs.17518	radical S-adenosyl methionine domain containing 2
TFAP2A	204653_at	10.27	BF343007	Hs.519880	transcription factor AP-2 alpha (activating enhancer binding protein 2 alpha)
C6orf145	212923_s_at	10.06	AK024828	Hs.484500	chromosome 6 open reading frame 145
CD40	215346_at	9.907	BF664114	Hs.472860	CD40 antigen (TNF receptor superfamily member 5)
PDGFRL	205226_at	9.901	NM_006207	Hs.458573	platelet-derived growth factor receptor-like
CD44	204490_s_at	9.843	M24915	Hs.502328	CD44 antigen (homing function and Indian blood group system)
C3AR1	209906_at	9.809	U62027	Hs.527839	complement component 3a receptor 1
GBP2	202748_at	9.758	NM_004120	Hs.386567	guanylate binding protein 2, interferon-inducible ; guanylate binding protein 2, interferon-inducible
IFI16	208965_s_at	9.757	BG256677	Hs.380250	interferon, gamma-inducible protein 16
PML	206503_x_at	9.7	NM_002675	Hs.526464	promyelocytic leukemia
OAS2	204972_at	9.665	NM_016817	Hs.414332	2'-5'-oligoadenylate synthetase 2, 69/71kDa
OAS1	202869_at	9.637	NM_016816	Hs.524760	2',5'-oligoadenylate synthetase 1, 40/46kDa
PLAUR	211924_s_at	9.52	AY029180	Hs.466871	plasminogen activator, urokinase receptor ; plasminogen activator, urokinase receptor
RRBP1	201206_s_at	9.507	NM_004587	Hs.472213	ribosome binding protein 1 homolog 180kDa (dog)
KCNJ2	206765_at	9.502	AF153820	Hs.1547	potassium inwardly-rectifying channel, subfamily J, member 2
KYNU	210663_s_at	9.336	BC000879	Hs.470126	kynureninase (L-kynurenine hydrolase)
CD44	212014_x_at	9.241	AI493245	Hs.502328	CD44 antigen (homing function and Indian blood group system)
CCR7	206337_at	9.228	NM_001838	Hs.370036	chemokine (C-C motif) receptor 7 ; chemokine (C-C motif) receptor 7
HMOX1	203665_at	9.185	NM_002133	Hs.517581	heme oxygenase (decycling) 1
MAFF	36711_at	9.163	AL021977	Hs.517617	v-maf musculoaponeurotic fibrosarcoma oncogene homolog F (avian)
SP110	208012_x_at	9.085	NM_004509	Hs.145150	SP110 nuclear body protein
CKIP-1	218223_s_at	9.047	NM_016274	Hs.438824	CK2 interacting protein 1; HQ0024c protein
MARCKS	201670_s_at	9.032	M68956	Hs.519909	myristoylated alanine-rich protein kinase C substrate
TNFAIP2	202510_s_at	9.004	NM_006291	Hs.525607	tumor necrosis factor, alpha-induced protein 2
SGK	201739_at	8.977	NM_005627	Hs.296323	serum/glucocorticoid regulated kinase
PSCDBP	209606_at	8.908	L06633	Hs.270	pleckstrin homology, Sec7 and coiled-coil domains, binding protein
PDE4B	211302_s_at	8.653	L20966	Hs.198072	phosphodiesterase 4B, cAMP-specific (phosphodiesterase E4 dunce homolog, Drosophila)
SAMD9	219691_at	8.348	NM_017654	Hs.65641	sterile alpha motif domain containing 9
SP110	209761_s_at	8.325	AA969194	Hs.145150	SP110 nuclear body protein
CCL5	1405_i_at	8.318	M21121	Hs.514821	chemokine (C-C motif) ligand 5
TRIM22	213293_s_at	8.204	AA083478	Hs.501778	tripartite motif-containing 22

PBEF1	217738_at	8.202	BF575514	Hs.489615	pre-B-cell colony enhancing factor 1
OAS3	218400_at	8.17	NM_006187	Hs.528634	2'-5'-oligoadenylate synthetase 3, 100kDa
SART2	218854_at	8.146	NM_013352	Hs.486292	squamous cell carcinoma antigen recognized by T cells 2
PML	211013_x_at	8.089	AF230411	Hs.526464	promyelocytic leukemia
PLA2G7	206214_at	7.93	NM_005084	Hs.554780	phospholipase A2, group VII (platelet-activating factor acetylhydrolase, plasma)
BCL6	203140_at	7.913	NM_001706	Hs.478588	B-cell CLL/lymphoma 6 (zinc finger protein 51) ; B-cell CLL/lymphoma 6 (zinc finger protein 51)
PELI1	218319_at	7.878	NM_020651	Hs.7886	pellino homolog 1 (Drosophila)
LY6E	202145_at	7.849	NM_002346	Hs.521903	lymphocyte antigen 6 complex, locus E
IFITM1	201601_x_at	7.848	NM_003641	Hs.458414	interferon induced transmembrane protein 1 (9-27)
FLJ11259	218627_at	7.788	NM_018370	Hs.525634	hypothetical protein FLJ11259
ACSL1	207275_s_at	7.738	NM_001995	Hs.406678	acyl-CoA synthetase long-chain family member 1
FLJ11286	53720_at	7.727	AI862559	Hs.175120	hypothetical protein FLJ11286
SAT	203455_s_at	7.721	NM_002970	Hs.28491	spermidine/spermine N1-acetyltransferase
SP110	209762_x_at	7.662	AF280094	Hs.145150	SP110 nuclear body protein
MARCKS	201668_x_at	7.624	AW163148	Hs.519909	myristoylated alanine-rich protein kinase C substrate
CD209	207278_s_at	7.596	NM_021155	Hs.278694	CD209 antigen
CD44	209835_x_at	7.594	BC004372	Hs.502328	CD44 antigen (homing function and Indian blood group system)
TNFAIP2	202509_s_at	7.557	AI862445	Hs.525607	tumor necrosis factor, alpha-induced protein 2
CRLF2	208303_s_at	7.522	NM_022148	Hs.287729	cytokine receptor-like factor 2
RND3	212724_at	7.424	BG054844	Hs.6838	Rho family GTPase 3
IBRDC3	213038_at	7.366	AL031602	Hs.546478	IBR domain containing 3
LILRB2	210146_x_at	7.254	AF004231	Hs.534386	leukocyte immunoglobulin-like receptor, subfamily B (with TM and ITIM domains), member 2
APOL6	219716_at	7.247	NM_030641	Hs.257352	apolipoprotein L, 6
SAMSN1	220330_s_at	7.094	NM_022136	Hs.473341	SAM domain, SH3 domain and nuclear localisation signals, 1
CREM	209967_s_at	6.976	D14826	Hs.200250	cAMP responsive element modulator
EHD1	209039_x_at	6.967	AF001434	Hs.523774	EH-domain containing 1
NFKB2	207535_s_at	6.949	NM_002502	Hs.73090	nuclear factor of kappa light polypeptide gene enhancer in B-cells 2 (p49/p100)
IFI16	206332_s_at	6.829	NM_005531	Hs.380250	interferon, gamma-inducible protein 16
IFI27	202411_at	6.774	NM_005532	Hs.532634	interferon, alpha-inducible protein 27
IFI16	208966_x_at	6.749	AF208043	Hs.380250	interferon, gamma-inducible protein 16
CCL5	204655_at	6.675	NM_002985	Hs.514821	chemokine (C-C motif) ligand 5 ; chemokine (C-C motif) ligand 5
PSD3	203355_s_at	6.671	NM_015310	Hs.434255	pleckstrin and Sec7 domain containing 3
SOD2	215078_at	6.646	AL050388	Hs.487046	superoxide dismutase 2, mitochondrial
TNIP1	207196_s_at	6.577	NM_006058	Hs.355141	TNFAIP3 interacting protein 1
RGL1	209568_s_at	6.564	AF186779	Hs.497148	ral guanine nucleotide dissociation stimulator-like 1
TRAF1	205599_at	6.559	NM_005658	Hs.531251	TNF receptor-associated factor 1
CD44	210916_s_at	6.557	AF098641	Hs.502328	CD44 antigen (homing function and Indian blood group system)
STAT1	AFFX-HUMIS	6.537	AFFX-HUMISC	Hs.470943	signal transducer and activator of transcription 1, 91kDa
CD44	217523_at	6.412	AV700298	Hs.502328	CD44 antigen (homing function and Indian blood group system)

NINJ1	203045_at	6.402	NM_004148	Hs.494457	ninjurin 1
MARCKS	201669_s_at	6.395	NM_002356	Hs.519909	myristoylated alanine-rich protein kinase C substrate
IFRG28	219684_at	6.297	NM_022147	Hs.43388	28kD interferon responsive protein
ACSL1	201963_at	6.292	NM_021122	Hs.406678	acyl-CoA synthetase long-chain family member 1
ZFP36L1	211962_s_at	6.262	BG250310	Hs.85155	zinc finger protein 36, C3H type-like 1
UGCG	204881_s_at	6.19	NM_003358	Hs.304249	UDP-glucose ceramide glucosyltransferase
LYN	210754_s_at	6.186	M79321	Hs.491767	v-yes-1 Yamaguchi sarcoma viral related oncogene homolog
TOR1B	209593_s_at	6.129	AF317129	Hs.252682	torsin family 1, member B (torsin B)
STAT1	AFFX-HUMIS	6.088	AFFX-HUMISC	Hs.470943	signal transducer and activator of transcription 1, 91kDa
WARS	200629_at	6.043	NM_004184	Hs.497599	tryptophanyl-tRNA synthetase
TNFRSF9	207536_s_at	6.028	NM_001561	Hs.193418	tumor necrosis factor receptor superfamily, member 9
PLSCR1	202446_s_at	6.027	AI825926	Hs.130759	phospholipid scramblase 1
CXCL12	209687_at	5.979	U19495	Hs.522891	chemokine (C-X-C motif) ligand 12 (stromal cell-derived factor 1)
SLAMF8	219385_at	5.976	NM_020125	Hs.438683	SLAM family member 8
AHR	202820_at	5.972	NM_001621	Hs.171189	aryl hydrocarbon receptor
PLEK	203470_s_at	5.931	AI433595	Hs.468840	pleckstrin
FTH1	214211_at	5.923	AA083483	Hs.558804	ferritin, heavy polypeptide 1
IER3	201631_s_at	5.905	NM_003897	Hs.76095	immediate early response 3
LYN	202626_s_at	5.902	NM_002350	Hs.491767	v-yes-1 Yamaguchi sarcoma viral related oncogene homolog
KYNU	204385_at	5.881	NM_003937	Hs.470126	kynureninase (L-kynurenine hydrolase)
NFKB1	209239_at	5.818	M55643	Hs.431926	nuclear factor of kappa light polypeptide gene enhancer in B-cells 1 (p105)
DUSP6	208891_at	5.801	BC003143	Hs.298654	dual specificity phosphatase 6
STAT1	AFFX-HUMIS	5.687	AFFX-HUMISC	Hs.470943	signal transducer and activator of transcription 1, 91kDa
TRIM34	221044_s_at	5.664	NM_021616	Hs.125300	tripartite motif-containing 34 ; tripartite motif-containing 6 and tripartite motif-containing 34
BCL3	204907_s_at	5.636	AI829875	Hs.31210	B-cell CLL/lymphoma 3
TRIM5	210705_s_at	5.622	AF220028	Hs.370515	tripartite motif-containing 5
THBS1	201108_s_at	5.618	BF055462	Hs.164226	thrombospondin 1
DUSP6	208892_s_at	5.599	BC003143	Hs.298654	dual specificity phosphatase 6
FCAR	207674_at	5.591	NM_002000	Hs.193122	Fc fragment of IgA, receptor for
PLEK	203471_s_at	5.555	NM_002664	Hs.468840	pleckstrin
PPP1R15A	202014_at	5.549	NM_014330	Hs.76556	protein phosphatase 1, regulatory (inhibitor) subunit 15A
OLIG2	213825_at	5.507	AA757419	Hs.176977	oligodendrocyte lineage transcription factor 2
MARCKS	213002_at	5.488	AA770596	Hs.519909	Myristoylated alanine-rich protein kinase C substrate
MMP14	217279_x_at	5.476	X83535	Hs.2399	matrix metallopeptidase 14 (membrane-inserted)
OPTN	202074_s_at	5.45	NM_021980	Hs.332706	optineurin
RAB6IP1	212561_at	5.396	AA349595	Hs.501857	RAB6 interacting protein 1
APOBEC3G	214995_s_at	5.391	BF508948	Hs.474853	apolipoprotein B mRNA editing enzyme, catalytic polypeptide-like 3G
HBEGF	203821_at	5.376	NM_001945	Hs.799	heparin-binding EGF-like growth factor
ME1	204059_s_at	5.36	NM_002395	Hs.21160	malic enzyme 1, NADP(+)-dependent, cytosolic

NFE2L3	204702_s_at	5.331	NM_004289	Hs.404741	nuclear factor (erythroid-derived 2)-like 3
VCAM1	203868_s_at	5.317	NM_001078	Hs.109225	vascular cell adhesion molecule 1
ETS2	201329_s_at	5.309	NM_005239	Hs.517296	v-ets erythroblastosis virus E26 oncogene homolog 2 (avian)
G0S2	213524_s_at	5.303	NM_015714	Hs.432132	G0/G1switch 2
ADAMDEC1	206134_at	5.25	NM_014479	Hs.521459	ADAM-like, decysin 1
CD44	204489_s_at	5.245	NM_000610	Hs.502328	CD44 antigen (homing function and Indian blood group system)
CPM	206100_at	5.196	NM_001874	Hs.484551	carboxypeptidase M
UBE2L6	201649_at	5.177	NM_004223	Hs.425777	ubiquitin-conjugating enzyme E2L 6
LYN	202625_at	5.118	AI356412	Hs.491767	v-yes-1 Yamaguchi sarcoma viral related oncogene homolog
PARP12	218543_s_at	5.056	NM_022750	Hs.12646	poly (ADP-ribose) polymerase family, member 12
JUN	201466_s_at	5.033	NM_002228	Hs.525704	v-jun sarcoma virus 17 oncogene homolog (avian)
OPTN	202073_at	5.032	AV757675	Hs.332706	optineurin
LILRB2	207697_x_at	5.016	NM_005874	Hs.534386	leukocyte immunoglobulin-like receptor, subfamily B (with TM and ITIM domains), member 2
CDKN1A	202284_s_at	4.978	NM_000389	Hs.370771	cyclin-dependent kinase inhibitor 1A (p21, Cip1)
ARL7	202208_s_at	4.965	BC001051	Hs.111554	ADP-ribosylation factor-like 7
CYLD	221903_s_at	4.956	BE046443	Hs.432993	cylindromatosis (turban tumor syndrome)
CHST2	203921_at	4.952	NM_004267	Hs.8786	carbohydrate (N-acetylglucosamine-6-O) sulfotransferase 2
IFITM1	214022_s_at	4.944	AA749101	Hs.458414	interferon induced transmembrane protein 1 (9-27)
PML	210362_x_at	4.928	AF230409	Hs.526464	promyelocytic leukemia
NPTX2	213479_at	4.916	U26662	Hs.3281	neuronal pentraxin II
ATF5	204999_s_at	4.906	BC005174	Hs.9754	activating transcription factor 5
HSD11B1	205404_at	4.887	NM_005525	Hs.195040	hydroxysteroid (11-beta) dehydrogenase 1
BID	204493_at	4.869	NM_001196	Hs.474150	BH3 interacting domain death agonist
TMEM45A	219410_at	4.822	NM_018004	Hs.126598	transmembrane protein 45A
UGCG	221765_at	4.798	AI378044	Hs.304249	UDP-glucose ceramide glucosyltransferase
N4BP1	32069_at	4.763	AB014515	Hs.558438	Nedd4 binding protein 1
HCK	208018_s_at	4.719	NM_002110	Hs.126521	hemopoietic cell kinase
OLIG2	213824_at	4.71	AI870776	Hs.176977	oligodendrocyte lineage transcription factor 2
BIRC4BP	206133_at	4.686	NM_017523	Hs.441975	XIAP associated factor-1
KIAA0286	212619_at	4.684	AW205215	Hs.533787	KIAA0286 protein
HIP1	205425_at	4.661	NM_005338	Hs.329266	huntingtin interacting protein 1
ST8SIA4	206925_at	4.648	NM_005668	Hs.308628	ST8 alpha-N-acetyl-neuraminide alpha-2,8-sialyltransferase 4
RELB	205205_at	4.637	NM_006509	Hs.307905	v-rel reticuloendotheliosis viral oncogene homolog B
MCTP1	220122_at	4.623	NM_024717	Hs.127412	multiple C2-domains with two transmembrane regions 1
EHD1	208112_x_at	4.617	NM_006795	Hs.523774	EH-domain containing 1
APOBEC3G	204205_at	4.584	NM_021822	Hs.474853	apolipoprotein B mRNA editing enzyme, catalytic polypeptide-like 3G
FLJ20035	218986_s_at	4.578	NM_017631	Hs.481141	hypothetical protein FLJ20035
TNFRSF1B	203508_at	4.578	NM_001066	Hs.256278	tumor necrosis factor receptor superfamily, member 1B
TCF4	212386_at	4.554	BF592782	Hs.200285	Transcription factor 4

PLSCR1	202430_s_at	4.515	NM_021105	Hs.130759	phospholipid scramblase 1
HERC5	219863_at	4.501	NM_016323	Hs.26663	hect domain and RLD 5
EPB41L3	212681_at	4.48	AI770004	Hs.213394	erythrocyte membrane protein band 4.1-like 3
RAB8B	219210_s_at	4.478	NM_016530	Hs.389733	RAB8B, member RAS oncogene family
CFLAR	208485_x_at	4.476	NM_003879	Hs.390736	CASP8 and FADD-like apoptosis regulator
CD44	212063_at	4.443	BE903880	Hs.502328	CD44 antigen (homing function and Indian blood group system)
PAX5	221969_at	4.433	BF510692	Hs.126365	Paired box gene 5 (B-cell lineage specific activator)
FGL2	204834_at	4.426	NM_006682	Hs.520989	fibrinogen-like 2
APOL1	209546_s_at	4.426	AF323540	Hs.114309	apolipoprotein L, 1
ATP2B1	209281_s_at	4.384	M95541	Hs.506276	ATPase, Ca++ transporting, plasma membrane 1
LOC54103	213142_x_at	4.349	AV700415	Hs.549342	hypothetical protein LOC54103
FLJ11286	218429_s_at	4.328	NM_018381	Hs.175120	hypothetical protein FLJ11286
INDO	210029_at	4.31	M34455	Hs.840	indoleamine-pyrrole 2,3 dioxygenase
C1orf24	217966_s_at	4.298	NM_022083	Hs.518662	chromosome 1 open reading frame 24
KITLG	207029_at	4.288	NM_000899	Hs.1048	KIT ligand
IFI35	209417_s_at	4.277	BC001356	Hs.50842	interferon-induced protein 35
EIF2AK2	204211_x_at	4.268	NM_002759	Hs.131431	eukaryotic translation initiation factor 2-alpha kinase 2
CFLAR	209939_x_at	4.26	AF005775	Hs.390736	CASP8 and FADD-like apoptosis regulator
DUSP1	201041_s_at	4.243	NM_004417	Hs.171695	dual specificity phosphatase 1
PDGFA	205463_s_at	4.232	NM_002607	Hs.376032	platelet-derived growth factor alpha polypeptide
OAS2	206553_at	4.204	NM_002535	Hs.414332	2'-5'-oligoadenylate synthetase 2, 69/71kDa
SNAI1	219480_at	4.204	NM_005985	Hs.48029	snail homolog 1 (Drosophila)
IL1A	210118_s_at	4.201	M15329	Hs.1722	interleukin 1, alpha
ATF5	204998_s_at	4.188	NM_012068	Hs.9754	activating transcription factor 5
CXCL9	203915_at	4.185	NM_002416	Hs.77367	chemokine (C-X-C motif) ligand 9
TCTEL1	201999_s_at	4.177	NM_006519	Hs.445999	t-complex-associated-testis-expressed 1-like 1
SPRED2	212466_at	4.171	AW138902	Hs.59332	sprouty-related, EVH1 domain containing 2
ATF3	202672_s_at	4.151	NM_001674	Hs.460	activating transcription factor 3
CD40	35150_at	4.121	X60592	Hs.472860	CD40 antigen (TNF receptor superfamily member 5)
CD209	207277_at	4.117	AF290886	Hs.278694	CD209 antigen
FGR	208438_s_at	4.103	NM_005248	Hs.1422	Gardner-Rasheed feline sarcoma viral (v-fgr) oncogene homolog
PANX1	204715_at	4.1	NM_015368	Hs.503584	pannexin 1
PIM1	209193_at	4.099	M24779	Hs.81170	pim-1 oncogene ; pim-1 oncogene
ADORA2A	205013_s_at	4.059	NM_000675	Hs.197029	adenosine A2a receptor
CASP7	207181_s_at	4.05	NM_001227	Hs.9216	caspase 7, apoptosis-related cysteine peptidase
JUN	201464_x_at	4.049	BG491844	Hs.525704	v-jun sarcoma virus 17 oncogene homolog (avian)
GAS7	202191_s_at	4.04	BE439987	Hs.462214	growth arrest-specific 7
N4BP1	204601_at	4.031	NM_014664	Hs.558438	Nedd4 binding protein 1
MYD88	209124_at	4.029	U70451	Hs.82116	myeloid differentiation primary response gene (88)

ATP1B1	201242_s_at	4.018	BC000006	Hs.291196	ATPase, Na+/K+ transporting, beta 1 polypeptide
ATP1B1	201243_s_at	4.006	NM_001677	Hs.291196	ATPase, Na+/K+ transporting, beta 1 polypeptide
PHF11	221816_s_at	4.004	BF055474	Hs.369039	PHD finger protein 11
FLJ38348	213294_at	4.002	AV755522	Hs.546523	Hypothetical protein FLJ38348
ISGF3G	203882_at	3.989	NM_006084	Hs.1706	interferon-stimulated transcription factor 3, gamma 48kDa
C6orf149	218561_s_at	3.987	NM_020408	Hs.387755	chromosome 6 open reading frame 149
STAT1	AFFX-HUMIS	3.973	AFFX-HUMISC	Hs.470943	signal transducer and activator of transcription 1, 91kDa
NFKBIE	203927_at	3.972	NM_004556	Hs.458276	nuclear factor of kappa light polypeptide gene enhancer in B-cells inhibitor, epsilon
LOC54103	222150_s_at	3.969	AK026747	Hs.549342	hypothetical protein LOC54103
LOC391020	216565_x_at	3.965	AL121994		similar to Interferon-induced transmembrane protein 3 (Interferon-inducible protein 1-8U)
AIM2	206513_at	3.957	NM_004833	Hs.281898	absent in melanoma 2
ENPP2	209392_at	3.941	L35594	Hs.190977	ectonucleotide pyrophosphatase/phosphodiesterase 2 (autotaxin)
STAT2	205170_at	3.926	NM_005419	Hs.530595	signal transducer and activator of transcription 2, 113kDa
CFLAR	211862_x_at	3.92	AF015451	Hs.390736	CASP8 and FADD-like apoptosis regulator
ME1	204058_at	3.92	AL049699	Hs.21160	Malic enzyme 1, NADP(+)-dependent, cytosolic
KIAA0286	212621_at	3.915	AB006624	Hs.533787	KIAA0286 protein
TRIM36	219736_at	3.907	NM_018700	Hs.519514	tripartite motif-containing 36
CASP5	207500_at	3.907	NM_004347	Hs.213327	caspase 5, apoptosis-related cysteine peptidase
ZFP36L1	211965_at	3.905	BE620915	Hs.85155	zinc finger protein 36, C3H type-like 1
CFLAR	211317_s_at	3.886	AF041461	Hs.390736	CASP8 and FADD-like apoptosis regulator
KMO	205306_x_at	3.877	AI074145		kynurenine 3-monooxygenase (kynurenine 3-hydroxylase)
CALM1	211984_at	3.862	AI653730	Hs.282410	calmodulin 1 (phosphorylase kinase, delta)
SLC15A3	219593_at	3.859	NM_016582	Hs.237856	solute carrier family 15, member 3
C1orf38	207571_x_at	3.858	NM_004848	Hs.10649	chromosome 1 open reading frame 38
IBRDC3	36564_at	3.826	W27419	Hs.546478	IBR domain containing 3
PHLDA1	217996_at	3.824	AA576961	Hs.558462	pleckstrin homology-like domain, family A, member 1
PLAUR	210845_s_at	3.818	U08839	Hs.466871	plasminogen activator, urokinase receptor
PHLDA1	217997_at	3.806	AI795908	Hs.558462	pleckstrin homology-like domain, family A, member 1
JUNB	201473_at	3.803	NM_002229	Hs.25292	jun B proto-oncogene
DOCK4	205003_at	3.796	NM_014705	Hs.118140	dedicator of cytokinesis 4
ICAM1	215485_s_at	3.752	AA284705	Hs.515126	intercellular adhesion molecule 1 (CD54), human rhinovirus receptor
FTH1	200748_s_at	3.752	NM_002032	Hs.558804	ferritin, heavy polypeptide 1
EPB41L3	206710_s_at	3.748	NM_012307	Hs.213394	erythrocyte membrane protein band 4.1-like 3
C1orf38	210785_s_at	3.748	AB035482	Hs.10649	chromosome 1 open reading frame 38
MT2A	212185_x_at	3.741	NM_005953	Hs.418241	metallothionein 2A
P2RY5	218589_at	3.717	NM_005767	Hs.123464	purinergic receptor P2Y, G-protein coupled, 5
SMAD7	204790_at	3.713	NM_005904	Hs.465087	SMAD, mothers against DPP homolog 7 (Drosophila)
SLC39A8	219869_s_at	3.709	NM_022154	Hs.288034	solute carrier family 39 (zinc transporter), member 8
NRIP1	202600_s_at	3.699	AI824012	Hs.155017	nuclear receptor interacting protein 1

TNFSF7	206508_at	3.679	NM_001252	Hs.501497	tumor necrosis factor (ligand) superfamily, member 7
UXS1	219675_s_at	3.668	NM_025076	Hs.469561	UDP-glucuronate decarboxylase 1
RGS16	209325_s_at	3.662	U94829	Hs.413297	regulator of G-protein signalling 16
CCR1	205099_s_at	3.659	NM_001295	Hs.301921	chemokine (C-C motif) receptor 1
KMO	211138_s_at	3.654	BC005297		kynurenine 3-monooxygenase (kynurenine 3-hydroxylase)
PPP1R15A	37028_at	3.651	U83981	Hs.76556	protein phosphatase 1, regulatory (inhibitor) subunit 15A
TFAP2A	204654_s_at	3.644	NM_003220	Hs.519880	transcription factor AP-2 alpha (activating enhancer binding protein 2 alpha)
CYP1B1	202435_s_at	3.637	AU154504	Hs.154654	cytochrome P450, family 1, subfamily B, polypeptide 1
DPY19L1	212792_at	3.622	AB020684	Hs.408623	dpy-19-like 1 (C. elegans)
SLC31A2	204204_at	3.622	NM_001860	Hs.24030	solute carrier family 31 (copper transporters), member 2
CASP1	206011_at	3.6	AI719655	Hs.2490	caspase 1, apoptosis-related cysteine peptidase (interleukin 1, beta, convertase)
BID	211725_s_at	3.584	BC005884	Hs.474150	BH3 interacting domain death agonist ; BH3 interacting domain death agonist
	215617_at	3.574	AU145711	Hs.224293	CDNA FLJ11754 fis, clone HEMBA1005588
PSTPIP2	219938_s_at	3.572	NM_024430	Hs.368623	proline-serine-threonine phosphatase interacting protein 2
CRIM1	202552_s_at	3.565	NM_016441	Hs.332847	cysteine rich transmembrane BMP regulator 1 (chordin-like)
TCF4	212382_at	3.551	BF433429	Hs.200285	Transcription factor 4
MTMR11	205076_s_at	3.547	NM_006697	Hs.425144	myotubularin related protein 11
GADD45B	207574_s_at	3.546	NM_015675	Hs.110571	growth arrest and DNA-damage-inducible, beta
LNK	203320_at	3.531	NM_005475	Hs.506784	lymphocyte adaptor protein
MTF1	205322_s_at	3.525	AW182367	Hs.471991	metal-regulatory transcription factor 1
LPPR4	213496_at	3.52	AW592563	Hs.13245	plasticity related gene 1
CYP1B1	202437_s_at	3.504	NM_000104	Hs.154654	cytochrome P450, family 1, subfamily B, polypeptide 1
IER5	218611_at	3.499	NM_016545	Hs.15725	immediate early response 5
ALAS1	205633_s_at	3.496	NM_000688	Hs.476308	aminolevulinate, delta-, synthase 1
KIAA0247	202181_at	3.491	NM_014734	Hs.440025	KIAA0247
NAB2	212803_at	3.477	BF337329	Hs.159223	NGFI-A binding protein 2 (EGR1 binding protein 2)
ZCCHC2	219062_s_at	3.477	NM_017742	Hs.114191	zinc finger, CCHC domain containing 2
WSB1	201295_s_at	3.474	BF111821	Hs.446017	WD repeat and SOCS box-containing 1
CALM1	211985_s_at	3.469	AI653730	Hs.282410	calmodulin 1 (phosphorylase kinase, delta)
RAFTLIN	212646_at	3.466	D42043	Hs.98910	raft-linking protein
QPCT	205174_s_at	3.462	NM_012413	Hs.79033	glutaminyl-peptide cyclotransferase (glutaminyl cyclase)
RTN1	210222_s_at	3.461	BC000314	Hs.368626	reticulon 1
BHLHB2	201169_s_at	3.453	BG326045	Hs.171825	basic helix-loop-helix domain containing, class B, 2
LPXN	216250_s_at	3.447	X77598	Hs.125474	leupaxin
GPR35	210264_at	3.429	AF089087	Hs.239891	G protein-coupled receptor 35
FGD2	215602_at	3.426	AK024456	Hs.509664	FYVE, RhoGEF and PH domain containing 2
CTNND1	208862_s_at	3.425	AW073672	Hs.166011	catenin (cadherin-associated protein), delta 1
PPARD	37152_at	3.423	L07592	Hs.485196	peroxisome proliferative activated receptor, delta
SKIL	217591_at	3.415	BF725121	Hs.536655	SKI-like

S100A12	205863_at	3.399	NM_005621	Hs.19413	S100 calcium binding protein A12 (calgranulin C) ; S100 calcium binding protein A12 (calgranulin C)
WTAP	210285_x_at	3.382	BC000383	Hs.446091	Wilms tumor 1 associated protein
THBD	203888_at	3.377	NM_000361	Hs.2030	thrombomodulin
HIVEP2	212641_at	3.366	AL023584	Hs.510172	human immunodeficiency virus type I enhancer binding protein 2
ATP2B1	215716_s_at	3.359	L14561	Hs.506276	ATPase, Ca++ transporting, plasma membrane 1
EMR2	207610_s_at	3.358	NM_013447	Hs.531619	egf-like module containing, mucin-like, hormone receptor-like 2
HBEGF	38037_at	3.356	M60278	Hs.799	heparin-binding EGF-like growth factor
GAS7	207704_s_at	3.35	NM_003644	Hs.462214	growth arrest-specific 7
STAT1	200887_s_at	3.35	NM_007315	Hs.470943	signal transducer and activator of transcription 1, 91kDa
FAS	215719_x_at	3.318	X83493	Hs.244139	Fas (TNF receptor superfamily, member 6)
SLC39A8	209267_s_at	3.295	AB040120	Hs.288034	solute carrier family 39 (zinc transporter), member 8
PRDM1	217192_s_at	3.294	AL022067	Hs.436023	PR domain containing 1, with ZNF domain
RUNX1T1	205529_s_at	3.292	NM_004349	Hs.368431	runt-related transcription factor 1; translocated to, 1 (cyclin D-related)
ARL7	202206_at	3.275	AW450363	Hs.111554	ADP-ribosylation factor-like 7
SLC7A11	217678_at	3.266	AA488687	Hs.390594	solute carrier family 7, (cationic amino acid transporter, y+ system) member 11
BHLHB2	201170_s_at	3.258	NM_003670	Hs.171825	basic helix-loop-helix domain containing, class B, 2
TNFAIP8	208296_x_at	3.247	NM_014350	Hs.271955	tumor necrosis factor, alpha-induced protein 8
TUBB2	204141_at	3.238	NM_001069	Hs.512712	tubulin, beta 2
FMNL1	204789_at	3.221	NM_005892	Hs.100217	formin-like 1
ATP2C1	209934_s_at	3.219	AF225981	Hs.567267	ATPase, Ca++ transporting, type 2C, member 1
IFNGR2	201642_at	3.203	NM_005534	Hs.517240	interferon gamma receptor 2 (interferon gamma transducer 1)
SP100	202864_s_at	3.202	NM_003113	Hs.369056	nuclear antigen Sp100
THBD	203887_s_at	3.202	NM_000361	Hs.2030	thrombomodulin
SDC4	202071_at	3.201	NM_002999	Hs.252189	syndecan 4 (amphiglycan, ryudocan)
NR4A3	207978_s_at	3.197	NM_006981	Hs.279522	nuclear receptor subfamily 4, group A, member 3
IFITM3	212203_x_at	3.197	BF338947	Hs.374650	interferon induced transmembrane protein 3 (1-8U)
FEZ1	205973_at	3.19	NM_022549	Hs.224008	fasciculation and elongation protein zeta 1 (zygin I)
DTX4	212611_at	3.183	AV728526	Hs.558464	deltex 4 homolog (Drosophila)
CFLAR	210563_x_at	3.178	U97075	Hs.390736	CASP8 and FADD-like apoptosis regulator
PDE4B	222326_at	3.16	AW973834	Hs.198072	Phosphodiesterase 4B, cAMP-specific (phosphodiesterase E4 dunce homolog, Drosophila)
SLC16A3	202856_s_at	3.144	NM_004207	Hs.500761	solute carrier family 16 (monocarboxylic acid transporters), member 3
CSPG2	204619_s_at	3.144	BF590263	Hs.443681	chondroitin sulfate proteoglycan 2 (versican)
CLIC4	201559_s_at	3.125	AF109196	Hs.440544	chloride intracellular channel 4
ENPP4	204160_s_at	3.124	AW194947	Hs.54037	ectonucleotide pyrophosphatase/phosphodiesterase 4 (putative function)
CLIC4	221881_s_at	3.114	AI638420	Hs.440544	chloride intracellular channel 4
TCF4	213891_s_at	3.113	AI927067	Hs.200285	Transcription factor 4
ETS2	201328_at	3.113	AL575509	Hs.517296	v-ets erythroblastosis virus E26 oncogene homolog 2 (avian)
PPIF	201490_s_at	3.11	NM_005729	Hs.381072	peptidylprolyl isomerase F (cyclophilin F)
SLC7A11	209921_at	3.106	AB040875	Hs.390594	solute carrier family 7, (cationic amino acid transporter, y+ system) member 11

CASP1	211368_s_at	3.105	U13700	Hs.2490	caspase 1, apoptosis-related cysteine peptidase (interleukin 1, beta, convertase)
KLF9	203543_s_at	3.084	NM_001206	Hs.150557	Kruppel-like factor 9
MMP14	202827_s_at	3.073	AU149305	Hs.2399	matrix metallopeptidase 14 (membrane-inserted)
SLC25A28	221432_s_at	3.056	NM_031212	Hs.403790	solute carrier family 25, member 28 ; solute carrier family 25, member 28
SLC16A3	202855_s_at	3.044	AL513917	Hs.500761	solute carrier family 16 (monocarboxylic acid transporters), member 3
FKBP5	204560_at	3.032	NM_004117	Hs.558328	FK506 binding protein 5
CHMP5	218085_at	3.027	NM_015961	Hs.415534	chromatin modifying protein 5
CYP1B1	202436_s_at	3.02	AU144855	Hs.154654	cytochrome P450, family 1, subfamily B, polypeptide 1
PSD3	218613_at	3.015	NM_018422	Hs.434255	pleckstrin and Sec7 domain containing 3
MT1X	208581_x_at	3.01	NM_005952	Hs.374950	metallothionein 1X
LITAF	200706_s_at	3.004	NM_004862	Hs.459940	lipopolysaccharide-induced TNF factor
IFI44	214059_at	2.987	BE049439	Hs.82316	Interferon-induced protein 44
SLIT2	209897_s_at	2.984	AF055585	Hs.558436	slit homolog 2 (Drosophila)
RAP2C	218669_at	2.968	NM_021183	Hs.119889	RAP2C, member of RAS oncogene family
HIST2H2AA	214290_s_at	2.962	AI313324	Hs.530461	histone 2, H2aa
FTHP1	211628_x_at	2.952	J04755	Hs.453583	ferritin, heavy polypeptide pseudogene 1 ; ferritin, heavy polypeptide pseudogene 1
CFLAR	209508_x_at	2.939	AF005774	Hs.390736	CASP8 and FADD-like apoptosis regulator ; CASP8 and FADD-like apoptosis regulator
CKB	200884_at	2.932	NM_001823	Hs.173724	creatine kinase, brain
PEA15	200788_s_at	2.929	NM_003768	Hs.517216	phosphoprotein enriched in astrocytes 15
PITPNA	201192_s_at	2.928	NM_006224	Hs.429819	phosphatidylinositol transfer protein, alpha
SQRDL	217995_at	2.919	NM_021199	Hs.511251	sulfide quinone reductase-like (yeast)
TNFAIP8	210260_s_at	2.918	BC005352	Hs.271955	tumor necrosis factor, alpha-induced protein 8
KIAA0226	212733_at	2.908	AI798908	Hs.478868	KIAA0226
NAB1	208047_s_at	2.907	NM_005966	Hs.107474	NGFI-A binding protein 1 (EGR1 binding protein 1)
IRF2	203275_at	2.905	NM_002199	Hs.374097	interferon regulatory factor 2
GAS7	211067_s_at	2.9	BC006454	Hs.462214	growth arrest-specific 7 ; growth arrest-specific 7
DUSP5	209457_at	2.893	U16996	Hs.2128	dual specificity phosphatase 5
CHMP5	219356_s_at	2.888	NM_016410	Hs.415534	chromatin modifying protein 5
WSB1	201294_s_at	2.88	N24643	Hs.446017	WD repeat and SOCS box-containing 1
HIVEP2	212642_s_at	2.874	AL023584	Hs.510172	human immunodeficiency virus type I enhancer binding protein 2
SLC11A2	203124_s_at	2.874	NM_000617	Hs.505545	solute carrier family 11 (proton-coupled divalent metal ion transporters), member 2
MALT1	208309_s_at	2.871	NM_006785	Hs.180566	mucosa associated lymphoid tissue lymphoma translocation gene 1
MAP2K3	215498_s_at	2.864	AA780381	Hs.514012	mitogen-activated protein kinase kinase 3 ; mitogen-activated protein kinase kinase 3
NAB1	209272_at	2.857	AF045451	Hs.107474	NGFI-A binding protein 1 (EGR1 binding protein 1)
EBI2	205419_at	2.854	NM_004951	Hs.508545	Epstein-Barr virus induced gene 2 (lymphocyte-specific G protein-coupled receptor)
IRF1	202531_at	2.85	NM_002198	Hs.436061	interferon regulatory factor 1
MARCKSL1	200644_at	2.849	NM_023009	Hs.75061	MARCKS-like 1
MT1K	216336_x_at	2.847	AL031602	Hs.188518	Metallothionein 1M
G1P3	204415_at	2.842	NM_022873	Hs.523847	interferon, alpha-inducible protein (clone IFI-6-16)

CD80	207176_s_at	2.832	NM_005191	Hs.838	CD80 antigen (CD28 antigen ligand 1, B7-1 antigen)
RASSF4	49306_at	2.825	AI890191	Hs.522895	Ras association (RalGDS/AF-6) domain family 4
IKBKE	204549_at	2.818	NM_014002	Hs.321045	inhibitor of kappa light polypeptide gene enhancer in B-cells, kinase epsilon
PEA15	200787_s_at	2.813	BC002426	Hs.517216	phosphoprotein enriched in astrocytes 15
RGS16	209324_s_at	2.809	BF304996	Hs.413297	regulator of G-protein signalling 16
SSH1	221753_at	2.804	AI651213	Hs.199763	slingshot homolog 1 (Drosophila)
P2RX4	204088_at	2.801	NM_002560	Hs.321709	purinergic receptor P2X, ligand-gated ion channel, 4
SLC2A3	202499_s_at	2.799	NM_006931	Hs.419240	solute carrier family 2 (facilitated glucose transporter), member 3
WARS	200628_s_at	2.797	M61715	Hs.497599	tryptophanyl-tRNA synthetase
LAP3	217933_s_at	2.797	NM_015907	Hs.479264	leucine aminopeptidase 3
CASP1	211367_s_at	2.793	U13699	Hs.2490	caspase 1, apoptosis-related cysteine peptidase (interleukin 1, beta, convertase)
TANK	207616_s_at	2.784	NM_004180	Hs.132257	TRAF family member-associated NFKB activator
RAP2C	218668_s_at	2.781	NM_021183	Hs.119889	RAP2C, member of RAS oncogene family
PTPRE	221840_at	2.761	AA775177	Hs.558374	protein tyrosine phosphatase, receptor type, E
REL	206036_s_at	2.76	NM_002908	Hs.370620	v-rel reticuloendotheliosis viral oncogene homolog (avian)
IGFBP4	201508_at	2.757	NM_001552	Hs.462998	insulin-like growth factor binding protein 4
DDIT4	202887_s_at	2.742	NM_019058	Hs.523012	DNA-damage-inducible transcript 4
GCLM	203925_at	2.737	NM_002061	Hs.315562	glutamate-cysteine ligase, modifier subunit
TRIM25	206911_at	2.732	NM_005082	Hs.528952	tripartite motif-containing 25
STK4	211085_s_at	2.73	Z25430	Hs.472838	serine/threonine kinase 4 ; serine/threonine kinase 4
CASP1	209970_x_at	2.726	M87507	Hs.2490	caspase 1, apoptosis-related cysteine peptidase (interleukin 1, beta, convertase)
KAL1	205206_at	2.719	NM_000216	Hs.521869	Kallmann syndrome 1 sequence
CLIC4	201560_at	2.718	NM_013943	Hs.440544	chloride intracellular channel 4
MAP2K3	207667_s_at	2.717	NM_002756	Hs.514012	mitogen-activated protein kinase kinase 3
USP12	215886_x_at	2.701	AF022790	Hs.562024	ubiquitin specific peptidase 12
PHACTR4	219235_s_at	2.7	NM_023923	Hs.225641	phosphatase and actin regulator 4
OGFRL1	219582_at	2.684	NM_024576	Hs.567296	opioid growth factor receptor-like 1
DAPP1	219290_x_at	2.68	NM_014395	Hs.436271	dual adaptor of phosphotyrosine and 3-phosphoinositides
	64488_at	2.675	AW003091	Hs.6217	CDNA FLJ46713 fis, clone TRACH3016885
PTPNS1	202897_at	2.672	AB023430	Hs.128846	protein tyrosine phosphatase, non-receptor type substrate 1
CASP1	211366_x_at	2.664	U13698	Hs.2490	caspase 1, apoptosis-related cysteine peptidase (interleukin 1, beta, convertase)
NFE2L2	201146_at	2.663	NM_006164	Hs.155396	nuclear factor (erythroid-derived 2)-like 2
STK17A	202693_s_at	2.637	AW194730	Hs.268887	serine/threonine kinase 17a (apoptosis-inducing)
PVRL2	203149_at	2.629	NM_002856	Hs.326371	poliovirus receptor-related 2 (herpesvirus entry mediator B)
PLCL1	205934_at	2.62	NM_006226	Hs.153322	phospholipase C-like 1
CFLAR	210564_x_at	2.619	AF009619	Hs.390736	CASP8 and FADD-like apoptosis regulator
PML ; LOC16152[illegible]	211014_s_at	2.618	AF230410	Hs.534573	promyelocytic leukemia ; hypothetical protein LOC161527
PNRC1	209034_at	2.618	AF279899	Hs.75969	proline-rich nuclear receptor coactivator 1
NQO1	201467_s_at	2.61	AI039874	Hs.406515	NAD(P)H dehydrogenase, quinone 1

pp9099	204436_at	2.599	NM_025201	Hs.458575	PH domain-containing protein
ADAR	201786_s_at	2.593	NM_001111	Hs.12341	adenosine deaminase, RNA-specific
BTG3	213134_x_at	2.59	AI765445	Hs.473420	BTG family, member 3
ABCC1	202804_at	2.585	AI539710	Hs.391464	ATP-binding cassette, sub-family C (CFTR/MRP), member 1
RAI17	212124_at	2.584	AF070622	Hs.193118	retinoic acid induced 17
HLA-E	200905_x_at	2.581	NM_005516	Hs.381008	major histocompatibility complex, class I, E
PIM2	204269_at	2.576	NM_006875	Hs.496096	pim-2 oncogene
SLA	203760_s_at	2.575	U44403	Hs.75367	Src-like-adaptor ; Src-like-adaptor
TNFRSF21	218856_at	2.568	NM_016629	Hs.443577	tumor necrosis factor receptor superfamily, member 21
DMXL2	212820_at	2.565	AB020663	Hs.511386	Dmx-like 2
PPIF	201489_at	2.564	BC005020	Hs.381072	peptidylprolyl isomerase F (cyclophilin F)
MGC10986	218600_at	2.563	NM_030576	Hs.555988	hypothetical protein MGC10986
RIT1	209882_at	2.554	AF084462	Hs.491234	Ras-like without CAAX 1
MGLL	211026_s_at	2.547	BC006230	Hs.277035	monoglyceride lipase ; monoglyceride lipase
RUNX3	204198_s_at	2.543	AA541630	Hs.170019	runt-related transcription factor 3
ECGF1	204858_s_at	2.539	NM_001953	Hs.546251	endothelial cell growth factor 1 (platelet-derived)
VASP	202205_at	2.536	NM_003370	Hs.515469	vasodilator-stimulated phosphoprotein
HIF1A	200989_at	2.533	NM_001530	Hs.509554	hypoxia-inducible factor 1, alpha subunit (basic helix-loop-helix transcription factor)
CHST11	219634_at	2.529	NM_018413	Hs.17569	carbohydrate (chondroitin 4) sulfotransferase 11
BLZF1	203840_at	2.522	NM_003666	Hs.494326	basic leucine zipper nuclear factor 1 (JEM-1)
SEC24A	212902_at	2.517	BE645231	Hs.211612	SEC24 related gene family, member A (S. cerevisiae)
BTG3	205548_s_at	2.515	NM_006806	Hs.473420	BTG family, member 3
SPHK1	219257_s_at	2.511	NM_021972	Hs.68061	sphingosine kinase 1
KCNQ3	206573_at	2.508	NM_004519	Hs.374023	potassium voltage-gated channel, KQT-like subfamily, member 3
	222303_at	2.5	AV700891		
ACVR2A	205327_s_at	2.488	NM_001616	Hs.470174	activin A receptor, type IIA
EPB41L3	211776_s_at	2.485	BC006141	Hs.213394	erythrocyte membrane protein band 4.1-like 3 ; erythrocyte membrane protein band 4.1-like 3
GTF2B	208066_s_at	2.485	NM_001514	Hs.481852	general transcription factor IIB ; general transcription factor IIB
MT1H	206461_x_at	2.482	NM_005951	Hs.438462	metallothionein 1H
LTB	207339_s_at	2.475	NM_002341	Hs.376208	lymphotoxin beta (TNF superfamily, member 3)
KCNN4	204401_at	2.468	NM_002250	Hs.10082	potassium intermediate/small conductance calcium-activated channel, subfamily N, member 4
LITAF	200704_at	2.461	AB034747	Hs.459940	lipopolysaccharide-induced TNF factor
PPARD	208044_s_at	2.46	NM_006238	Hs.485196	peroxisome proliferative activated receptor, delta
TCF4	203753_at	2.456	NM_003199	Hs.200285	transcription factor 4
DOK2	214054_at	2.449	AI828929	Hs.71215	docking protein 2, 56kDa
IKBKE	214398_s_at	2.433	AW340333	Hs.321045	inhibitor of kappa light polypeptide gene enhancer in B-cells, kinase epsilon
ABCC1	202805_s_at	2.431	NM_004996	Hs.391464	ATP-binding cassette, sub-family C (CFTR/MRP), member 1
C6orf32	206707_x_at	2.428	NM_015864	Hs.558439	chromosome 6 open reading frame 32
MOBK1B	201297_s_at	2.427	AK023321	Hs.196437	MOB1, Mps One Binder kinase activator-like 1B (yeast)

PSMB9	204279_at	2.423	NM_002800	Hs.381081	proteasome (prosome, macropain) subunit, beta type, 9 (large multifunctional peptidase 2)
LIMK2	217475_s_at	2.422	AC002073	Hs.474596	LIM domain kinase 2
PTPRC	212587_s_at	2.418	AI809341	Hs.192039	protein tyrosine phosphatase, receptor type, C
CCR1	205098_at	2.41	AI421071	Hs.301921	chemokine (C-C motif) receptor 1
NF2	211091_s_at	2.4	AF122828	Hs.187898	neurofibromin 2 (bilateral acoustic neuroma)
TCF7L2	212762_s_at	2.399	AI375916	Hs.501080	transcription factor 7-like 2 (T-cell specific, HMG-box)
EPHA7	206852_at	2.398	NM_004440	Hs.73962	EPH receptor A7
C6orf62	213875_x_at	2.396	BG252842	Hs.519930	chromosome 6 open reading frame 62
ROBO1	213194_at	2.395	BF059159	Hs.13640	roundabout, axon guidance receptor, homolog 1 (Drosophila)
RUNX3	204197_s_at	2.394	NM_004350	Hs.170019	runt-related transcription factor 3
SEC14L1	202082_s_at	2.393	AV748469	Hs.464184	SEC14-like 1 (S. cerevisiae)
TSPAN31	203227_s_at	2.391	NM_005981	Hs.50984	tetraspanin 31
SDCBP	200958_s_at	2.388	NM_005625	Hs.200804	syndecan binding protein (syntenin)
RHBDL6	219202_at	2.382	NM_024599	Hs.464157	rhomboid, veinlet-like 6 (Drosophila)
RPL35	211456_x_at	2.38	AF333388	Hs.449044	Similar to 60S ribosomal protein L35
BTG1	200920_s_at	2.379	AL535380	Hs.255935	B-cell translocation gene 1, anti-proliferative
SP100	210218_s_at	2.367	U36501	Hs.369056	nuclear antigen Sp100
TFEC	206715_at	2.364	NM_012252	Hs.125962	transcription factor EC
CSPG2	215646_s_at	2.356	R94644	Hs.443681	chondroitin sulfate proteoglycan 2 (versican) ; chondroitin sulfate proteoglycan 2 (versican)
GADD45B	209305_s_at	2.356	AF078077	Hs.110571	growth arrest and DNA-damage-inducible, beta
TNFRSF21	214581_x_at	2.351	BE568134	Hs.443577	tumor necrosis factor receptor superfamily, member 21
CDC42	208727_s_at	2.346	BC002711	Hs.467637	cell division cycle 42 (GTP binding protein, 25kDa)
ST3GAL2	205346_at	2.336	NM_006927	Hs.368611	ST3 beta-galactoside alpha-2,3-sialyltransferase 2
MTHFD2L	220346_at	2.336	NM_025001	Hs.535007	methylenetetrahydrofolate dehydrogenase (NADP+ dependent) 2-like
PRKAG2	218292_s_at	2.334	NM_016203	Hs.549162	protein kinase, AMP-activated, gamma 2 non-catalytic subunit
LILRB1	207104_x_at	2.333	NM_006669	Hs.149924	leukocyte immunoglobulin-like receptor, subfamily B (with TM and ITIM domains), member 1
YRDC	218647_s_at	2.332	NM_024640	Hs.301564	yrdC domain containing (E.coli)
CALM1	209563_x_at	2.33	BC000454	Hs.282410	calmodulin 1 (phosphorylase kinase, delta)
ECOP	208091_s_at	2.329	NM_030796	Hs.488307	EGFR-coamplified and overexpressed protein ; EGFR-coamplified and overexpressed protein
WTAP	203137_at	2.325	NM_004906	Hs.446091	Wilms tumor 1 associated protein
MCL1	200798_x_at	2.324	NM_021960	Hs.532826	myeloid cell leukemia sequence 1 (BCL2-related)
CALM1	200653_s_at	2.314	M27319	Hs.282410	calmodulin 1 (phosphorylase kinase, delta)
SLC2A3	222088_s_at	2.31	AA778684	Hs.419240	solute carrier family 2 (facilitated glucose transporter), member 3
RGS2	202388_at	2.305	NM_002923	Hs.78944	regulator of G-protein signalling 2, 24kDa
RCN1	201063_at	2.296	NM_002901	Hs.97887	reticulocalbin 1, EF-hand calcium binding domain
GPD2	210007_s_at	2.295	U36310	Hs.148266	glycerol-3-phosphate dehydrogenase 2 (mitochondrial)
DKFZp564K142	221553_at	2.293	AL136636	Hs.323562	implantation-associated protein
NAV3	204823_at	2.291	NM_014903	Hs.306322	neuron navigator 3
NAGK	218231_at	2.277	NM_017567	Hs.7036	N-acetylglucosamine kinase ; N-acetylglucosamine kinase

CD59	200985_s_at	2.277	NM_000611	Hs.278573	CD59 antigen p18-20 (antigen identified by monoclonal antibodies 16.3A5, EJ16, EJ30, EL32 and G344)
ZC3HAV1	220104_at	2.273	NM_020119	Hs.133512	zinc finger CCCH-type, antiviral 1
STAT3	208991_at	2.267	AA634272	Hs.463059	signal transducer and activator of transcription 3 (acute-phase response factor)
VEGF	211527_x_at	2.265	M27281	Hs.73793	vascular endothelial growth factor
SERPINB1	212268_at	2.255	NM_030666	Hs.381167	serpin peptidase inhibitor, clade B (ovalbumin), member 1
VRK2	205126_at	2.25	NM_006296	Hs.468623	vaccinia related kinase 2
LIMK2	202193_at	2.247	NM_005569	Hs.474596	LIM domain kinase 2
SPRED2	212458_at	2.247	H97931	Hs.59332	sprouty-related, EVH1 domain containing 2
GNA15	205349_at	2.245	NM_002068	Hs.73797	guanine nucleotide binding protein (G protein), alpha 15 (Gq class)
TRIM21	204804_at	2.239	NM_003141	Hs.532357	tripartite motif-containing 21
POLS	202466_at	2.231	NM_006999	Hs.481542	polymerase (DNA directed) sigma
MMP9	203936_s_at	2.227	NM_004994	Hs.297413	matrix metallopeptidase 9 (gelatinase B, 92kDa gelatinase, 92kDa type IV collagenase)
CDC73	218578_at	2.224	NM_024529	Hs.378996	cell division cycle 73, Paf1/RNA polymerase II complex component, homolog (S. cerevisiae)
TMPO	203432_at	2.221	AW272611	Hs.11355	thymopoietin
ABCA1	203504_s_at	2.22	NM_005502	Hs.429294	ATP-binding cassette, sub-family A (ABC1), member 1
LGALS9	203236_s_at	2.219	NM_009587	Hs.81337	lectin, galactoside-binding, soluble, 9 (galectin 9)
NAB1	211139_s_at	2.219	AF045452	Hs.107474	NGFI-A binding protein 1 (EGR1 binding protein 1)
SSH1	221752_at	2.218	AL041728	Hs.199763	Slingshot homolog 1 (Drosophila)
DAF	201925_s_at	2.217	NM_000574	Hs.527653	decay accelerating factor for complement (CD55, Cromer blood group system)
CYBB	203922_s_at	2.217	AI308863	Hs.292356	cytochrome b-245, beta polypeptide (chronic granulomatous disease)
PDLIM5	203243_s_at	2.214	NM_006457	Hs.480311	PDZ and LIM domain 5
PML	209640_at	2.211	M79462	Hs.526464	promyelocytic leukemia
CASP4	209310_s_at	2.208	U25804	Hs.138378	caspase 4, apoptosis-related cysteine peptidase
	213281_at	2.205	BE327172		
TANK	209451_at	2.203	U59863	Hs.132257	TRAF family member-associated NFKB activator
EMP3	203729_at	2.2	NM_001425	Hs.9999	epithelial membrane protein 3
CSF2RB	205159_at	2.199	AV756141	Hs.285401	colony stimulating factor 2 receptor, beta, low-affinity (granulocyte-macrophage)
VEGF	210512_s_at	2.197	AF022375	Hs.73793	vascular endothelial growth factor
ZBED1	203043_at	2.196	NM_004729	Hs.458462	zinc finger, BED-type containing 1
RTN1	203485_at	2.194	NM_021136	Hs.368626	reticulon 1
MCL1	200796_s_at	2.193	BF594446	Hs.532826	myeloid cell leukemia sequence 1 (BCL2-related)
USP12	213327_s_at	2.192	AI820101	Hs.42400	ubiquitin specific peptidase 12
MTF1	205323_s_at	2.192	NM_005955	Hs.471991	metal-regulatory transcription factor 1
CTSL	202087_s_at	2.188	NM_001912	Hs.418123	cathepsin L
HIVEP1	204512_at	2.185	NM_002114	Hs.128930	human immunodeficiency virus type I enhancer binding protein 1
STK3	204068_at	2.183	NM_006281	Hs.492333	serine/threonine kinase 3 (STE20 homolog, yeast)
LMNA	212086_x_at	2.179	AK026584	Hs.491359	lamin A/C
HRH1	205579_at	2.173	NM_000861	Hs.1570	histamine receptor H1
RASSF4	221578_at	2.172	AF260335	Hs.522895	Ras association (RalGDS/AF-6) domain family 4

SQSTM1	201471_s_at	2.169	NM_003900	Hs.529892	sequestosome 1
DDX3X	212514_x_at	2.166	R60068	Hs.380774	DEAD (Asp-Glu-Ala-Asp) box polypeptide 3, X-linked
CHST12	218927_s_at	2.166	NM_018641	Hs.213088	carbohydrate (chondroitin 4) sulfotransferase 12
C1orf24	217967_s_at	2.165	AF288391	Hs.518662	chromosome 1 open reading frame 24
OGFR	211512_s_at	2.164	AF172450	Hs.67896	opioid growth factor receptor
MOBK1B	201298_s_at	2.163	BC003398	Hs.196437	MOB1, Mps One Binder kinase activator-like 1B (yeast)
GRINA	212090_at	2.161	AL571424	Hs.339697	glutamate receptor, ionotropic, N-methyl D-asparate-associated protein 1 (glutamate binding)
TAPBP	208829_at	2.16	AF029750	Hs.370937	TAP binding protein (tapasin)
BLVRA	203771_s_at	2.155	AA740186	Hs.488143	biliverdin reductase A
IL6ST	204864_s_at	2.15	NM_002184	Hs.532082	interleukin 6 signal transducer (gp130, oncostatin M receptor)
DDX3X	212515_s_at	2.149	BG492602	Hs.380774	DEAD (Asp-Glu-Ala-Asp) box polypeptide 3, X-linked
ZFP36	201531_at	2.147	NM_003407	Hs.534052	zinc finger protein 36, C3H type, homolog (mouse)
HLA-G ; HLA-H	217436_x_at	2.143	M80469	Hs.512152	HLA-G histocompatibility antigen, class I, G ; major histocompatibility complex, class I, H (pseudogene)
C1orf106	219010_at	2.143	NM_018265	Hs.518997	chromosome 1 open reading frame 106
RIPK1	209941_at	2.142	U50062	Hs.519842	receptor (TNFRSF)-interacting serine-threonine kinase 1
JMJD3	41386_i_at	2.142	AB002344	Hs.553519	jumonji domain containing 3
EDEM1	203279_at	2.142	NM_014674	Hs.224616	ER degradation enhancer, mannosidase alpha-like 1
EHD4	209536_s_at	2.141	AF320070	Hs.143703	EH-domain containing 4
GADD45A	203725_at	2.138	NM_001924	Hs.80409	growth arrest and DNA-damage-inducible, alpha
RHOQ	212117_at	2.13	BF978689	Hs.549125	ras homolog gene family, member Q
SP100	202863_at	2.129	NM_003113	Hs.369056	nuclear antigen Sp100
TANK	210458_s_at	2.128	BC003388	Hs.132257	TRAF family member-associated NFKB activator
NRIP1	202599_s_at	2.125	NM_003489	Hs.155017	nuclear receptor interacting protein 1
NADK	215159_s_at	2.124	AI239732	Hs.456572	NAD kinase
PTGER4	204896_s_at	2.123	AI675173	Hs.199248	prostaglandin E receptor 4 (subtype EP4)
KIAA0226	212735_at	2.117	BF448041	Hs.478868	KIAA0226
RRM2	201890_at	2.111	BE966236	Hs.226390	ribonucleotide reductase M2 polypeptide
GMIP	218913_s_at	2.11	NM_016573	Hs.49427	GEM interacting protein
LOC93349	214791_at	2.109	AK023116	Hs.471582	hypothetical protein BC004921
SRD5A1	204675_at	2.109	NM_001047	Hs.552	steroid-5-alpha-reductase, alpha polypeptide 1 (3-oxo-5 alpha-steroid delta 4-dehydrogenase alpha 1)
HLA-A	215313_x_at	2.105	AA573862	Hs.181244	major histocompatibility complex, class I, A
MT1E	212859_x_at	2.104	BF217861	Hs.534330	metallothionein 1E (functional)
RIN3	60471_at	2.103	AA625133	Hs.326822	Ras and Rab interactor 3
WSB2	213734_at	2.1	BG260658	Hs.506985	WD repeat and SOCS box-containing 2
ARTS-1	210385_s_at	2.098	AF106037	Hs.436186	type 1 tumor necrosis factor receptor shedding aminopeptidase regulator
KIAA0674	76897_s_at	2.096	AA628140	Hs.522351	KIAA0674
VILL	209950_s_at	2.093	BC004300	Hs.103665	villin-like
UBE2D1	214590_s_at	2.093	AL545760	Hs.129683	ubiquitin-conjugating enzyme E2D 1 (UBC4/5 homolog, yeast)
FPRL1	210772_at	2.093	M88107	Hs.99855	formyl peptide receptor-like 1 ; formyl peptide receptor-like 1

HIP1	205426_s_at	2.091	U79734	Hs.329266	huntingtin interacting protein 1
AP1G1	203350_at	2.091	NM_001128	Hs.461253	adaptor-related protein complex 1, gamma 1 subunit
PLEKHF2	218640_s_at	2.089	NM_024613	Hs.29724	pleckstrin homology domain containing, family F (with FYVE domain) member 2
CTNND1	208407_s_at	2.085	NM_001331	Hs.166011	catenin (cadherin-associated protein), delta 1
STX12	212112_s_at	2.082	AI816243	Hs.523855	syntaxin 12
OGFR	202841_x_at	2.082	NM_007346	Hs.67896	opioid growth factor receptor
HPSE	219403_s_at	2.081	NM_006665	Hs.44227	heparanase
TRADD	1729_at	2.078	L41690	Hs.460996	TNFRSF1A-associated via death domain
BIRC4	206536_s_at	2.075	U32974	Hs.356076	baculoviral IAP repeat-containing 4
C6orf62	208809_s_at	2.074	AL136632	Hs.519930	chromosome 6 open reading frame 62
SLA	203761_at	2.074	NM_006748	Hs.75367	Src-like-adaptor ; Src-like-adaptor
CFLAR	211316_x_at	2.073	AF009616	Hs.390736	CASP8 and FADD-like apoptosis regulator
CD38	205692_s_at	2.072	NM_001775	Hs.479214	CD38 antigen (p45)
TMEM4	209797_at	2.072	BC001027	Hs.8752	transmembrane protein 4
PMAIP1	204285_s_at	2.071	AI857639	Hs.96	phorbol-12-myristate-13-acetate-induced protein 1
C6orf62	213872_at	2.062	BE465032	Hs.519930	Chromosome 6 open reading frame 62
MT1G	204745_x_at	2.061	NM_005950	Hs.433391	metallothionein 1G
NRP1	212298_at	2.061	BE620457	Hs.131704	neuropilin 1
JAK2	205841_at	2.061	NM_004972	Hs.434374	Janus kinase 2 (a protein tyrosine kinase)
CTNND1	211240_x_at	2.059	AB002382	Hs.166011	catenin (cadherin-associated protein), delta 1
SERPINB1	213572_s_at	2.059	AI554300	Hs.381167	serpin peptidase inhibitor, clade B (ovalbumin), member 1
NUP62	202153_s_at	2.058	NM_016553	Hs.467133	nucleoporin 62kDa
MALT1	210017_at	2.056	AF070528	Hs.180566	mucosa associated lymphoid tissue lymphoma translocation gene 1
NR4A2	204622_x_at	2.052	NM_006186	Hs.165258	nuclear receptor subfamily 4, group A, member 2
SEC24A	212900_at	2.051	AJ131244	Hs.211612	SEC24 related gene family, member A (S. cerevisiae)
RAB8A	208819_at	2.05	BC002977	Hs.5947	RAB8A, member RAS oncogene family
SLCO3A1	219229_at	2.048	NM_013272	Hs.311187	solute carrier organic anion transporter family, member 3A1
TRIM26	202702_at	2.046	NM_003449	Hs.485041	tripartite motif-containing 26
HPS5	204544_at	2.043	NM_007216	Hs.437599	Hermansky-Pudlak syndrome 5
ABCA1	203505_at	2.04	AF285167	Hs.429294	ATP-binding cassette, sub-family A (ABC1), member 1
CPD	201942_s_at	2.039	D85390	Hs.446079	carboxypeptidase D
EXOSC9	205061_s_at	2.039	NM_005033	Hs.91728	exosome component 9
TMEM4	202857_at	2.038	NM_014255	Hs.8752	transmembrane protein 4
FLJ13855	217750_s_at	2.036	NM_023079	Hs.514297	hypothetical protein FLJ13855
RAB13	202252_at	2.033	NM_002870	Hs.558375	RAB13, member RAS oncogene family
TESK1	204106_at	2.032	NM_006285	Hs.79358	testis-specific kinase 1
DPYSL2	200762_at	2.031	NM_001386	Hs.173381	dihydropyrimidinase-like 2
SNX11	220140_s_at	2.027	NM_013323	Hs.15827	sorting nexin 11
MTHFD2	201761_at	2.025	NM_006636	Hs.469030	methylenetetrahydrofolate dehydrogenase (NADP+ dependent) 2, methenyltetrahydrofolate cyclohydrolase

AGRN	212285_s_at	2.022	AW008051	Hs.273330	agrin
ENPP2	210839_s_at	2.02	D45421	Hs.190977	ectonucleotide pyrophosphatase/phosphodiesterase 2 (autotaxin)
GAS7	202192_s_at	2.016	NM_005890	Hs.462214	growth arrest-specific 7
NUP153	202097_at	2.015	NM_005124	Hs.121088	nucleoporin 153kDa
LSS	211019_s_at	2.013	D63807	Hs.517366	lanosterol synthase (2,3-oxidosqualene-lanosterol cyclase)
KPNA1	213741_s_at	2.012	BF575685	Hs.161008	karyopherin alpha 1 (importin alpha 5)
AGPAT3	219723_x_at	2.012	NM_020132	Hs.248785	1-acylglycerol-3-phosphate O-acyltransferase 3
SN	44673_at	2.01	N53555	Hs.31869	Sialoadhesin
PRKCH	218764_at	2.006	NM_024064	Hs.333907	protein kinase C, eta
ITGAV	202351_at	2.004	AI093579	Hs.436873	integrin, alpha V (vitronectin receptor, alpha polypeptide, antigen CD51)
TOP1	208900_s_at	2.004	AW025108	Hs.472737	topoisomerase (DNA) I
PLAGL1	209318_x_at	2.004	BG547855	Hs.444975	pleiomorphic adenoma gene-like 1
SPATA5L1	215694_at	2.002	AK022348	Hs.369657	spermatogenesis associated 5-like 1
JMJD2A	203204_s_at	0.499	BC002558	Hs.155983	jumonji domain containing 2A
C8orf52	218905_at	0.499	NM_017864	Hs.521693	chromosome 8 open reading frame 52
COX11	211727_s_at	0.498	BC005895	Hs.96530	COX11 homolog, cytochrome c oxidase assembly protein (yeast)
MYST1	221820_s_at	0.498	AK024102	Hs.533803	MYST histone acetyltransferase 1
EDD1	208883_at	0.497	BF515424	Hs.492445	E3 ubiquitin protein ligase, HECT domain containing, 1
GCAT	36475_at	0.496	Z97630	Hs.54609	glycine C-acetyltransferase (2-amino-3-ketobutyrate coenzyme A ligase)
FLJ20534	218646_at	0.495	NM_017867	Hs.406756	hypothetical protein FLJ20534
CGI-116	218628_at	0.495	NM_016053	Hs.405692	CGI-116 protein
CACYBP	201381_x_at	0.495	AF057356	Hs.508524	calcyclin binding protein
IDI1	204615_x_at	0.495	NM_004508	Hs.283652	isopentenyl-diphosphate delta isomerase 1
FADS1	208962_s_at	0.494	BE540552	Hs.503546	fatty acid desaturase 1
LOC81558	221249_s_at	0.494	NM_030802	Hs.514308	C/EBP-induced protein ; C/EBP-induced protein
EBNA1BP2	201323_at	0.493	NM_006824	Hs.346868	EBNA1 binding protein 2
FLJ14827	44065_at	0.492	AI937468	Hs.524762	hypothetical protein FLJ14827
DACH1	205471_s_at	0.492	AW772082	Hs.129452	dachshund homolog 1 (Drosophila)
GATM	203178_at	0.492	NM_001482	Hs.75335	glycine amidinotransferase (L-arginine:glycine amidinotransferase)
NY-REN-58	219644_at	0.492	NM_016122	Hs.279209	NY-REN-58 antigen
INPP5A	203006_at	0.49	NM_005539	Hs.523360	inositol polyphosphate-5-phosphatase, 40kDa
USP48	220079_s_at	0.49	NM_018391	Hs.467524	ubiquitin specific peptidase 48
AKAP10	213396_s_at	0.489	AA456929	Hs.462457	A kinase (PRKA) anchor protein 10
MPST	203524_s_at	0.488	NM_021126	Hs.248267	mercaptopyruvate sulfurtransferase
ST14	202005_at	0.487	NM_021978	Hs.504315	suppression of tumorigenicity 14 (colon carcinoma, matriptase, epithin)
KIAA0564	212946_at	0.487	AK025432	Hs.368282	KIAA0564 protein
PILRB	220954_s_at	0.487	NM_013440	Hs.530084	paired immunoglobin-like type 2 receptor beta
GGA2	214190_x_at	0.486	AI799984	Hs.460336	golgi associated, gamma adaptin ear containing, ARF binding protein 2
ZFR	201857_at	0.486	NM_016107	Hs.435231	zinc finger RNA binding protein

MT	213132_s_at	0.485	AL022237	Hs.349111	malonyl-CoA:acyl carrier protein transacylase, mitochondrial
OPN3	219032_x_at	0.483	NM_014322	Hs.409081	opsin 3 (encephalopsin, panopsin)
PRKACB	202742_s_at	0.483	NM_002731	Hs.487325	protein kinase, cAMP-dependent, catalytic, beta
PTPN18	213521_at	0.482	AW575379	Hs.516390	Protein tyrosine phosphatase, non-receptor type 18 (brain-derived)
R3HDM1	202754_at	0.481	NM_015361	Hs.412462	R3H domain containing 1
CBFA2T3	208056_s_at	0.481	NM_005187	Hs.513811	core-binding factor, runt domain, alpha subunit 2; translocated to, 3
MIR16	202593_s_at	0.479	NM_016641	Hs.512607	membrane interacting protein of RGS16
FAM21C	211068_x_at	0.479	BC006456	Hs.365286	family with sequence similarity 21, member C
	209478_at	0.479	U95006		
C1orf22	220926_s_at	0.479	NM_025191	Hs.523811	chromosome 1 open reading frame 22
ZFR	201856_s_at	0.478	BC000376	Hs.435231	zinc finger RNA binding protein
UNG	202330_s_at	0.478	NM_003362	Hs.191334	uracil-DNA glycosylase
EIF2C2	222294_s_at	0.477	AW971415	Hs.449415	Eukaryotic translation initiation factor 2C, 2
RRAGD	221523_s_at	0.477	AL138717	Hs.485938	Ras-related GTP binding D
PDCL3	219043_s_at	0.477	NM_024065	Hs.449206	phosducin-like 3 ; hypothetical protein FLJ12205
SMARCA2	217707_x_at	0.477	AI535683	Hs.298990	SWI/SNF related, matrix associated, actin dependent regulator of chromatin, subfamily a, member 2
CLNS1A	209143_s_at	0.476	AF005422	Hs.107393	chloride channel, nucleotide-sensitive, 1A ; chromosome 3 open reading frame 4
CACYBP	211761_s_at	0.475	BC005975	Hs.508524	calcyclin binding protein ; calcyclin binding protein
PRSS12	205515_at	0.475	NM_003619	Hs.445857	protease, serine, 12 (neurotrypsin, motopsin)
OPRS1	201692_at	0.474	NM_005866	Hs.522087	opioid receptor, sigma 1
EPRS	200842_s_at	0.473	AI475965	Hs.497788	glutamyl-prolyl-tRNA synthetase
LOC137886	212934_at	0.472	AI245523	Hs.155572	hypothetical protein LOC137886
SMARCA2	206542_s_at	0.471	AV725365	Hs.298990	SWI/SNF related, matrix associated, actin dependent regulator of chromatin, subfamily a, member 2
PDLIM2	219165_at	0.469	NM_021630	Hs.555972	PDZ and LIM domain 2 (mystique)
HHEX	204689_at	0.469	NM_001529	Hs.118651	hematopoietically expressed homeobox
6-Sep	212414_s_at	0.468	D50918	Hs.387255	septin 6 ; cytokine-like nuclear factor n-pac
EBP	202735_at	0.468	NM_006579	Hs.522636	emopamil binding protein (sterol isomerase)
PKP4	201927_s_at	0.467	BG292559	Hs.407580	plakophilin 4
MICAL1	218376_s_at	0.467	NM_022765	Hs.33476	microtubule associated monoxygenase, calponin and LIM domain containing 1
RAB11FIP2	203883_s_at	0.466	BG249608	Hs.173656	RAB11 family interacting protein 2 (class I)
HHEX	215933_s_at	0.464	Z21533	Hs.118651	hematopoietically expressed homeobox
RIMS3	204730_at	0.461	NM_014747	Hs.434924	regulating synaptic membrane exocytosis 3
HTRA2	211152_s_at	0.46	AF184911	Hs.558479	HtrA serine peptidase 2
ARHGEF6	209539_at	0.46	D25304	Hs.522795	Rac/Cdc42 guanine nucleotide exchange factor (GEF) 6
CORO1A	209083_at	0.46	U34690	Hs.415067	coronin, actin binding protein, 1A
GNB5	204000_at	0.459	NM_016194	Hs.155090	guanine nucleotide binding protein (G protein), beta 5
CTBP2	210835_s_at	0.459	AF222711	Hs.501345	C-terminal binding protein 2
EPRS	200843_s_at	0.458	NM_004446	Hs.497788	glutamyl-prolyl-tRNA synthetase
SLC19A1	211576_s_at	0.458	BC003068	Hs.84190	solute carrier family 19 (folate transporter), member 1

OSBPL1A	208158_s_at	0.458	NM_018030	Hs.370725	oxysterol binding protein-like 1A ; oxysterol binding protein-like 1A
FLJ22386	218394_at	0.457	NM_024589	Hs.459795	leucine zipper domain protein
MS4A6A	219666_at	0.456	NM_022349	Hs.523702	membrane-spanning 4-domains, subfamily A, member 6A
MS4A4A	219607_s_at	0.453	NM_024021	Hs.325960	membrane-spanning 4-domains, subfamily A, member 4
ERCC5	202414_at	0.453	NM_000123	Hs.258429	excision repair cross-complementing rodent repair deficiency, complementation group 5
DCPS	218774_at	0.452	NM_014026	Hs.504249	decapping enzyme, scavenger
MAC30	212282_at	0.45	BF038366	Hs.199695	hypothetical protein MAC30
PMS1	213677_s_at	0.45	BG434893	Hs.111749	PMS1 postmeiotic segregation increased 1 (S. cerevisiae)
C6orf111	212179_at	0.448	AW157501	Hs.520287	chromosome 6 open reading frame 111
C21orf59	218123_at	0.445	NM_017835	Hs.5811	chromosome 21 open reading frame 59
P2RY2	206277_at	0.445	NM_002564	Hs.339	purinergic receptor P2Y, G-protein coupled, 2
GGA2	213772_s_at	0.445	BF196572	Hs.460336	golgi associated, gamma adaptin ear containing, ARF binding protein 2
MID1IP1	218251_at	0.444	NM_021242	Hs.522605	MID1 interacting protein 1 (gastrulation specific G12-like (zebrafish))
VLDLR	209822_s_at	0.444	L22431	Hs.370422	very low density lipoprotein receptor
SMARCA2	212257_s_at	0.444	AW131754	Hs.298990	SWI/SNF related, matrix associated, actin dependent regulator of chromatin, subfamily a, member 2
	212764_at	0.443	AI806174		
PRG2	220798_x_at	0.441	NM_024888	Hs.546439	plasticity-related gene 2
C6orf210	219307_at	0.441	NM_020381	Hs.486095	chromosome 6 open reading frame 210
KPNB1	213803_at	0.44	BG545463	Hs.532793	Karyopherin (importin) beta 1
C14orf87	221932_s_at	0.44	AA133341	Hs.532683	chromosome 14 open reading frame 87
NTE	203718_at	0.44	NM_006702	Hs.511760	neuropathy target esterase
ATP2B4	212136_at	0.44	AW517686	Hs.343522	ATPase, Ca++ transporting, plasma membrane 4
CLGN	205830_at	0.438	NM_004362	Hs.86368	calmegin
ANKRD15	213005_s_at	0.438	D79994	Hs.493272	ankyrin repeat domain 15
SQLE	213562_s_at	0.437	BF979497	Hs.71465	squalene epoxidase
TOP2B	211987_at	0.436	NM_001068	Hs.475733	topoisomerase (DNA) II beta 180kDa
PYCARD	221666_s_at	0.436	BC004470	Hs.499094	PYD and CARD domain containing
CTBP2	201218_at	0.435	N23018	Hs.501345	C-terminal binding protein 2
SPFH1	202441_at	0.434	AL568449	Hs.150087	SPFH domain family, member 1
ITPR1	216944_s_at	0.432	U23850	Hs.443239	inositol 1,4,5-triphosphate receptor, type 1
KCNAB2	203402_at	0.431	AL520102	Hs.440497	potassium voltage-gated channel, shaker-related subfamily, beta member 2
TCFL5	204849_at	0.431	NM_006602	Hs.30696	transcription factor-like 5 (basic helix-loop-helix)
SCAMP1	212416_at	0.43	AV745949	Hs.482587	secretory carrier membrane protein 1
PFAS	213302_at	0.43	AL044326	Hs.88139	phosphoribosylformylglycinamidine synthase (FGAR amidotransferase)
ZNF238	212774_at	0.429	AJ223321	Hs.69997	zinc finger protein 238
ITGA4	205885_s_at	0.429	L12002	Hs.555880	integrin, alpha 4 (antigen CD49D, alpha 4 subunit of VLA-4 receptor)
WRB	202749_at	0.426	NM_004627	Hs.198308	tryptophan rich basic protein
SFXN3	217226_s_at	0.426	M95929	Hs.283844	sideroflexin 3
TBC1D5	201814_at	0.425	AI300084	Hs.475629	TBC1 domain family, member 5

MYB	204798_at	0.424	NM_005375	Hs.531941	v-myb myeloblastosis viral oncogene homolog (avian)
PKP4	201929_s_at	0.424	NM_003628	Hs.407580	plakophilin 4
MFNG	204153_s_at	0.423	NM_002405	Hs.517603	manic fringe homolog (Drosophila)
FLJ11712	219056_at	0.42	NM_024570	Hs.306291	hypothetical protein FLJ11712
PRKAR2B	203680_at	0.419	NM_002736	Hs.433068	protein kinase, cAMP-dependent, regulatory, type II, beta
PSIP1	209337_at	0.419	AF063020	Hs.493516	PC4 and SFRS1 interacting protein 1
SGK3	220038_at	0.419	NM_013257	Hs.380877	serum/glucocorticoid regulated kinase family, member 3
ST14	216905_s_at	0.416	U20428	Hs.504315	suppression of tumorigenicity 14 (colon carcinoma, matriptase, epithin)
PSAT1	220892_s_at	0.416	NM_021154	Hs.494261	phosphoserine aminotransferase 1
KIAA0433	203253_s_at	0.416	NM_015216	Hs.212046	KIAA0433 protein
ITGA4	205884_at	0.415	NM_000885	Hs.555880	integrin, alpha 4 (antigen CD49D, alpha 4 subunit of VLA-4 receptor)
BCL2	203685_at	0.412	NM_000633	Hs.150749	B-cell CLL/lymphoma 2
SAMM50	201569_s_at	0.411	NM_015380	Hs.505824	sorting and assembly machinery component 50 homolog (S. cerevisiae)
CARD9	220162_s_at	0.409	NM_022352	Hs.558530	caspase recruitment domain family, member 9
SPTBN1	212071_s_at	0.407	BE968833	Hs.503178	spectrin, beta, non-erythrocytic 1
TUBA1	212242_at	0.404	AL565074	Hs.75318	tubulin, alpha 1 (testis specific)
RPL10	221989_at	0.404	AW057781	Hs.401929	ribosomal protein L10
RASGRP2	214369_s_at	0.403	AI688812	Hs.99491	RAS guanyl releasing protein 2 (calcium and DAG-regulated)
CAT	211922_s_at	0.4	AY028632	Hs.502302	catalase ; catalase
ASAH1	210980_s_at	0.399	U47674	Hs.527412	N-acylsphingosine amidohydrolase (acid ceramidase) 1
GPR18	210279_at	0.397	AF261135	Hs.558333	G protein-coupled receptor 18
CXCR4	209201_x_at	0.397	L01639	Hs.421986	chemokine (C-X-C motif) receptor 4
JARID1B	201549_x_at	0.396	NM_006618	Hs.443650	Jumonji, AT rich interactive domain 1B (RBP2-like)
SPFH1	202444_s_at	0.395	NM_006459	Hs.150087	SPFH domain family, member 1
C14orf159	218298_s_at	0.392	NM_024952	Hs.309849	chromosome 14 open reading frame 159
EBP	213787_s_at	0.392	AV702405	Hs.522636	emopamil binding protein (sterol isomerase)
SMARCA2	206544_x_at	0.39	NM_003070	Hs.298990	SWI/SNF related, matrix associated, actin dependent regulator of chromatin, subfamily a, member 2
FLJ11127	214947_at	0.387	AF052146	Hs.155085	Hypothetical protein FLJ11127
FLJ10287	219130_at	0.383	NM_019083	Hs.440371	hypothetical protein FLJ10287
ASAH1	213702_x_at	0.375	AI934569	Hs.527412	N-acylsphingosine amidohydrolase (acid ceramidase) 1
DHRS9	219799_s_at	0.373	NM_005771	Hs.179608	dehydrogenase/reductase (SDR family) member 9
SLC29A1	201801_s_at	0.372	AF079117	Hs.25450	solute carrier family 29 (nucleoside transporters), member 1
MRPL33	203781_at	0.372	NM_004891	Hs.515879	mitochondrial ribosomal protein L33
PDCD4	202731_at	0.372	NM_014456	Hs.232543	programmed cell death 4 (neoplastic transformation inhibitor)
SLC29A1	201802_at	0.367	NM_004955	Hs.25450	solute carrier family 29 (nucleoside transporters), member 1
SFXN3	220974_x_at	0.365	NM_030971	Hs.283844	sideroflexin 3 ; sideroflexin 3
CXCR4	217028_at	0.363	AJ224869	Hs.421986	chemokine (C-X-C motif) receptor 4
QPRT	204044_at	0.363	NM_014298	Hs.513484	quinolinate phosphoribosyltransferase (nicotinate-nucleotide pyrophosphorylase (carboxylating))
TARBP1	202813_at	0.361	NM_005646	Hs.498115	TAR (HIV) RNA binding protein 1

MXI1	202364_at	0.361	NM_005962	Hs.501023	MAX interactor 1 ; MAX interactor 1
PSIP1	205961_s_at	0.358	NM_004682	Hs.493516	PC4 and SFRS1 interacting protein 1
PDCD4	202730_s_at	0.357	NM_014456	Hs.232543	programmed cell death 4 (neoplastic transformation inhibitor)
CXCR4	211919_s_at	0.357	AF348491	Hs.421986	chemokine (C-X-C motif) receptor 4 ; chemokine (C-X-C motif) receptor 4
HSU79274	204521_at	0.355	NM_013300	Hs.436618	protein predicted by clone 23733
COMMD8	218351_at	0.353	NM_017845	Hs.23956	COMM domain containing 8
PRKCA	213093_at	0.353	AI471375	Hs.531704	protein kinase C, alpha
HOMER3	204647_at	0.351	NM_004838	Hs.410683	homer homolog 3 (Drosophila)
HA-1	212873_at	0.35	BE349017	Hs.465521	minor histocompatibility antigen HA-1
RBL2	212331_at	0.344	X76061	Hs.513609	retinoblastoma-like 2 (p130)
EIF4EBP1	221539_at	0.344	AB044548	Hs.411641	eukaryotic translation initiation factor 4E binding protein 1
GM632	55872_at	0.341	AI493119	Hs.551552	KIAA1196 protein
IMPA2	203126_at	0.336	NM_014214	Hs.367992	inositol(myo)-1(or 4)-monophosphatase 2
C5orf13	201310_s_at	0.331	NM_004772	Hs.483067	chromosome 5 open reading frame 13
THY28	218491_s_at	0.331	NM_014174	Hs.13645	thymocyte protein thy28
LOC283768	213737_x_at	0.331	AI620911	Hs.146211	hypothetical LOC283768 ; similar to hypothetical protein
PDGFD	219304_s_at	0.329	NM_025208	Hs.352298	platelet derived growth factor D
SCD	200832_s_at	0.328	AB032261	Hs.558396	stearoyl-CoA desaturase (delta-9-desaturase)
CD1D	205789_at	0.321	NM_001766	Hs.1799	CD1D antigen, d polypeptide ; CD1D antigen, d polypeptide
C22orf9	212421_at	0.316	AB023147	Hs.369682	chromosome 22 open reading frame 9
C22orf9	217118_s_at	0.315	AK025608	Hs.369682	chromosome 22 open reading frame 9
DIPA	204610_s_at	0.311	NM_006848	Hs.66713	hepatitis delta antigen-interacting protein A
IRF8	204057_at	0.311	AI073984	Hs.137427	interferon regulatory factor 8 ; interferon regulatory factor 8
IDH1	201193_at	0.308	NM_005896	Hs.11223	isocitrate dehydrogenase 1 (NADP+), soluble
AGTPBP1	204500_s_at	0.305	NM_015239	Hs.494321	ATP/GTP binding protein 1
HOMER3	215489_x_at	0.294	AI871287	Hs.410683	homer homolog 3 (Drosophila)
C20orf103	219463_at	0.288	NM_012261	Hs.22920	chromosome 20 open reading frame 103
OBSL1	212775_at	0.275	AI978623	Hs.526594	obscurin-like 1
MS4A3	210254_at	0.268	L35848	Hs.99960	membrane-spanning 4-domains, subfamily A, member 3 (hematopoietic cell-specific)
RET	211421_s_at	0.264	M31213	Hs.350321	Ret proto-oncogene (multiple endocrine neoplasia and medullary thyroid carcinoma 1, Hirschsprung disease)
PDCD4	212593_s_at	0.223	N92498	Hs.232543	programmed cell death 4 (neoplastic transformation inhibitor)
