# Supplementary material for: Ganoderma lucidum Polysaccharides Induce Macrophage-Like Differentiation in Human Leukemia THP-1 Cells via Caspase and p53 Activation
Source: Evid Based Complement Alternat Med. 2011 Jan 4;2011:358717. doi: 10.1093/ecam/nep107 (PMC3135330; doi:10.1093/ecam/nep107)
Supplement: Supplementary file 2 [file 358717.f2.pdf]

supplementary Table 2. The differentially expressed genes in F3-treated THP-1 cells after 24 hours.

Gene Symbol	Chip ID	Fold Change	Genbank	UniGene	Description
CXCL13	205242_at	339.4	NM_006419	Hs.100431	chemokine (C-X-C motif) ligand 13 (B-cell chemoattractant)
GBP1	202269_x_at	140.9	BC002666	Hs.62661	guanylate binding protein 1, interferon-inducible, 67kDa ; guanylate binding protein 1, interferon-inducible, 67kDa
MMP1	204475_at	118.7	NM_002421	Hs.83169	matrix metallopeptidase 1 (interstitial collagenase)
EBI3	219424_at	117	NM_005755	Hs.501452	Epstein-Barr virus induced gene 3
ISG20	204698_at	100	NM_002201	Hs.459265	interferon stimulated exonuclease gene 20kDa
CCL8	214038_at	99.94	AI984980	Hs.271387	chemokine (C-C motif) ligand 8
CCL4	204103_at	94.93	NM_002984	Hs.75703	chemokine (C-C motif) ligand 4
IGFBP3	210095_s_at	79.85	M31159	Hs.450230	insulin-like growth factor binding protein 3
IGFBP3	212143_s_at	68.37	BF340228	Hs.450230	insulin-like growth factor binding protein 3
BCL3	204908_s_at	66.55	NM_005178	Hs.31210	B-cell CLL/lymphoma 3
CXCL10	204533_at	62.04	NM_001565	Hs.413924	chemokine (C-X-C motif) ligand 10
CCL2	216598_s_at	61.99	S69738	Hs.303649	chemokine (C-C motif) ligand 2
TDO2	205943_at	53.66	NM_005651	Hs.183671	tryptophan 2,3-dioxygenase
CXCL11	211122_s_at	51.98	AF002985	Hs.518814	chemokine (C-X-C motif) ligand 11
SAT	213988_s_at	50.6	BE971383	Hs.28491	spermidine/spermine N1-acetyltransferase
TLR7	220146_at	50.49	NM_016562	Hs.443036	toll-like receptor 7
MT1M	217546_at	49.46	R06655	Hs.188518	metallothionein 1M
SN	219519_s_at	47.06	NM_023068	Hs.31869	Sialoadhesin ; Sialoadhesin
IL7R	205798_at	45.81	NM_002185	Hs.362807	interleukin 7 receptor ; interleukin 7 receptor
USP18	219211_at	45.54	NM_017414	Hs.38260	ubiquitin specific peptidase 18
ADAMDEC1	206134_at	42.03	NM_014479	Hs.521459	ADAM-like, decysin 1
LAMP3	205569_at	38.96	NM_014398	Hs.518448	lysosomal-associated membrane protein 3
IL8	202859_x_at	34.51	NM_000584	Hs.624	interleukin 8
IL8	211506_s_at	33.97	AF043337	Hs.624	interleukin 8
NCF1	214084_x_at	32.46	AW072388	Hs.551786	neutrophil cytosolic factor 1 (47kDa, chronic granulomatous disease, autosomal 1)
NCF1	204961_s_at	31.27	NM_000265	Hs.551786	neutrophil cytosolic factor 1 (47kDa, chronic granulomatous disease, autosomal 1)
DUSP6	208893_s_at	27.48	BC005047	Hs.298654	dual specificity phosphatase 6
SLAMF8	219386_s_at	27.02	NM_020125	Hs.438683	SLAM family member 8
IFI44L	204439_at	25.9	NM_006820	Hs.389724	interferon-induced protein 44-like
BCL2A1	205681_at	25.48	NM_004049	Hs.227817	BCL2-related protein A1
MMP9	203936_s_at	24.88	NM_004994	Hs.297413	matrix metallopeptidase 9 (gelatinase B, 92kDa gelatinase, 92kDa type IV collagenase)
CCL3	205114_s_at	24.8	NM_002983	Hs.514107	chemokine (C-C motif) ligand 3 ; chemokine (C-C motif) ligand 3-like 1 ; chemokine (C-C motif) ligand 3-like 3
LY6E	202145_at	24.71	NM_002346	Hs.521903	lymphocyte antigen 6 complex, locus E
IFI27	202411_at	22.39	NM_005532	Hs.532634	interferon, alpha-inducible protein 27
GBP1	202270_at	22.13	NM_002053	Hs.62661	guanylate binding protein 1, interferon-inducible, 67kDa ; guanylate binding protein 1, interferon-inducible, 67kDa
IFIT1	203153_at	21.51	NM_001548	Hs.20315	interferon-induced protein with tetratricopeptide repeats 1
SLAMF7	219159_s_at	20.52	NM_021181	Hs.517265	SLAM family member 7
IL15RA	207375_s_at	19.62	NM_002189	Hs.524117	interleukin 15 receptor, alpha
CXCL11	210163_at	19.01	AF030514	Hs.518814	chemokine (C-X-C motif) ligand 11

RSAD2	213797_at	18.84	AI337069	Hs.17518	radical S-adenosyl methionine domain containing 2
MSC	209928_s_at	18.42	AF060154	Hs.442619	musculin (activated B-cell factor-1)
IL1B	205067_at	18.35	NM_000576	Hs.126256	interleukin 1, beta
CXCL1	204470_at	18.22	NM_001511	Hs.789	chemokine (C-X-C motif) ligand 1 (melanoma growth stimulating activity, alpha)
SOD2	215223_s_at	17.95	W46388	Hs.487046	superoxide dismutase 2, mitochondrial
ICAM1	202638_s_at	17.55	NM_000201	Hs.515126	intercellular adhesion molecule 1 (CD54), human rhinovirus receptor
HESX1	211267_at	17.34	U82811	Hs.171980	homeo box (expressed in ES cells) 1
HERC6	219352_at	17.31	NM_017912	Hs.529317	hect domain and RLD 6
MX1	202086_at	16.32	NM_002462	Hs.517307	myxovirus (influenza virus) resistance 1, interferon-inducible protein p78 (mouse)
LOC51334	220014_at	16.23	NM_016644	Hs.157461	mesenchymal stem cell protein DSC54
ISG20	33304_at	15.85	U88964	Hs.459265	interferon stimulated exonuclease gene 20kDa
TNFAIP6	206025_s_at	15.64	AW188198	Hs.437322	tumor necrosis factor, alpha-induced protein 6
OAS1	205552_s_at	15.53	NM_002534	Hs.524760	2',5'-oligoadenylate synthetase 1, 40/46kDa
TNFAIP6	206026_s_at	14.74	NM_007115	Hs.437322	tumor necrosis factor, alpha-induced protein 6
IFI44	214453_s_at	14.73	NM_006417	Hs.82316	interferon-induced protein 44
IFITM1	201601_x_at	14.45	NM_003641	Hs.458414	interferon induced transmembrane protein 1 (9-27)
MAFB	218559_s_at	14.19	NM_005461	Hs.169487	v-maf musculoaponeurotic fibrosarcoma oncogene homolog B (avian)
TNFSF10	202687_s_at	14.04	U57059	Hs.478275	tumor necrosis factor (ligand) superfamily, member 10 ; tumor necrosis factor (ligand) superfamily, member 10
IL1B	39402_at	13.72	M15330	Hs.126256	interleukin 1, beta
STAT1	209969_s_at	13.57	BC002704	Hs.470943	signal transducer and activator of transcription 1, 91kDa
SGK	201739_at	13.46	NM_005627	Hs.296323	serum/glucocorticoid regulated kinase
BIRC3	210538_s_at	13.19	U37546	Hs.127799	baculoviral IAP repeat-containing 3
CD14	201743_at	12.82	NM_000591	Hs.163867	CD14 antigen ; CD14 antigen
IFIT3	204747_at	12.46	NM_001549	Hs.47338	interferon-induced protein with tetratricopeptide repeats 3
IRF7	208436_s_at	11.9	NM_004030	Hs.166120	interferon regulatory factor 7
SOD2	216841_s_at	11.22	X15132	Hs.487046	superoxide dismutase 2, mitochondrial
ZFP36L1	211962_s_at	11.02	BG250310	Hs.85155	zinc finger protein 36, C3H type-like 1
G1P2	205483_s_at	10.99	NM_005101	Hs.458485	interferon, alpha-inducible protein (clone IFI-15K)
IFI16	208965_s_at	10.77	BG256677	Hs.380250	interferon, gamma-inducible protein 16
MGC4504	219270_at	10.7	NM_024111	Hs.155569	hypothetical protein MGC4504
FEZ1	203562_at	10.57	NM_005103	Hs.224008	fasciculation and elongation protein zeta 1 (zygin I)
CASP10	205467_at	10.55	NM_001230	Hs.5353	caspase 10, apoptosis-related cysteine peptidase
IL1RN	212657_s_at	10.47	U65590	Hs.81134	interleukin 1 receptor antagonist
IFI16	208966_x_at	10.22	AF208043	Hs.380250	interferon, gamma-inducible protein 16
ENPP2	209392_at	10.13	L35594	Hs.190977	ectonucleotide pyrophosphatase/phosphodiesterase 2 (autotaxin)
OAS1	202869_at	10.08	NM_016816	Hs.524760	2',5'-oligoadenylate synthetase 1, 40/46kDa
S100A12	205863_at	9.992	NM_005621	Hs.19413	S100 calcium binding protein A12 (calgranulin C) ; S100 calcium binding protein A12 (calgranulin C)
IFIT5	203595_s_at	9.986	N47725	Hs.252839	interferon-induced protein with tetratricopeptide repeats 5
MX2	204994_at	9.787	NM_002463	Hs.926	myxovirus (influenza virus) resistance 2 (mouse)
OAS2	204972_at	9.66	NM_016817	Hs.414332	2'-5'-oligoadenylate synthetase 2, 69/71kDa
ICAM1	202637_s_at	9.643	AI608725	Hs.515126	intercellular adhesion molecule 1 (CD54), human rhinovirus receptor
SLC2A6	220091_at	9.567	NM_017585	Hs.244378	solute carrier family 2 (facilitated glucose transporter), member 6

TNFSF10	214329_x_at	9.467	AW474434	Hs.478275	Tumor necrosis factor (ligand) superfamily, member 10 ; Tumor necrosis factor (ligand) superfamily, member 10
OASL	210797_s_at	9.312	AF063612	Hs.118633	2'-5'-oligoadenylate synthetase-like
TNFSF10	202688_at	9.277	NM_003810	Hs.478275	tumor necrosis factor (ligand) superfamily, member 10 ; tumor necrosis factor (ligand) superfamily, member 10
IFITM1	214022_s_at	9.265	AA749101	Hs.458414	interferon induced transmembrane protein 1 (9-27)
DDX58	218943_s_at	9.176	NM_014314	Hs.190622	DEAD (Asp-Glu-Ala-Asp) box polypeptide 58
OAS3	218400_at	9.105	NM_006187	Hs.528634	2'-5'-oligoadenylate synthetase 3, 100kDa
MMP14	217279_x_at	9.066	X83535	Hs.2399	matrix metallopeptidase 14 (membrane-inserted)
BRDG1	220059_at	8.89	NM_012108	Hs.435579	BCR downstream signaling 1
IFIH1	219209_at	8.735	NM_022168	Hs.163173	interferon induced with helicase C domain 1
IFIT2	217502_at	8.732	BE888744	Hs.437609	interferon-induced protein with tetratricopeptide repeats 2
TRIM22	213293_s_at	8.33	AA083478	Hs.501778	tripartite motif-containing 22
CXCL2	209774_x_at	8.066	M57731	Hs.75765	chemokine (C-X-C motif) ligand 2
TAP1	202307_s_at	8.028	NM_000593	Hs.352018	transporter 1, ATP-binding cassette, sub-family B (MDR/TAP)
IFI16	206332_s_at	7.894	NM_005531	Hs.380250	interferon, gamma-inducible protein 16
LOC391020	216565_x_at	7.893	AL121994		similar to Interferon-induced transmembrane protein 3 (Interferon-inducible protein 1-8U)
CXCL6	206336_at	7.825	NM_002993	Hs.164021	chemokine (C-X-C motif) ligand 6 (granulocyte chemotactic protein 2)
MGC5618	221477_s_at	7.616	BF575213		hypothetical protein MGC5618
PSCDBP	209606_at	7.615	L06633	Hs.270	pleckstrin homology, Sec7 and coiled-coil domains, binding protein
CCL7	208075_s_at	7.564	NM_006273	Hs.251526	chemokine (C-C motif) ligand 7 ; chemokine (C-C motif) ligand 7
IL1RN	212659_s_at	7.483	AW083357	Hs.81134	interleukin 1 receptor antagonist
BCL6	203140_at	7.406	NM_001706	Hs.478588	B-cell CLL/lymphoma 6 (zinc finger protein 51) ; B-cell CLL/lymphoma 6 (zinc finger protein 51)
OASL	205660_at	7.38	NM_003733	Hs.118633	2'-5'-oligoadenylate synthetase-like
STAT1	AFFX-HUMIS(	7.307	AFFX-HUMIS	Hs.470943	signal transducer and activator of transcription 1, 91kDa
SAMD9	219691_at	7.22	NM_017654	Hs.65641	sterile alpha motif domain containing 9
SP110	208392_x_at	7.021	NM_004510	Hs.145150	SP110 nuclear body protein
STAT1	AFFX-HUMIS(	6.967	AFFX-HUMIS	Hs.470943	signal transducer and activator of transcription 1, 91kDa
RRBP1	201206_s_at	6.933	NM_004587	Hs.472213	ribosome binding protein 1 homolog 180kDa (dog)
SLAMF8	219385_at	6.907	NM_020125	Hs.438683	SLAM family member 8
PDGFRL	205226_at	6.847	NM_006207	Hs.458573	platelet-derived growth factor receptor-like
DUSP6	208891_at	6.83	BC003143	Hs.298654	dual specificity phosphatase 6
SAT	210592_s_at	6.769	M55580	Hs.28491	spermidine/spermine N1-acetyltransferase
EPB41L3	212681_at	6.765	AI770004	Hs.213394	erythrocyte membrane protein band 4.1-like 3
IFI35	209417_s_at	6.704	BC001356	Hs.50842	interferon-induced protein 35
UBE2L6	201649_at	6.605	NM_004223	Hs.425777	ubiquitin-conjugating enzyme E2L 6
EPB41L3	206710_s_at	6.525	NM_012307	Hs.213394	erythrocyte membrane protein band 4.1-like 3
HSD11B1	205404_at	6.52	NM_005525	Hs.195040	hydroxysteroid (11-beta) dehydrogenase 1
HMOX1	203665_at	6.444	NM_002133	Hs.517581	heme oxygenase (decycling) 1
GALNAC4S-6ST	203066_at	6.41	NM_014863	Hs.287537	B cell RAG associated protein
C3AR1	209906_at	6.405	U62027	Hs.527839	complement component 3a receptor 1
CCL5	1405_i_at	6.388	M21121	Hs.514821	chemokine (C-C motif) ligand 5
TRIM34	221044_s_at	6.294	NM_021616	Hs.125300	tripartite motif-containing 34 ; tripartite motif-containing 6 and tripartite motif-containing 34
CTSL	202087_s_at	6.287	NM_001912	Hs.418123	cathepsin L

CCL5	204655_at	6.236	NM_002985	Hs.514821	chemokine (C-C motif) ligand 5 ; chemokine (C-C motif) ligand 5
NFKBIA	201502_s_at	6.184	AI078167	Hs.81328	nuclear factor of kappa light polypeptide gene enhancer in B-cells inhibitor, alpha
APOBEC3A	210873_x_at	6.171	U03891	Hs.348983	apolipoprotein B mRNA editing enzyme, catalytic polypeptide-like 3A
IL1RN	216243_s_at	6.148	BE563442	Hs.81134	interleukin 1 receptor antagonist
MAFF	36711_at	6.09	AL021977	Hs.517617	v-maf musculoaponeurotic fibrosarcoma oncogene homolog F (avian)
STAT1	AFFX-HUMIS(	6.05	AFFX-HUMIS	Hs.470943	signal transducer and activator of transcription 1, 91kDa
PELI1	218319_at	6.035	NM_020651	Hs.7886	pellino homolog 1 (Drosophila)
HERC5	219863_at	5.953	NM_016323	Hs.26663	hect domain and RLD 5
FXYD6	217897_at	5.931	NM_022003	Hs.504031	FXYD domain containing ion transport regulator 6
TNF	207113_s_at	5.916	NM_000594	Hs.241570	tumor necrosis factor (TNF superfamily, member 2)
GCH1	204224_s_at	5.764	NM_000161	Hs.86724	GTP cyclohydrolase 1 (dopa-responsive dystonia)
CD163	215049_x_at	5.659	Z22969	Hs.504641	CD163 antigen
ZFP36L1	211965_at	5.651	BE620915	Hs.85155	zinc finger protein 36, C3H type-like 1
SRD5A1	204675_at	5.601	NM_001047	Hs.552	steroid-5-alpha-reductase, alpha polypeptide 1 (3-oxo-5 alpha-steroid delta 4-dehydrogenase alpha 1)
BCL3	204907_s_at	5.567	AI829875	Hs.31210	B-cell CLL/lymphoma 3
ZC3H12A	218810_at	5.524	NM_025079	Hs.471918	zinc finger CCCH-type containing 12A
CD163	203645_s_at	5.522	NM_004244	Hs.504641	CD163 antigen
TNFAIP3	202644_s_at	5.489	NM_006290	Hs.211600	tumor necrosis factor, alpha-induced protein 3
SERPINA1	211429_s_at	5.456	AF119873	Hs.525557	serpin peptidase inhibitor, clade A (alpha-1 antiproteinase, antitrypsin), member 1
GBP2	202748_at	5.447	NM_004120	Hs.386567	guanylate binding protein 2, interferon-inducible ; guanylate binding protein 2, interferon-inducible
KYNU	217388_s_at	5.435	D55639	Hs.470126	kynureninase (L-kynurenine hydrolase)
TNFRSF9	207536_s_at	5.409	NM_001561	Hs.193418	tumor necrosis factor receptor superfamily, member 9
FLJ11286	53720_at	5.398	AI862559	Hs.175120	hypothetical protein FLJ11286
TNFAIP3	202643_s_at	5.384	AI738896	Hs.211600	tumor necrosis factor, alpha-induced protein 3
OAS2	206553_at	5.366	NM_002535	Hs.414332	2'-5'-oligoadenylate synthetase 2, 69/71kDa
PPP1R15A	202014_at	5.302	NM_014330	Hs.76556	protein phosphatase 1, regulatory (inhibitor) subunit 15A
NQO1	201467_s_at	5.266	AI039874	Hs.406515	NAD(P)H dehydrogenase, quinone 1
SP110	209762_x_at	5.259	AF280094	Hs.145150	SP110 nuclear body protein
RND3	212724_at	5.258	BG054844	Hs.6838	Rho family GTPase 3
LILRB2	207697_x_at	5.182	NM_005874	Hs.534386	leukocyte immunoglobulin-like receptor, subfamily B (with TM and ITIM domains), member 2
IL10RA	204912_at	5.174	NM_001558	Hs.504035	interleukin 10 receptor, alpha
LILRB2	210146_x_at	5.164	AF004231	Hs.534386	leukocyte immunoglobulin-like receptor, subfamily B (with TM and ITIM domains), member 2
PLSCR1	202446_s_at	5.125	AI825926	Hs.130759	phospholipid scramblase 1
AHR	202820_at	5.125	NM_001621	Hs.171189	aryl hydrocarbon receptor
FCAR	207674_at	5.001	NM_002000	Hs.193122	Fc fragment of IgA, receptor for
PLAUR	211924_s_at	4.93	AY029180	Hs.466871	plasminogen activator, urokinase receptor ; plasminogen activator, urokinase receptor
RGL1	209568_s_at	4.925	AF186779	Hs.497148	ral guanine nucleotide dissociation stimulator-like 1
BTG2	201236_s_at	4.882	NM_006763	Hs.519162	BTG family, member 2
EHD1	209038_s_at	4.84	AL579035	Hs.523774	EH-domain containing 1
EHBP1L1	221755_at	4.838	BG334196	Hs.502867	EH domain binding protein 1-like 1
SAT	203455_s_at	4.83	NM_002970	Hs.28491	spermidine/spermine N1-acetyltransferase
PIM1	209193_at	4.83	M24779	Hs.81170	pim-1 oncogene ; pim-1 oncogene

SN	44673_at	4.808	N53555	Hs.31869	Sialoadhesin
SERPINE2	212190_at	4.797	AL541302	Hs.38449	serpin peptidase inhibitor, clade E (nexin, plasminogen activator inhibitor type 1), member 2
CD83	204440_at	4.79	NM_004233	Hs.484703	CD83 antigen (activated B lymphocytes, immunoglobulin superfamily)
SP110	208012_x_at	4.786	NM_004509	Hs.145150	SP110 nuclear body protein
SPHK1	219257_s_at	4.746	NM_021972	Hs.68061	sphingosine kinase 1
SP110	209761_s_at	4.743	AA969194	Hs.145150	SP110 nuclear body protein
MARCKS	201670_s_at	4.663	M68956	Hs.519909	myristoylated alanine-rich protein kinase C substrate
IER3	201631_s_at	4.658	NM_003897	Hs.76095	immediate early response 3
PLSCR1	202430_s_at	4.633	NM_021105	Hs.130759	phospholipid scramblase 1
MARCKS	201668_x_at	4.619	AW163148	Hs.519909	myristoylated alanine-rich protein kinase C substrate
AIM2	206513_at	4.587	NM_004833	Hs.281898	absent in melanoma 2
LPXN	216250_s_at	4.543	X77598	Hs.125474	leupaxin
EHD1	209037_s_at	4.542	AW182860	Hs.523774	EH-domain containing 1
CYBB	203922_s_at	4.48	AI308863	Hs.292356	cytochrome b-245, beta polypeptide (chronic granulomatous disease)
FTH1	214211_at	4.476	AA083483	Hs.558804	ferritin, heavy polypeptide 1
ENPP2	210839_s_at	4.475	D45421	Hs.190977	ectonucleotide pyrophosphatase/phosphodiesterase 2 (autotaxin)
DUSP6	208892_s_at	4.451	BC003143	Hs.298654	dual specificity phosphatase 6
PDE4B	203708_at	4.448	NM_002600	Hs.198072	phosphodiesterase 4B, cAMP-specific (phosphodiesterase E4 dunce homolog, Drosophila)
WARS	200629_at	4.437	NM_004184	Hs.497599	tryptophanyl-tRNA synthetase
TRAF1	205599_at	4.43	NM_005658	Hs.531251	TNF receptor-associated factor 1
KMO	205306_x_at	4.42	AI074145		kynurenine 3-monooxygenase (kynurenine 3-hydroxylase)
ATF5	204999_s_at	4.417	BC005174	Hs.9754	activating transcription factor 5
HIST2H2AA	214290_s_at	4.403	AI313324	Hs.530461	histone 2, H2aa
BTG2	201235_s_at	4.395	BG339064	Hs.519162	BTG family, member 2
IFITM3	212203_x_at	4.391	BF338947	Hs.374650	interferon induced transmembrane protein 3 (1-8U)
CKIP-1	218223_s_at	4.388	NM_016274	Hs.438824	CK2 interacting protein 1; HQ0024c protein
HCK	208018_s_at	4.387	NM_002110	Hs.126521	hemopoietic cell kinase
CHST2	203921_at	4.378	NM_004267	Hs.8786	carbohydrate (N-acetylglucosamine-6-O) sulfotransferase 2
SART2	218854_at	4.366	NM_013352	Hs.486292	squamous cell carcinoma antigen recognized by T cells 2
INDO	210029_at	4.298	M34455	Hs.840	indoleamine-pyrrole 2,3 dioxygenase
FLJ11259	218627_at	4.249	NM_018370	Hs.525634	hypothetical protein FLJ11259
KMO	211138_s_at	4.204	BC005297		kynurenine 3-monooxygenase (kynurenine 3-hydroxylase)
EMR2	207610_s_at	4.199	NM_013447	Hs.531619	egf-like module containing, mucin-like, hormone receptor-like 2
SERPINA1	202833_s_at	4.199	NM_000295	Hs.525557	serpin peptidase inhibitor, clade A (alpha-1 antiproteinase, antitrypsin), member 1
DOCK4	205003_at	4.123	NM_014705	Hs.118140	dedicator of cytokinesis 4
PARP12	218543_s_at	4.116	NM_022750	Hs.12646	poly (ADP-ribose) polymerase family, member 12
PLA2G7	206214_at	4.113	NM_005084	Hs.554780	phospholipase A2, group VII (platelet-activating factor acetylhydrolase, plasma)
APOL6	219716_at	4.101	NM_030641	Hs.257352	apolipoprotein L, 6
IFRG28	219684_at	4.077	NM_022147	Hs.43388	28kD interferon responsive protein
SRD5A1	210959_s_at	4.059	AF113128	Hs.552	steroid-5-alpha-reductase, alpha polypeptide 1 (3-oxo-5 alpha-steroid delta 4-dehydrogenase alpha 1)
IGSF4	209031_at	4.034	AL519710	Hs.370510	Immunoglobulin superfamily, member 4
PLA2G4C	209785_s_at	4.02	AF065214	Hs.18858	phospholipase A2, group IVC (cytosolic, calcium-independent)

LY96	206584_at	4.018	NM_015364	Hs.69328	lymphocyte antigen 96
FAS	215719_x_at	4.003	X83493	Hs.244139	Fas (TNF receptor superfamily, member 6)
VCAM1	203868_s_at	3.996	NM_001078	Hs.109225	vascular cell adhesion molecule 1
ALOX5AP	204174_at	3.99	NM_001629	Hs.507658	arachidonate 5-lipoxygenase-activating protein
MMP14	202827_s_at	3.987	AU149305	Hs.2399	matrix metallopeptidase 14 (membrane-inserted)
GAS7	207704_s_at	3.965	NM_003644	Hs.462214	growth arrest-specific 7
PDGFA	205463_s_at	3.956	NM_002607	Hs.376032	platelet-derived growth factor alpha polypeptide
DDIT4	202887_s_at	3.948	NM_019058	Hs.523012	DNA-damage-inducible transcript 4
LYN	202626_s_at	3.947	NM_002350	Hs.491767	v-yes-1 Yamaguchi sarcoma viral related oncogene homolog
MARCKS	201669_s_at	3.932	NM_002356	Hs.519909	myristoylated alanine-rich protein kinase C substrate
RTN1	210222_s_at	3.922	BC000314	Hs.368626	reticulon 1
KYNU	210663_s_at	3.911	BC000879	Hs.470126	kynureninase (L-kynurenine hydrolase)
GPR35	210264_at	3.892	AF089087	Hs.239891	G protein-coupled receptor 35
ATF5	204998_s_at	3.89	NM_012068	Hs.9754	activating transcription factor 5
BIRC4BP	206133_at	3.887	NM_017523	Hs.441975	XIAP associated factor-1
CDKN1A	202284_s_at	3.887	NM_000389	Hs.370771	cyclin-dependent kinase inhibitor 1A (p21, Cip1)
OPTN	202074_s_at	3.886	NM_021980	Hs.332706	optineurin
RAPGEF1	204543_at	3.875	NM_005312	Hs.127897	Rap guanine nucleotide exchange factor (GEF) 1
TFAP2A	204653_at	3.826	BF343007	Hs.519880	transcription factor AP-2 alpha (activating enhancer binding protein 2 alpha)
GEM	204472_at	3.813	NM_005261	Hs.345139	GTP binding protein overexpressed in skeletal muscle
IBRDC3	36564_at	3.797	W27419	Hs.546478	IBR domain containing 3
FLJ20035	218986_s_at	3.787	NM_017631	Hs.481141	hypothetical protein FLJ20035
ECGF1	204858_s_at	3.776	NM_001953	Hs.546251	endothelial cell growth factor 1 (platelet-derived)
KCNJ2	206765_at	3.775	AF153820	Hs.1547	potassium inwardly-rectifying channel, subfamily J, member 2
LYN	210754_s_at	3.761	M79321	Hs.491767	v-yes-1 Yamaguchi sarcoma viral related oncogene homolog
IL6	205207_at	3.759	NM_000600	Hs.512234	interleukin 6 (interferon, beta 2)
IGSF4	209030_s_at	3.752	NM_014333	Hs.370510	immunoglobulin superfamily, member 4
CD40	215346_at	3.74	BF664114	Hs.472860	CD40 antigen (TNF receptor superfamily member 5)
EHD1	222221_x_at	3.734	AY007161	Hs.523774	EH-domain containing 1
C5R1	220088_at	3.729	NM_001736	Hs.2161	complement component 5 receptor 1 (C5a ligand)
ME1	204059_s_at	3.725	NM_002395	Hs.21160	malic enzyme 1, NADP(+)-dependent, cytosolic
PBEF1	217739_s_at	3.724	NM_005746	Hs.489615	pre-B-cell colony enhancing factor 1
VEGF	211527_x_at	3.71	M27281	Hs.73793	vascular endothelial growth factor
G1P3	204415_at	3.689	NM_022873	Hs.523847	interferon, alpha-inducible protein (clone IFI-6-16)
PTAFR	211661_x_at	3.66	M80436	Hs.46	platelet-activating factor receptor ; platelet-activating factor receptor
ALAS1	205633_s_at	3.654	NM_000688	Hs.476308	aminolevulinate, delta-, synthase 1
SLC31A1	203971_at	3.651	NM_001859	Hs.532315	solute carrier family 31 (copper transporters), member 1
P2RY5	218589_at	3.65	NM_005767	Hs.123464	purinergic receptor P2Y, G-protein coupled, 5
OLIG2	213825_at	3.64	AA757419	Hs.176977	oligodendrocyte lineage transcription factor 2
PSMB9	204279_at	3.629	NM_002800	Hs.381081	proteasome (prosome, macropain) subunit, beta type, 9 (large multifunctional peptidase 2)
EPB41L3	211776_s_at	3.627	BC006141	Hs.213394	erythrocyte membrane protein band 4.1-like 3 ; erythrocyte membrane protein band 4.1-like 3
CSPG2	204619_s_at	3.618	BF590263	Hs.443681	chondroitin sulfate proteoglycan 2 (versican)

ACSL1	207275_s_at	3.611	NM_001995	Hs.406678	acyl-CoA synthetase long-chain family member 1
NFKB2	207535_s_at	3.604	NM_002502	Hs.73090	nuclear factor of kappa light polypeptide gene enhancer in B-cells 2 (p49/p100)
CTSS	202902_s_at	3.59	NM_004079	Hs.181301	cathepsin S
NKG7	213915_at	3.588	NM_005601	Hs.10306	natural killer cell group 7 sequence
SAMSN1	220330_s_at	3.576	NM_022136	Hs.473341	SAM domain, SH3 domain and nuclear localisation signals, 1
STAT1	AFFX-HUMIS(	3.554	AFFX-HUMIS	Hs.470943	signal transducer and activator of transcription 1, 91kDa
LYN	202625_at	3.533	AI356412	Hs.491767	v-yes-1 Yamaguchi sarcoma viral related oncogene homolog
INHBE	210587_at	3.52	BC005161	Hs.279497	inhibin, beta E
APOL1	209546_s_at	3.477	AF323540	Hs.114309	apolipoprotein L, 1
CTSH	202295_s_at	3.46	NM_004390	Hs.148641	cathepsin H
VEGF	212171_x_at	3.429	H95344	Hs.73793	vascular endothelial growth factor
G0S2	213524_s_at	3.414	NM_015714	Hs.432132	G0/G1switch 2
KYNU	204385_at	3.413	NM_003937	Hs.470126	kynureninase (L-kynurenine hydrolase)
CASP1	206011_at	3.4	AI719655	Hs.2490	caspase 1, apoptosis-related cysteine peptidase (interleukin 1, beta, convertase)
TNFRSF1B	203508_at	3.397	NM_001066	Hs.256278	tumor necrosis factor receptor superfamily, member 1B
STAT1	200887_s_at	3.354	NM_007315	Hs.470943	signal transducer and activator of transcription 1, 91kDa
CTH	217127_at	3.327	AL354872	Hs.19904	cystathionase (cystathionine gamma-lyase)
GAS7	211067_s_at	3.321	BC006454	Hs.462214	growth arrest-specific 7 ; growth arrest-specific 7
ME1	204058_at	3.316	AL049699	Hs.21160	Malic enzyme 1, NADP(+)-dependent, cytosolic
FTH1	200748_s_at	3.288	NM_002032	Hs.558804	ferritin, heavy polypeptide 1
FLJ20273	218035_s_at	3.276	NM_019027	Hs.518727	RNA-binding protein
SLC2A3	222088_s_at	3.267	AA778684	Hs.419240	solute carrier family 2 (facilitated glucose transporter), member 3
APOBEC3G	214995_s_at	3.254	BF508948	Hs.474853	apolipoprotein B mRNA editing enzyme, catalytic polypeptide-like 3G
IDS	202438_x_at	3.244	BF346014	Hs.567240	iduronate 2-sulfatase (Hunter syndrome)
MTSS1	203037_s_at	3.243	NM_014751	Hs.336994	metastasis suppressor 1
TRIM5	210705_s_at	3.242	AF220028	Hs.370515	tripartite motif-containing 5
RELB	205205_at	3.238	NM_006509	Hs.307905	v-rel reticuloendotheliosis viral oncogene homolog B
PBEF1	217738_at	3.237	BF575514	Hs.489615	pre-B-cell colony enhancing factor 1
TCF7L2	212762_s_at	3.231	AI375916	Hs.501080	transcription factor 7-like 2 (T-cell specific, HMG-box)
ARL7	202206_at	3.219	AW450363	Hs.111554	ADP-ribosylation factor-like 7
PDE4B	211302_s_at	3.206	L20966	Hs.198072	phosphodiesterase 4B, cAMP-specific (phosphodiesterase E4 dunce homolog, Drosophila)
FLJ11286	218429_s_at	3.203	NM_018381	Hs.175120	hypothetical protein FLJ11286
PILRA	222218_s_at	3.2	AJ400843	Hs.444407	paired immunoglobin-like type 2 receptor alpha
MAF	209348_s_at	3.178	AF055376	Hs.134859	v-maf musculoaponeurotic fibrosarcoma oncogene homolog (avian)
STC2	203439_s_at	3.177	BC000658	Hs.233160	stanniocalcin 2
COL6A1	213428_s_at	3.155	AA292373	Hs.474053	collagen, type VI, alpha 1
HIP1	205425_at	3.142	NM_005338	Hs.329266	huntingtin interacting protein 1
TCF4	212386_at	3.142	BF592782	Hs.200285	Transcription factor 4
ACSL1	201963_at	3.136	NM_021122	Hs.406678	acyl-CoA synthetase long-chain family member 1
SP100	202864_s_at	3.131	NM_003113	Hs.369056	nuclear antigen Sp100
CTSB	200839_s_at	3.13	NM_001908	Hs.520898	cathepsin B
NFE2L1	200758_s_at	3.127	AI361227	Hs.514284	nuclear factor (erythroid-derived 2)-like 1

VEGF	210512_s_at	3.125	AF022375	Hs.73793	vascular endothelial growth factor
RHBDL6	219202_at	3.124	NM_024599	Hs.464157	rhomboid, veinlet-like 6 (Drosophila)
LR8	220532_s_at	3.121	NM_014020	Hs.521295	LR8 protein
ICAM1	215485_s_at	3.121	AA284705	Hs.515126	intercellular adhesion molecule 1 (CD54), human rhinovirus receptor
MMP14	202828_s_at	3.12	NM_004995	Hs.2399	matrix metallopeptidase 14 (membrane-inserted)
TCF4	203753_at	3.11	NM_003199	Hs.200285	transcription factor 4
RIN2	209684_at	3.092	AL136924	Hs.472270	Ras and Rab interactor 2
PHLDA1	217997_at	3.089	AI795908	Hs.558462	pleckstrin homology-like domain, family A, member 1
BST2	201641_at	3.074	NM_004335	Hs.118110	bone marrow stromal cell antigen 2
CCR1	205099_s_at	3.056	NM_001295	Hs.301921	chemokine (C-C motif) receptor 1
MUC1	207847_s_at	3.056	NM_002456	Hs.89603	mucin 1, transmembrane
TNFAIP2	202510_s_at	3.056	NM_006291	Hs.525607	tumor necrosis factor, alpha-induced protein 2
HLA-C	211799_x_at	3.047	U62824	Hs.534125	major histocompatibility complex, class I, C
RAB8B	219210_s_at	3.042	NM_016530	Hs.389733	RAB8B, member RAS oncogene family
CYBB	203923_s_at	3.037	NM_000397	Hs.292356	cytochrome b-245, beta polypeptide (chronic granulomatous disease)
SQSTM1	201471_s_at	3.013	NM_003900	Hs.529892	sequestosome 1
AMPD3	207992_s_at	3.008	NM_000480	Hs.501890	adenosine monophosphate deaminase (isoform E)
FAM26B	57715_at	3.008	W72694	Hs.241545	family with sequence similarity 26, member B
HLA-G ; HLA-H	217436_x_at	2.989	M80469	Hs.512152	HLA-G histocompatibility antigen, class I, G ; major histocompatibility complex, class I, H (pseudogene)
IFNGR2	201642_at	2.986	NM_005534	Hs.517240	interferon gamma receptor 2 (interferon gamma transducer 1)
IFI30	201422_at	2.982	NM_006332	Hs.14623	interferon, gamma-inducible protein 30
PHF11	221816_s_at	2.975	BF055474	Hs.369039	PHD finger protein 11
EIF2AK2	204211_x_at	2.97	NM_002759	Hs.131431	eukaryotic translation initiation factor 2-alpha kinase 2
ISGF3G	203882_at	2.964	NM_006084	Hs.1706	interferon-stimulated transcription factor 3, gamma 48kDa
N4BP1	32069_at	2.942	AB014515	Hs.558438	Nedd4 binding protein 1
GRB10	209409_at	2.938	D86962	Hs.164060	growth factor receptor-bound protein 10
STAT2	205170_at	2.917	NM_005419	Hs.530595	signal transducer and activator of transcription 2, 113kDa
NINJ1	203045_at	2.906	NM_004148	Hs.494457	ninjurin 1
FTHP1	211628_x_at	2.905	J04755	Hs.453583	ferritin, heavy polypeptide pseudogene 1 ; ferritin, heavy polypeptide pseudogene 1
NRP1	212298_at	2.881	BE620457	Hs.131704	neuropilin 1
ETV5	203349_s_at	2.872	NM_004454	Hs.43697	ets variant gene 5 (ets-related molecule)
MT2A	212185_x_at	2.872	NM_005953	Hs.418241	metallothionein 2A
BHLHB2	201170_s_at	2.87	NM_003670	Hs.171825	basic helix-loop-helix domain containing, class B, 2
NFE2L1	200759_x_at	2.869	NM_003204	Hs.514284	nuclear factor (erythroid-derived 2)-like 1
RNF41	201961_s_at	2.864	AL583171	Hs.524502	ring finger protein 41
MARCKS	213002_at	2.858	AA770596	Hs.519909	Myristoylated alanine-rich protein kinase C substrate
APOBEC3G	204205_at	2.857	NM_021822	Hs.474853	apolipoprotein B mRNA editing enzyme, catalytic polypeptide-like 3G
H1F0	208886_at	2.851	BC000145	Hs.226117	H1 histone family, member 0
ITGAL	213475_s_at	2.848	AC002310	Hs.174103	integrin, alpha L (antigen CD11A (p180), lymphocyte function-associated antigen 1; alpha polypeptide)
CD44	209835_x_at	2.844	BC004372	Hs.502328	CD44 antigen (homing function and Indian blood group system)
TOM1	202807_s_at	2.842	NM_005488	Hs.474705	target of myb1 (chicken)
OPTN	202073_at	2.832	AV757675	Hs.332706	optineurin

PPARD	208044_s_at	2.829	NM_006238	Hs.485196	peroxisome proliferative activated receptor, delta
MERTK	211913_s_at	2.825	L08961	Hs.306178	c-mer proto-oncogene tyrosine kinase ; c-mer proto-oncogene tyrosine kinase
TCTEL1	201999_s_at	2.808	NM_006519	Hs.445999	t-complex-associated-testis-expressed 1-like 1
MMP12	204580_at	2.807	NM_002426	Hs.1695	matrix metallopeptidase 12 (macrophage elastase)
CHMP5	219356_s_at	2.797	NM_016410	Hs.415534	chromatin modifying protein 5
LAP3	217933_s_at	2.796	NM_015907	Hs.479264	leucine aminopeptidase 3
CFLAR	209939_x_at	2.791	AF005775	Hs.390736	CASP8 and FADD-like apoptosis regulator
AXL	202686_s_at	2.757	NM_021913	Hs.466791	AXL receptor tyrosine kinase
SEMA4A	219259_at	2.752	NM_022367	Hs.408846	sema domain, immunoglobulin domain (Ig), transmembrane domain (TM) and short cytoplasmic domain, (semaphorin) 4A
LGALS3BP	200923_at	2.743	NM_005567	Hs.514535	lectin, galactoside-binding, soluble, 3 binding protein
DPY19L1	212792_at	2.733	AB020684	Hs.408623	dpy-19-like 1 (C. elegans)
IL23A	220054_at	2.726	NM_016584	Hs.98309	interleukin 23, alpha subunit p19
PSD3	203355_s_at	2.722	NM_015310	Hs.434255	pleckstrin and Sec7 domain containing 3
CD44	204490_s_at	2.716	M24915	Hs.502328	CD44 antigen (homing function and Indian blood group system)
ASNS	205047_s_at	2.71	NM_001673	Hs.489207	asparagine synthetase
TOR1B	209593_s_at	2.706	AF317129	Hs.252682	torsin family 1, member B (torsin B)
NFE2L1	214179_s_at	2.701	H93013	Hs.514284	nuclear factor (erythroid-derived 2)-like 1
GK	207387_s_at	2.7	NM_000167	Hs.1466	glycerol kinase
GAS7	202191_s_at	2.696	BE439987	Hs.462214	growth arrest-specific 7
IFITM2	201315_x_at	2.681	NM_006435	Hs.174195	interferon induced transmembrane protein 2 (1-8D)
CYP1B1	202437_s_at	2.675	NM_000104	Hs.154654	cytochrome P450, family 1, subfamily B, polypeptide 1
CYLD	221903_s_at	2.67	BE046443	Hs.432993	cylindromatosis (turban tumor syndrome)
FLJ38348	213294_at	2.633	AV755522	Hs.546523	Hypothetical protein FLJ38348
pp9099	204436_at	2.629	NM_025201	Hs.458575	PH domain-containing protein
PLCL1	205934_at	2.628	NM_006226	Hs.153322	phospholipase C-like 1
CASP1	211368_s_at	2.628	U13700	Hs.2490	caspase 1, apoptosis-related cysteine peptidase (interleukin 1, beta, convertase)
C1orf38	207571_x_at	2.626	NM_004848	Hs.10649	chromosome 1 open reading frame 38
S100A9	203535_at	2.625	NM_002965	Hs.112405	S100 calcium binding protein A9 (calgranulin B)
PLXNC1	206471_s_at	2.623	NM_005761	Hs.554795	plexin C1
ARID5B	212614_at	2.623	BG285011	Hs.535297	AT rich interactive domain 5B (MRF1-like)
LIMK2	217475_s_at	2.622	AC002073	Hs.474596	LIM domain kinase 2
SLC2A3	202499_s_at	2.618	NM_006931	Hs.419240	solute carrier family 2 (facilitated glucose transporter), member 3
ETS2	201329_s_at	2.617	NM_005239	Hs.517296	v-ets erythroblastosis virus E26 oncogene homolog 2 (avian)
CTSB	213274_s_at	2.615	AA020826	Hs.520898	cathepsin B
KLF9	203543_s_at	2.605	NM_001206	Hs.150557	Kruppel-like factor 9
C1orf24	217966_s_at	2.605	NM_022083	Hs.518662	chromosome 1 open reading frame 24
PNMA2	209598_at	2.603	AB020690	Hs.521466	paraneoplastic antigen MA2
HLA-G	211528_x_at	2.599	M90685	Hs.512152	HLA-G histocompatibility antigen, class I, G
FKBP5	204560_at	2.594	NM_004117	Hs.558328	FK506 binding protein 5
LGALS9	203236_s_at	2.593	NM_009587	Hs.81337	lectin, galactoside-binding, soluble, 9 (galectin 9)
C1orf38	210785_s_at	2.585	AB035482	Hs.10649	chromosome 1 open reading frame 38
SLC15A3	219593_at	2.581	NM_016582	Hs.237856	solute carrier family 15, member 3

VEGF	210513_s_at	2.573	AF091352	Hs.73793	vascular endothelial growth factor
MYCL1	214058_at	2.568	M19720	Hs.437922	v-myc myelocytomatosis viral oncogene homolog 1, lung carcinoma derived (avian)
NAGK	218231_at	2.566	NM_017567	Hs.7036	N-acetylglucosamine kinase ; N-acetylglucosamine kinase
TCF4	213891_s_at	2.565	AI927067	Hs.200285	Transcription factor 4
NQO1	210519_s_at	2.564	BC000906	Hs.406515	NAD(P)H dehydrogenase, quinone 1
DTX4	212611_at	2.562	AV728526	Hs.558464	deltex 4 homolog (Drosophila)
CTSB	200838_at	2.557	NM_001908	Hs.520898	cathepsin B
KIAA0674	76897_s_at	2.554	AA628140	Hs.522351	KIAA0674
EXT1	201995_at	2.536	NM_000127	Hs.492618	exostoses (multiple) 1
UBE1L	203281_s_at	2.535	NM_003335	Hs.16695	ubiquitin-activating enzyme E1-like
WARS	200628_s_at	2.532	M61715	Hs.497599	tryptophanyl-tRNA synthetase
MARCKSL1	200644_at	2.53	NM_023009	Hs.75061	MARCKS-like 1
CD44	212014_x_at	2.528	AI493245	Hs.502328	CD44 antigen (homing function and Indian blood group system)
EHD1	208112_x_at	2.524	NM_006795	Hs.523774	EH-domain containing 1
EHD1	209039_x_at	2.523	AF001434	Hs.523774	EH-domain containing 1
ARTS-1	210385_s_at	2.523	AF106037	Hs.436186	type 1 tumor necrosis factor receptor shedding aminopeptidase regulator
PPARD	37152_at	2.521	L07592	Hs.485196	peroxisome proliferative activated receptor, delta
CFLAR	210563_x_at	2.51	U97075	Hs.390736	CASP8 and FADD-like apoptosis regulator
RAFTLIN	212646_at	2.506	D42043	Hs.98910	raft-linking protein
PTPRC	212587_s_at	2.506	AI809341	Hs.192039	protein tyrosine phosphatase, receptor type, C
UGCG	221765_at	2.501	AI378044	Hs.304249	UDP-glucose ceramide glucosyltransferase
NADK	208917_x_at	2.496	BE674658	Hs.456572	NAD kinase
PSAT1	220892_s_at	2.49	NM_021154	Hs.494261	phosphoserine aminotransferase 1
SECTM1	213716_s_at	2.486	BF939675	Hs.558009	secreted and transmembrane 1
LRP12	219631_at	2.481	NM_024937	Hs.301974	low density lipoprotein-related protein 12
RAB13	202252_at	2.478	NM_002870	Hs.558375	RAB13, member RAS oncogene family
CTNND1	208862_s_at	2.478	AW073672	Hs.166011	catenin (cadherin-associated protein), delta 1
CHMP5	218085_at	2.473	NM_015961	Hs.415534	chromatin modifying protein 5
SLA	203761_at	2.469	NM_006748	Hs.75367	Src-like-adaptor ; Src-like-adaptor
ABCA1	203505_at	2.468	AF285167	Hs.429294	ATP-binding cassette, sub-family A (ABC1), member 1
HIST2H2AA	218280_x_at	2.465	NM_003516	Hs.530461	histone 2, H2aa
LAIR2	207509_s_at	2.459	NM_002288	Hs.43803	leukocyte-associated Ig-like receptor 2
PHLDA1	217996_at	2.454	AA576961	Hs.558462	pleckstrin homology-like domain, family A, member 1
PMAIP1	204285_s_at	2.451	AI857639	Hs.96	phorbol-12-myristate-13-acetate-induced protein 1
TRIM38	203610_s_at	2.45	AI363270	Hs.202510	tripartite motif-containing 38
HECA	218603_at	2.449	NM_016217	Hs.197644	headcase homolog (Drosophila)
PLXNC1	213241_at	2.449	AF035307	Hs.554795	plexin C1
RIT1	209882_at	2.443	AF084462	Hs.491234	Ras-like without CAAX 1
WSB1	201295_s_at	2.44	BF111821	Hs.446017	WD repeat and SOCS box-containing 1
TCF4	212382_at	2.438	BF433429	Hs.200285	Transcription factor 4
PIR	207469_s_at	2.435	NM_003662	Hs.495728	pirin (iron-binding nuclear protein)
PVRL3	213325_at	2.435	AA129716	Hs.293917	poliovirus receptor-related 3

NCOA2	205732_s_at	2.431	NM_006540	Hs.446678	nuclear receptor coactivator 2
RPS6KA2	212912_at	2.431	AI992251	Hs.147119	ribosomal protein S6 kinase, 90kDa, polypeptide 2
CFLAR	208485_x_at	2.423	NM_003879	Hs.390736	CASP8 and FADD-like apoptosis regulator
HLA-A	215313_x_at	2.423	AA573862	Hs.181244	major histocompatibility complex, class I, A
CASP1	211367_s_at	2.418	U13699	Hs.2490	caspase 1, apoptosis-related cysteine peptidase (interleukin 1, beta, convertase)
PTPNS1	202897_at	2.411	AB023430	Hs.128846	protein tyrosine phosphatase, non-receptor type substrate 1
ASS	207076_s_at	2.41	NM_000050	Hs.558301	argininosuccinate synthetase
FYB	205285_s_at	2.405	AI633888	Hs.370503	FYN binding protein (FYB-120/130)
TNIP1	207196_s_at	2.4	NM_006058	Hs.355141	TNFAIP3 interacting protein 1
FOS	209189_at	2.398	BC004490	Hs.25647	v-fos FBJ murine osteosarcoma viral oncogene homolog
HLA-E	200905_x_at	2.396	NM_005516	Hs.381008	major histocompatibility complex, class I, E
RGS16	209325_s_at	2.395	U94829	Hs.413297	regulator of G-protein signalling 16
LILRB1	207104_x_at	2.391	NM_006669	Hs.149924	leukocyte immunoglobulin-like receptor, subfamily B (with TM and ITIM domains), member 1
HLX1	214438_at	2.39	M60721	Hs.74870	H2.0-like homeo box 1 (Drosophila)
AGRN	212285_s_at	2.382	AW008051	Hs.273330	agrin
PANX1	204715_at	2.379	NM_015368	Hs.503584	pannexin 1
PSTPIP2	219938_s_at	2.377	NM_024430	Hs.368623	proline-serine-threonine phosphatase interacting protein 2
SDS	205695_at	2.377	NM_006843	Hs.439023	serine dehydratase
PTPRO	208121_s_at	2.376	NM_002848	Hs.160871	protein tyrosine phosphatase, receptor type, O ; protein tyrosine phosphatase, receptor type, O
CEBPB	212501_at	2.376	AL564683	Hs.517106	CCAAT/enhancer binding protein (C/EBP), beta
PTPRC	212588_at	2.372	Y00062	Hs.192039	protein tyrosine phosphatase, receptor type, C
CYP1B1	202436_s_at	2.371	AU144855	Hs.154654	cytochrome P450, family 1, subfamily B, polypeptide 1
MYD88	209124_at	2.368	U70451	Hs.82116	myeloid differentiation primary response gene (88)
GRINA	212090_at	2.364	AL571424	Hs.339697	glutamate receptor, ionotropic, N-methyl D-asparate-associated protein 1 (glutamate binding)
GSDMDC1	218154_at	2.361	NM_024736	Hs.118983	gasdermin domain containing 1
NADK	215159_s_at	2.358	AI239732	Hs.456572	NAD kinase
IRF2	203275_at	2.358	NM_002199	Hs.374097	interferon regulatory factor 2
SASH1	213236_at	2.357	AK025495	Hs.193133	SAM and SH3 domain containing 1
ATF4	200779_at	2.353	NM_001675	Hs.496487	activating transcription factor 4 (tax-responsive enhancer element B67)
KIAA1280	219520_s_at	2.348	NM_018458	Hs.527524	KIAA1280 protein
SQRDL	217995_at	2.339	NM_021199	Hs.511251	sulfide quinone reductase-like (yeast)
SLC31A2	204204_at	2.33	NM_001860	Hs.24030	solute carrier family 31 (copper transporters), member 2
ST8SIA4	206925_at	2.328	NM_005668	Hs.308628	ST8 alpha-N-acetyl-neuraminide alpha-2,8-sialyltransferase 4
NFE2L3	204702_s_at	2.326	NM_004289	Hs.404741	nuclear factor (erythroid-derived 2)-like 3
LIMK2	210582_s_at	2.325	AL117466	Hs.474596	LIM domain kinase 2
CASP1	209970_x_at	2.323	M87507	Hs.2490	caspase 1, apoptosis-related cysteine peptidase (interleukin 1, beta, convertase)
NEU1	208926_at	2.315	U84246	Hs.520037	sialidase 1 (lysosomal sialidase)
CCR1	205098_at	2.31	AI421071	Hs.301921	chemokine (C-C motif) receptor 1
SLC7A7	204588_s_at	2.305	NM_003982	Hs.513147	solute carrier family 7 (cationic amino acid transporter, y+ system), member 7
PPP1R15A	37028_at	2.305	U83981	Hs.76556	protein phosphatase 1, regulatory (inhibitor) subunit 15A
HLA-F	204806_x_at	2.305	NM_018950	Hs.519972	major histocompatibility complex, class I, F
CBR1	209213_at	2.299	BC002511	Hs.88778	carbonyl reductase 1

ATF3	202672_s_at	2.297	NM_001674	Hs.460	activating transcription factor 3
SP100	210218_s_at	2.291	U36501	Hs.369056	nuclear antigen Sp100
GIMAP5	64064_at	2.29	AI435089	Hs.412331	GTPase, IMAP family member 5
FER1L3	201798_s_at	2.286	NM_013451	Hs.500572	fer-1-like 3, myoferlin (C. elegans)
NPC1	202679_at	2.277	NM_000271	Hs.464779	Niemann-Pick disease, type C1
GRB10	210999_s_at	2.276	U66065	Hs.164060	growth factor receptor-bound protein 10
HLA-F	221875_x_at	2.271	AW514210	Hs.519972	major histocompatibility complex, class I, F
SLA	203760_s_at	2.27	U44403	Hs.75367	Src-like-adaptor ; Src-like-adaptor
PROCR	203650_at	2.264	NM_006404	Hs.82353	protein C receptor, endothelial (EPCR)
CD36	209555_s_at	2.263	M98399	Hs.120949	CD36 antigen (collagen type I receptor, thrombospondin receptor)
CENTA2	219358_s_at	2.263	NM_018404	Hs.514063	centaurin, alpha 2
SPTLC2	216202_s_at	2.263	U15555	Hs.435661	serine palmitoyltransferase, long chain base subunit 2
PTPRC	207238_s_at	2.263	NM_002838	Hs.192039	protein tyrosine phosphatase, receptor type, C
P8	209230_s_at	2.261	AF135266	Hs.513463	p8 protein (candidate of metastasis 1)
SMAD7	204790_at	2.26	NM_005904	Hs.465087	SMAD, mothers against DPP homolog 7 (Drosophila)
MT1X	208581_x_at	2.259	NM_005952	Hs.374950	metallothionein 1X
RTN1	203485_at	2.259	NM_021136	Hs.368626	reticulon 1
UGCG	204881_s_at	2.256	NM_003358	Hs.304249	UDP-glucose ceramide glucosyltransferase
OSBPL11	218304_s_at	2.254	NM_022776	Hs.477440	oxysterol binding protein-like 11
MMP14	160020_at	2.254	Z48481	Hs.2399	matrix metallopeptidase 14 (membrane-inserted)
AGRN	217419_x_at	2.252	AK021586	Hs.273330	agrin
MGLL	211026_s_at	2.246	BC006230	Hs.277035	monoglyceride lipase ; monoglyceride lipase
CFLAR	211317_s_at	2.245	AF041461	Hs.390736	CASP8 and FADD-like apoptosis regulator
SSH1	221753_at	2.24	AI651213	Hs.199763	slingshot homolog 1 (Drosophila)
ABCA1	203504_s_at	2.239	NM_005502	Hs.429294	ATP-binding cassette, sub-family A (ABC1), member 1
CYP1B1	202435_s_at	2.238	AU154504	Hs.154654	cytochrome P450, family 1, subfamily B, polypeptide 1
HLA-B	208729_x_at	2.237	D83043	Hs.77961	major histocompatibility complex, class I, B
NFKB1	209239_at	2.236	M55643	Hs.431926	nuclear factor of kappa light polypeptide gene enhancer in B-cells 1 (p105)
CSPG2	215646_s_at	2.235	R94644	Hs.443681	chondroitin sulfate proteoglycan 2 (versican) ; chondroitin sulfate proteoglycan 2 (versican)
ZCCHC2	219062_s_at	2.235	NM_017742	Hs.114191	zinc finger, CCHC domain containing 2
KIAA0247	202181_at	2.233	NM_014734	Hs.440025	KIAA0247
CSF2RB	205159_at	2.229	AV756141	Hs.285401	colony stimulating factor 2 receptor, beta, low-affinity (granulocyte-macrophage)
SLC11A2	203124_s_at	2.227	NM_000617	Hs.505545	solute carrier family 11 (proton-coupled divalent metal ion transporters), member 2
CASP1	211366_x_at	2.221	U13698	Hs.2490	caspase 1, apoptosis-related cysteine peptidase (interleukin 1, beta, convertase)
CARS	212971_at	2.219	AI769685	Hs.274873	cysteinyl-tRNA synthetase
STAT4	206118_at	2.216	NM_003151	Hs.80642	signal transducer and activator of transcription 4
LIMK2	202193_at	2.215	NM_005569	Hs.474596	LIM domain kinase 2
CASP7	207181_s_at	2.214	NM_001227	Hs.9216	caspase 7, apoptosis-related cysteine peptidase
P2RX4	204088_at	2.212	NM_002560	Hs.321709	purinergic receptor P2X, ligand-gated ion channel, 4
CSPG2	211571_s_at	2.206	D32039	Hs.443681	chondroitin sulfate proteoglycan 2 (versican)
STAT3	208991_at	2.206	AA634272	Hs.463059	signal transducer and activator of transcription 3 (acute-phase response factor)
SLC7A5	201195_s_at	2.202	AB018009	Hs.513797	solute carrier family 7 (cationic amino acid transporter, y+ system), member 5

SLC7A11	209921_at	2.202	AB040875	Hs.390594	solute carrier family 7, (cationic amino acid transporter, y+ system) member 11
HLA-G	211529_x_at	2.201	M90684	Hs.512152	HLA-G histocompatibility antigen, class I, G
PVRL2	203149_at	2.194	NM_002856	Hs.326371	poliovirus receptor-related 2 (herpesvirus entry mediator B)
CHST11	219634_at	2.192	NM_018413	Hs.17569	carbohydrate (chondroitin 4) sulfotransferase 11
TRIM16	204341_at	2.172	NM_006470	Hs.164324	tripartite motif-containing 16 ; similar to tripartite motif-containing 16; estrogen-responsive B box protein
GK	215977_x_at	2.171	X68285	Hs.1466	glycerol kinase
RASSF4	49306_at	2.169	AI890191	Hs.522895	Ras association (RalGDS/AF-6) domain family 4
IKBKE	204549_at	2.167	NM_014002	Hs.321045	inhibitor of kappa light polypeptide gene enhancer in B-cells, kinase epsilon
MVP	202180_s_at	2.165	NM_017458	Hs.513488	major vault protein
PTX3	206157_at	2.164	NM_002852	Hs.127657	pentraxin-related gene, rapidly induced by IL-1 beta
IFNGR1	202727_s_at	2.161	NM_000416	Hs.520414	interferon gamma receptor 1
GK	217167_x_at	2.159	AJ252550	Hs.1466	glycerol kinase
TCF7L2	216037_x_at	2.159	AA664011	Hs.501080	transcription factor 7-like 2 (T-cell specific, HMG-box)
NQO1	201468_s_at	2.159	NM_000903	Hs.406515	NAD(P)H dehydrogenase, quinone 1
DENND3	212974_at	2.153	AI808958	Hs.18166	DENN/MADD domain containing 3
CD53	203416_at	2.151	NM_000560	Hs.443057	CD53 antigen
NR1H3	203920_at	2.15	NM_005693	Hs.438863	nuclear receptor subfamily 1, group H, member 3
JUNB	201473_at	2.149	NM_002229	Hs.25292	jun B proto-oncogene
IKBKE	214398_s_at	2.147	AW340333	Hs.321045	inhibitor of kappa light polypeptide gene enhancer in B-cells, kinase epsilon
PPA1	217848_s_at	2.146	NM_021129	Hs.437403	pyrophosphatase (inorganic) 1
NPTX2	213479_at	2.137	U26662	Hs.3281	neuronal pentraxin II
FYB	211795_s_at	2.134	AF198052	Hs.370503	FYN binding protein (FYB-120/130)
NFKBIE	203927_at	2.132	NM_004556	Hs.458276	nuclear factor of kappa light polypeptide gene enhancer in B-cells inhibitor, epsilon
RGS16	209324_s_at	2.131	BF304996	Hs.413297	regulator of G-protein signalling 16
PLEK	203470_s_at	2.128	AI433595	Hs.468840	pleckstrin
RAB6IP1	212561_at	2.124	AA349595	Hs.501857	RAB6 interacting protein 1
ADA	204639_at	2.123	NM_000022	Hs.407135	adenosine deaminase
RHOQ	212117_at	2.119	BF978689	Hs.549125	ras homolog gene family, member Q
EMR1	207111_at	2.119	NM_001974	Hs.2375	egf-like module containing, mucin-like, hormone receptor-like 1
PLAUR	210845_s_at	2.118	U08839	Hs.466871	plasminogen activator, urokinase receptor
PTPNS1	202895_s_at	2.117	D86043	Hs.128846	protein tyrosine phosphatase, non-receptor type substrate 1
G6PD	202275_at	2.116	NM_000402	Hs.461047	glucose-6-phosphate dehydrogenase
FCGR1A	214511_x_at	2.115	L03419	Hs.534956	Fc fragment of IgG, high affinity Ia, receptor (CD64) ; Fc-gamma receptor I B2
SERPINI1	205352_at	2.11	NM_005025	Hs.478153	serpin peptidase inhibitor, clade I (neuroserpin), member 1
SASH1	41644_at	2.108	AB018333	Hs.193133	SAM and SH3 domain containing 1
IER5	218611_at	2.108	NM_016545	Hs.15725	immediate early response 5
LRP12	220253_s_at	2.106	NM_013437	Hs.301974	low density lipoprotein-related protein 12
PRKCA	215195_at	2.101	AF035594	Hs.531704	protein kinase C, alpha
MERTK	206028_s_at	2.101	NM_006343	Hs.306178	c-mer proto-oncogene tyrosine kinase
ATP10A	214255_at	2.096	N35112	Hs.128041	ATPase, Class V, type 10A
CFLAR	211862_x_at	2.091	AF015451	Hs.390736	CASP8 and FADD-like apoptosis regulator
KCNN4	204401_at	2.09	NM_002250	Hs.10082	potassium intermediate/small conductance calcium-activated channel, subfamily N, member 4

ADAR	201786_s_at	2.088	NM_001111	Hs.12341	adenosine deaminase, RNA-specific
NRN1	218625_at	2.086	NM_016588	Hs.103291	neuritin 1
IRF1	202531_at	2.084	NM_002198	Hs.436061	interferon regulatory factor 1
RASSF4	221578_at	2.081	AF260335	Hs.522895	Ras association (RalGDS/AF-6) domain family 4
PCK2	202847_at	2.081	NM_004563	Hs.75812	phosphoenolpyruvate carboxykinase 2 (mitochondrial)
SH3BP5	201811_x_at	2.076	NM_004844	Hs.257761	SH3-domain binding protein 5 (BTK-associated)
PSAP	200871_s_at	2.068	NM_002778	Hs.523004	prosaposin (variant Gaucher disease and variant metachromatic leukodystrophy)
CTSO	203758_at	2.066	AV729484	Hs.75262	cathepsin O
CD40	35150_at	2.066	X60592	Hs.472860	CD40 antigen (TNF receptor superfamily member 5)
TCF7L2	216511_s_at	2.063	AJ270770	Hs.501080	transcription factor 7-like 2 (T-cell specific, HMG-box)
FMNL1	204789_at	2.063	NM_005892	Hs.100217	formin-like 1
PILRA	219788_at	2.062	NM_013439	Hs.444407	paired immunoglobin-like type 2 receptor alpha
ECOP	208091_s_at	2.052	NM_030796	Hs.488307	EGFR-coamplified and overexpressed protein ; EGFR-coamplified and overexpressed protein
HIST2H2BE	202708_s_at	2.048	NM_003528	Hs.2178	histone 2, H2be
ANXA3	209369_at	2.043	M63310	Hs.480042	annexin A3
GAS7	202192_s_at	2.04	NM_005890	Hs.462214	growth arrest-specific 7
STK10	203047_at	2.04	NM_005990	Hs.519756	serine/threonine kinase 10
ENG	201809_s_at	2.039	NM_000118	Hs.76753	endoglin (Osler-Rendu-Weber syndrome 1)
RAB31	217763_s_at	2.037	NM_006868	Hs.99528	RAB31, member RAS oncogene family
CLEC4M	207995_s_at	2.033	NM_014257	Hs.421437	C-type lectin domain family 4, member M
HLA-B	211911_x_at	2.031	L07950	Hs.77961	major histocompatibility complex, class I, B ; major histocompatibility complex, class I, B
PLEK	203471_s_at	2.029	NM_002664	Hs.468840	pleckstrin
TAPBP	208829_at	2.028	AF029750	Hs.370937	TAP binding protein (tapasin)
TRAF3IP3	213888_s_at	2.027	AL022398	Hs.147434	TRAF3 interacting protein 3
TAP2	204769_s_at	2.023	M74447	Hs.502	transporter 2, ATP-binding cassette, sub-family B (MDR/TAP)
FOXJ2	203734_at	2.022	NM_018416	Hs.120844	forkhead box J2
PHACTR4	219235_s_at	2.021	NM_023923	Hs.225641	phosphatase and actin regulator 4
CTAGE1	220957_at	2.017	NM_022663	Hs.406709	cutaneous T-cell lymphoma-associated antigen 1
HES1	203394_s_at	2.013	BE973687	Hs.250666	hairy and enhancer of split 1, (Drosophila)
TANK	207616_s_at	2.006	NM_004180	Hs.132257	TRAF family member-associated NFKB activator
FXN	205565_s_at	0.5	NM_000144	Hs.29978	frataxin
TRIOBP	210276_s_at	0.5	AF281030	Hs.533030	TRIO and F-actin binding protein
ACTN1	208636_at	0.499	AI082078	Hs.509765	Actinin, alpha 1
SHFM1	202276_at	0.499	NM_006304	Hs.489201	split hand/foot malformation (ectrodactyly) type 1
PGLS	218388_at	0.499	NM_012088	Hs.466165	6-phosphogluconolactonase
FLJ10154	218067_s_at	0.499	NM_018011	Hs.508644	hypothetical protein FLJ10154
LOC339448	222000_at	0.498	AI915947	Hs.103939	hypothetical protein LOC339448
GGA2	208914_at	0.498	BE646414	Hs.460336	golgi associated, gamma adaptin ear containing, ARF binding protein 2
HEBP2	203430_at	0.497	NM_014320	Hs.486589	heme binding protein 2
NIPBL	212483_at	0.497	AW339587	Hs.481927	Nipped-B homolog (Drosophila)
FUBP1	214093_s_at	0.496	AA156865	Hs.567255	far upstream element (FUSE) binding protein 1
HSU79274	204521_at	0.496	NM_013300	Hs.436618	protein predicted by clone 23733

PROS1	207808_s_at	0.495	NM_000313	Hs.64016	protein S (alpha)
MAGOH	210093_s_at	0.495	AF067173	Hs.421576	mago-nashi homolog, proliferation-associated (Drosophila)
FAM20B	202916_s_at	0.495	NM_014864	Hs.5737	family with sequence similarity 20, member B
ZF	202978_s_at	0.495	AW204564	Hs.535319	HCF-binding transcription factor Zhangfei
C1orf41	215691_x_at	0.494	AV702994	Hs.525462	chromosome 1 open reading frame 41
LOC93081	213346_at	0.494	BE748563	Hs.398111	hypothetical protein BC015148
C9orf40	218904_s_at	0.494	NM_017998	Hs.532296	chromosome 9 open reading frame 40
AKAP1	210625_s_at	0.494	U34074	Hs.463506	A kinase (PRKA) anchor protein 1
ARHGEF6	209539_at	0.493	D25304	Hs.522795	Rac/Cdc42 guanine nucleotide exchange factor (GEF) 6
C16orf33	218493_at	0.493	NM_024571	Hs.15277	chromosome 16 open reading frame 33
KIAA0960	213894_at	0.492	BF447246	Hs.120855	KIAA0960 protein
OPHN1	206323_x_at	0.492	NM_002547	Hs.128824	oligophrenin 1
TIMP3	201149_s_at	0.491	U67195	Hs.297324	TIMP metallopeptidase inhibitor 3 (Sorsby fundus dystrophy, pseudoinflammatory)
NAP1L1	204528_s_at	0.491	NM_004537	Hs.524599	nucleosome assembly protein 1-like 1
TSC2	215735_s_at	0.49	AC005600	Hs.90303	tuberous sclerosis 2
TAF4	213090_s_at	0.49	AI744029	Hs.473243	TAF4 RNA polymerase II, TATA box binding protein (TBP)-associated factor, 135kDa
LARS	217810_x_at	0.49	NM_020117	Hs.432674	leucyl-tRNA synthetase
RNF170	220985_s_at	0.49	NM_030954	Hs.491626	ring finger protein 170 ; ring finger protein 170
TTK	204822_at	0.489	NM_003318	Hs.169840	TTK protein kinase
SYK	209269_s_at	0.489	AW450910	Hs.371720	Spleen tyrosine kinase
SEPT6 ; N-PAC	212414_s_at	0.489	D50918	Hs.387255	septin 6 ; cytokine-like nuclear factor n-pac
C2orf17	221984_s_at	0.488	AL040896	Hs.516707	chromosome 2 open reading frame 17
C19orf2	222266_at	0.488	BF796940	Hs.466391	Chromosome 19 open reading frame 2
TRAPPC6A	204985_s_at	0.488	NM_024108	Hs.466929	trafficking protein particle complex 6A
RPL22	221726_at	0.487	BE250348	Hs.515329	ribosomal protein L22
WDR39	217501_at	0.487	AI339732	Hs.12109	WD repeat domain 39
TERF1	203449_s_at	0.487	NM_017489	Hs.442707	telomeric repeat binding factor (NIMA-interacting) 1
TOP2A	201291_s_at	0.487	AU159942	Hs.156346	topoisomerase (DNA) II alpha 170kDa
CLGN	205830_at	0.486	NM_004362	Hs.86368	calmegin
TTC3	208073_x_at	0.486	NM_003316	Hs.368214	tetratricopeptide repeat domain 3
DKC1	201478_s_at	0.485	U59151	Hs.4747	dyskeratosis congenita 1, dyskerin
NRGN	204081_at	0.485	NM_006176	Hs.524116	neurogranin (protein kinase C substrate, RC3)
FBXO5	218875_s_at	0.484	NM_012177	Hs.520506	F-box protein 5
WHSC1	209053_s_at	0.484	BE793789	Hs.113876	Wolf-Hirschhorn syndrome candidate 1
ITGB1BP1	203336_s_at	0.484	AL548363	Hs.467662	integrin beta 1 binding protein 1
PFAAP5	221899_at	0.483	AI809961	Hs.507680	Hypothetical gene CG012
C13orf18	219471_at	0.483	NM_025113	Hs.98117	chromosome 13 open reading frame 18
FLI1	204236_at	0.483	NM_002017	Hs.504281	Friend leukemia virus integration 1
MSH6	211450_s_at	0.483	D89646	Hs.445052	mutS homolog 6 (E. coli)
POLR1D	218258_at	0.482	NM_015972		polymerase (RNA) I polypeptide D, 16kDa
PRSS12	205515_at	0.482	NM_003619	Hs.445857	protease, serine, 12 (neurotrypsin, motopsin)
	209478_at	0.482	U95006		

LOC130074	212017_at	0.482	BF677404	Hs.534679	hypothetical protein LOC130074
CRI1	208669_s_at	0.481	AF109873	Hs.255973	CREBBP/EP300 inhibitor 1
MGC16943	213365_at	0.481	N64622	Hs.248437	similar to RIKEN cDNA 4933424N09 gene
ATP2B4	212136_at	0.481	AW517686	Hs.343522	ATPase, Ca++ transporting, plasma membrane 4
DHFR	48808_at	0.481	AI144299	Hs.83765	dihydrofolate reductase
HMGN4	209786_at	0.481	BC001282	Hs.236774	high mobility group nucleosomal binding domain 4
SFRS1	211784_s_at	0.479	BC006181	Hs.68714	splicing factor, arginine/serine-rich 1 (splicing factor 2, alternate splicing factor)
CYP51A1	202314_at	0.479	NM_000786	Hs.417077	cytochrome P450, family 51, subfamily A, polypeptide 1
GBE1	203282_at	0.478	NM_000158	Hs.436062	glucan (1,4-alpha-), branching enzyme 1
HTRA2	203089_s_at	0.477	NM_013247	Hs.558479	HtrA serine peptidase 2
ZNF544	218735_s_at	0.477	AA349848	Hs.438994	zinc finger protein 544
SKP2	203625_x_at	0.477	BG105365	Hs.23348	S-phase kinase-associated protein 2 (p45)
FNBP1	212288_at	0.476	AB011126	Hs.189409	formin binding protein 1
DHFR	202532_s_at	0.475	BC000192	Hs.83765	dihydrofolate reductase
STK39	202786_at	0.475	NM_013233	Hs.276271	serine threonine kinase 39 (STE20/SPS1 homolog, yeast)
ST14	216905_s_at	0.474	U20428	Hs.504315	suppression of tumorigenicity 14 (colon carcinoma, matriptase, epithin)
KIAA0182	212057_at	0.472	AA206161	Hs.461647	KIAA0182 protein
RANBP6	213019_at	0.472	AI123233	Hs.167496	RAN binding protein 6
GPSM2	221922_at	0.472	AW195581	Hs.489353	G-protein signalling modulator 2 (AGS3-like, C. elegans)
OBSL1	212775_at	0.471	AI978623	Hs.526594	obscurin-like 1
ACOT7	208002_s_at	0.47	NM_007274	Hs.126137	acyl-CoA thioesterase 7
RAB4A	203582_s_at	0.47	NM_004578	Hs.296169	RAB4A, member RAS oncogene family
TXNDC13	201581_at	0.47	BF572868	Hs.169358	thioredoxin domain containing 13
ZFR	201857_at	0.47	NM_016107	Hs.435231	zinc finger RNA binding protein
E2IG5	220942_x_at	0.469	NM_014367	Hs.5243	growth and transformation-dependent protein
SFRS14	214092_x_at	0.468	AI928127	Hs.515271	splicing factor, arginine/serine-rich 14
HMGN1	200943_at	0.468	NM_004965	Hs.356285	high-mobility group nucleosome binding domain 1
ATP5L	208745_at	0.468	AA917672	Hs.558453	ATP synthase, H+ transporting, mitochondrial F0 complex, subunit g
TMSL8	205347_s_at	0.468	NM_021992	Hs.56145	thymosin-like 8
PYGL	202990_at	0.468	NM_002863	Hs.282417	phosphorylase, glycogen; liver (Hers disease, glycogen storage disease type VI)
PPM1G	200913_at	0.467	NM_002707	Hs.17883	protein phosphatase 1G (formerly 2C), magnesium-dependent, gamma isoform
CYB5-M	201634_s_at	0.466	NM_030579	Hs.461131	outer mitochondrial membrane cytochrome b5
RPS17	216348_at	0.466	AL049693	Hs.512525	ribosomal protein S17 ; similar to 40S ribosomal protein S17
RAD17	207405_s_at	0.466	NM_002873	Hs.16184	RAD17 homolog (S. pombe)
ELA2	206871_at	0.466	NM_001972	Hs.99863	elastase 2, neutrophil
FLJ22386	218394_at	0.465	NM_024589	Hs.459795	leucine zipper domain protein
RAB4A	203581_at	0.465	BC002438	Hs.296169	RAB4A, member RAS oncogene family
ZNF505	208119_s_at	0.464	NM_031218	Hs.301059	zinc finger protein 505 ; zinc finger protein 505
C14orf87	221932_s_at	0.463	AA133341	Hs.532683	chromosome 14 open reading frame 87
C22orf9	217118_s_at	0.463	AK025608	Hs.369682	chromosome 22 open reading frame 9
RBM8A	213852_at	0.462	BG289199	Hs.555911	RNA binding motif protein 8A
DKFZP564C152	216028_at	0.462	AL049980	Hs.184216	DKFZP564C152 protein

HSPA4L	205543_at	0.462	NM_014278	Hs.135554	heat shock 70kDa protein 4-like
GOLGA8A	208798_x_at	0.462	AF204231	Hs.182982	golgi autoantigen, golgin subfamily a, 8A
C14orf138	218940_at	0.462	NM_024558	Hs.558541	chromosome 14 open reading frame 138
RALBP1	202845_s_at	0.461	NM_006788	Hs.528993	ralA binding protein 1
6-Mar	201737_s_at	0.461	NM_005885	Hs.432862	membrane-associated ring finger (C3HC4) 6
DCPS	218774_at	0.46	NM_014026	Hs.504249	decapping enzyme, scavenger
RPL35	211976_at	0.46	AK026168	Hs.485155	Ribosomal protein L35
SLC35A3	206770_s_at	0.46	NM_012243	Hs.448979	solute carrier family 35 (UDP-N-acetylglucosamine (UDP-GlcNAc) transporter), member A3
TSTA3	36936_at	0.458	U58766	Hs.404119	tissue specific transplantation antigen P35B
C1orf41	203960_s_at	0.458	NM_016126	Hs.525462	chromosome 1 open reading frame 41
CENTG2	204066_s_at	0.458	NM_014914	Hs.435039	centaurin, gamma 2
LY75	205668_at	0.457	NM_002349	Hs.153563	lymphocyte antigen 75
RPL18A	216383_at	0.457	U52111	Hs.558383	ribosomal protein L18a ; similar to ribosomal protein L18a; 60S ribosomal protein L18a
JARID1B	201549_x_at	0.457	NM_006618	Hs.443650	Jumonji, AT rich interactive domain 1B (RBP2-like)
NXT2	209628_at	0.456	AK023289	Hs.25010	nuclear transport factor 2-like export factor 2
CKAP2	218252_at	0.456	NM_018204	Hs.444028	cytoskeleton associated protein 2
EDD1	208883_at	0.456	BF515424	Hs.492445	E3 ubiquitin protein ligase, HECT domain containing, 1
RAB27A	209515_s_at	0.456	U38654	Hs.298651	RAB27A, member RAS oncogene family
MXI1	202364_at	0.456	NM_005962	Hs.501023	MAX interactor 1 ; MAX interactor 1
UNG	202330_s_at	0.455	NM_003362	Hs.191334	uracil-DNA glycosylase
PRKACB	202742_s_at	0.455	NM_002731	Hs.487325	protein kinase, cAMP-dependent, catalytic, beta
BST1	205715_at	0.455	NM_004334	Hs.169998	bone marrow stromal cell antigen 1
GCSH	213129_s_at	0.455	AI970157	Hs.435741	glycine cleavage system protein H (aminomethyl carrier)
SFRS1	208863_s_at	0.454	M72709	Hs.68714	splicing factor, arginine/serine-rich 1 (splicing factor 2, alternate splicing factor)
PPIG	208993_s_at	0.454	AW340788	Hs.470544	peptidyl-prolyl isomerase G (cyclophilin G)
DCXR	217973_at	0.454	NM_016286	Hs.9857	dicarbonyl/L-xylulose reductase
PTBP2	218683_at	0.454	NM_021190	Hs.269895	polypyrimidine tract binding protein 2
SPFH1	202441_at	0.454	AL568449	Hs.150087	SPFH domain family, member 1
PIGB	205452_at	0.453	NM_004855	Hs.126115	phosphatidylinositol glycan, class B
CROP	220044_x_at	0.453	NM_016424	Hs.130293	cisplatin resistance-associated overexpressed protein
UAP1	209340_at	0.453	S73498	Hs.492859	UDP-N-acteylglucosamine pyrophosphorylase 1
WT1	206067_s_at	0.452	NM_024426	Hs.555896	Wilms tumor 1
CENPF	207828_s_at	0.452	NM_005196	Hs.497741	centromere protein F, 350/400ka (mitosin)
GPSM2	205240_at	0.452	NM_013296	Hs.489353	G-protein signalling modulator 2 (AGS3-like, C. elegans)
MYB	204798_at	0.451	NM_005375	Hs.531941	v-myb myeloblastosis viral oncogene homolog (avian)
CLEC11A	210783_x_at	0.451	D86586	Hs.512680	C-type lectin domain family 11, member A
C13orf10	218422_s_at	0.451	NM_022118	Hs.558528	chromosome 13 open reading frame 10
NPTX1	204684_at	0.45	NM_002522	Hs.514556	neuronal pentraxin I
USP7	201498_at	0.449	AI160440	Hs.386939	Ubiquitin specific peptidase 7 (herpes virus-associated)
PDCD4	202731_at	0.449	NM_014456	Hs.232543	programmed cell death 4 (neoplastic transformation inhibitor)
KTN1 ; PDIA6	214709_s_at	0.449	Z22551	Hs.509414	kinectin 1 (kinesin receptor) ; protein disulfide isomerase family A, member 6
COX11	211727_s_at	0.447	BC005895	Hs.96530	COX11 homolog, cytochrome c oxidase assembly protein (yeast)

ATP5O	216954_x_at	0.447	S77356	Hs.409140	ATP synthase, H+ transporting, mitochondrial F1 complex, O subunit (oligomycin sensitivity conferring protein)
TFB2M	218605_at	0.447	NM_022366	Hs.7395	transcription factor B2, mitochondrial
ZFR	201856_s_at	0.446	BC000376	Hs.435231	zinc finger RNA binding protein
EAF2	219551_at	0.443	NM_018456	Hs.477325	ELL associated factor 2
PRPF38B	218040_at	0.442	NM_018061	Hs.342307	PRP38 pre-mRNA processing factor 38 (yeast) domain containing B
CES1	209616_s_at	0.442	S73751	Hs.535486	carboxylesterase 1 (monocyte/macrophage serine esterase 1)
RNGTT	204208_at	0.441	NM_003800	Hs.127219	RNA guanylyltransferase and 5'-phosphatase
	212764_at	0.441	AI806174		
CCNA1	205899_at	0.441	NM_003914	Hs.417050	cyclin A1
AGTPBP1	204500_s_at	0.441	NM_015239	Hs.494321	ATP/GTP binding protein 1
PCGF4	202265_at	0.441	NM_005180	Hs.380403	polycomb group ring finger 4
ECH1	200789_at	0.44	NM_001398	Hs.196176	enoyl Coenzyme A hydratase 1, peroxisomal
SMC5L1	212927_at	0.44	AB011166	Hs.534189	SMC5 structural maintenance of chromosomes 5-like 1 (yeast)
PKP4	201928_at	0.438	AA194254	Hs.407580	plakophilin 4
FAH	202862_at	0.437	NM_000137	Hs.73875	fumarylacetoacetate hydrolase (fumarylacetoacetase)
HYAL3	211728_s_at	0.437	BC005896	Hs.129910	hyaluronoglucosaminidase 3 ; hyaluronoglucosaminidase 3
SFPQ	214016_s_at	0.437	AL558875	Hs.355934	Splicing factor proline/glutamine-rich (polypyrimidine tract binding protein associated)
RPS27L	218007_s_at	0.437	NM_015920	Hs.108957	ribosomal protein S27-like
UQCRB	209066_x_at	0.436	M26700	Hs.131255	ubiquinol-cytochrome c reductase binding protein
PNN	212036_s_at	0.435	AW152664	Hs.409965	pinin, desmosome associated protein
TIMP3	201147_s_at	0.434	BF347089	Hs.297324	TIMP metallopeptidase inhibitor 3 (Sorsby fundus dystrophy, pseudoinflammatory)
TARBP1	202813_at	0.434	NM_005646	Hs.498115	TAR (HIV) RNA binding protein 1
DIPA	204610_s_at	0.434	NM_006848	Hs.66713	hepatitis delta antigen-interacting protein A
CROP	203804_s_at	0.433	NM_006107	Hs.130293	cisplatin resistance-associated overexpressed protein
NXF3	220110_s_at	0.433	NM_022052	Hs.60386	nuclear RNA export factor 3
GGA2	214190_x_at	0.432	AI799984	Hs.460336	golgi associated, gamma adaptin ear containing, ARF binding protein 2
ZFP36L2	201367_s_at	0.432	AI356398	Hs.503093	zinc finger protein 36, C3H type-like 2
PARP8	219033_at	0.431	NM_024615	Hs.369581	poly (ADP-ribose) polymerase family, member 8
AKR7A2	214259_s_at	0.43	AI144075	Hs.512807	aldo-keto reductase family 7, member A2 (aflatoxin aldehyde reductase)
KIF23	204709_s_at	0.43	NM_004856	Hs.270845	kinesin family member 23
ANKRD15	213005_s_at	0.43	D79994	Hs.493272	ankyrin repeat domain 15
ZMYND11	202136_at	0.428	BE250417	Hs.292265	zinc finger, MYND domain containing 11
LILRA2	211101_x_at	0.428	U82276	Hs.534394	leukocyte immunoglobulin-like receptor, subfamily A (with TM domain), member 2
ACTN1	211160_x_at	0.427	M95178	Hs.509765	actinin, alpha 1
UCHL3	204616_at	0.427	NM_006002	Hs.162241	ubiquitin carboxyl-terminal esterase L3 (ubiquitin thiolesterase)
EIF4A2	200912_s_at	0.426	NM_001967	Hs.478553	eukaryotic translation initiation factor 4A, isoform 2
KIAA0746	212314_at	0.424	AB018289	Hs.479384	KIAA0746 protein
PMS1	213677_s_at	0.423	BG434893	Hs.111749	PMS1 postmeiotic segregation increased 1 (S. cerevisiae)
SLC7A6	203579_s_at	0.422	AI660619		solute carrier family 7 (cationic amino acid transporter, y+ system), member 6
NOL8	218244_at	0.422	NM_017948	Hs.442199	nucleolar protein 8
CLEC11A	205131_x_at	0.421	NM_002975	Hs.512680	C-type lectin domain family 11, member A
LAS1L	208117_s_at	0.42	NM_031206	Hs.522675	LAS1-like (S. cerevisiae) ; LAS1-like (S. cerevisiae)

AKR7A2	202139_at	0.42	NM_003689	Hs.512807	aldo-keto reductase family 7, member A2 (aflatoxin aldehyde reductase)
PCM1	202174_s_at	0.42	NM_006197	Hs.491148	pericentriolar material 1
LOC221981	214920_at	0.419	R33964		hypothetical protein LOC221981
EIF4B	211938_at	0.417	BF247371	Hs.292063	eukaryotic translation initiation factor 4B
ZNF91	206059_at	0.417	NM_003430	Hs.558418	zinc finger protein 91 (HPF7, HTF10)
TOP2B	211987_at	0.415	NM_001068	Hs.475733	topoisomerase (DNA) II beta 180kDa
ZFP36L2	201368_at	0.414	U07802	Hs.503093	zinc finger protein 36, C3H type-like 2
SLC39A4	219215_s_at	0.413	NM_017767	Hs.521934	solute carrier family 39 (zinc transporter), member 4
MYCBP	203359_s_at	0.412	AL525412	Hs.370040	c-myc binding protein
HTATSF1	202602_s_at	0.411	NM_014500	Hs.204475	HIV TAT specific factor 1
PRPF31	202407_s_at	0.411	BF342707	Hs.515598	PRP31 pre-mRNA processing factor 31 homolog (yeast)
NNT	202783_at	0.411	U40490	Hs.482043	nicotinamide nucleotide transhydrogenase
CBFA2T3	208056_s_at	0.41	NM_005187	Hs.513811	core-binding factor, runt domain, alpha subunit 2; translocated to, 3
BXDC2	219177_at	0.409	NM_018321	Hs.38114	brix domain containing 2
HOMER3	215489_x_at	0.408	AI871287	Hs.410683	homer homolog 3 (Drosophila)
TAF9	202168_at	0.408	NM_003187	Hs.248941	TAF9 RNA polymerase II, TATA box binding protein (TBP)-associated factor, 32kDa
SERTAD2	202657_s_at	0.406	NM_014755	Hs.77293	SERTA domain containing 2
TRGC2	211144_x_at	0.405	M30894		T cell receptor gamma constant 2
SMYD3	218788_s_at	0.404	NM_022743	Hs.127406	SET and MYND domain containing 3
RCN2	201486_at	0.404	NM_002902	Hs.79088	reticulocalbin 2, EF-hand calcium binding domain
PDLIM2	219165_at	0.404	NM_021630	Hs.555972	PDZ and LIM domain 2 (mystique)
PKP4	201929_s_at	0.404	NM_003628	Hs.407580	plakophilin 4
TTC3	208662_s_at	0.402	AI885338	Hs.368214	tetratricopeptide repeat domain 3
FLJ10287	219130_at	0.398	NM_019083	Hs.440371	hypothetical protein FLJ10287
C2orf17	222129_at	0.398	AK026155	Hs.516707	Chromosome 2 open reading frame 17
MASK	218499_at	0.398	NM_016542	Hs.444247	Mst3 and SOK1-related kinase
C6orf111	212179_at	0.396	AW157501	Hs.520287	chromosome 6 open reading frame 111
AFF1	201924_at	0.395	NM_005935	Hs.480190	AF4/FMR2 family, member 1
CGI-116	218628_at	0.394	NM_016053	Hs.405692	CGI-116 protein
PTPRF	200637_s_at	0.394	AI762627	Hs.272062	protein tyrosine phosphatase, receptor type, F
ABCD3	202850_at	0.394	NM_002858	Hs.76781	ATP-binding cassette, sub-family D (ALD), member 3
ALCAM	201951_at	0.393	BF242905	Hs.150693	activated leukocyte cell adhesion molecule
TCFL5	204849_at	0.392	NM_006602	Hs.30696	transcription factor-like 5 (basic helix-loop-helix)
PRTN3	207341_at	0.392	NM_002777	Hs.928	proteinase 3 (serine proteinase, neutrophil, Wegener granulomatosis autoantigen)
E2F8	219990_at	0.391	NM_024680	Hs.523526	E2F transcription factor 8
	212498_at	0.391	AF056433	Hs.561432	Full-length cDNA clone CS0DM001YA04 of Fetal liver of Homo sapiens (human)
SLC35A1	203306_s_at	0.391	NM_006416	Hs.423163	solute carrier family 35 (CMP-sialic acid transporter), member A1
RGC32	218723_s_at	0.391	NM_014059	Hs.507866	response gene to complement 32
MICAL1	218376_s_at	0.389	NM_022765	Hs.33476	microtubule associated monoxygenase, calponin and LIM domain containing 1
STMN1	200783_s_at	0.389	NM_005563	Hs.209983	stathmin 1/oncoprotein 18
HNRPA3	211929_at	0.388	AA527502	Hs.516539	heterogeneous nuclear ribonucleoprotein A3
FUBP1	212847_at	0.382	AL036840	Hs.567255	Far upstream element (FUSE) binding protein 1

CRIP1	205081_at	0.382	NM_001311	Hs.70327	cysteine-rich protein 1 (intestinal)
HOMER3	204647_at	0.379	NM_004838	Hs.410683	homer homolog 3 (Drosophila)
MRPL33	203781_at	0.377	NM_004891	Hs.515879	mitochondrial ribosomal protein L33
RET	215771_x_at	0.376	X15786	Hs.350321	ret proto-oncogene (multiple endocrine neoplasia and medullary thyroid carcinoma 1, Hirschsprung disease)
TIMP3	201150_s_at	0.376	NM_000362	Hs.297324	TIMP metallopeptidase inhibitor 3 (Sorsby fundus dystrophy, pseudoinflammatory)
CD302	203799_at	0.374	NM_014880	Hs.130014	CD302 antigen
DKFZp434N2030	213701_at	0.373	AW299245	Hs.494204	hypothetical protein DKFZp434N2030
HNRPA0	201054_at	0.372	BE966599	Hs.96996	heterogeneous nuclear ribonucleoprotein A0
SACS	213262_at	0.371	AI932370	Hs.159492	spastic ataxia of Charlevoix-Saguenay (sacsin)
PLAC8	219014_at	0.371	NM_016619	Hs.546392	placenta-specific 8
CETN2	209194_at	0.369	BC005334	Hs.82794	centrin, EF-hand protein, 2
ZFP36L2	201369_s_at	0.369	NM_006887	Hs.503093	zinc finger protein 36, C3H type-like 2
PGRMC2	213227_at	0.368	BE879873	Hs.507910	progesterone receptor membrane component 2
NKTR	215338_s_at	0.368	AI688640	Hs.529509	natural killer-tumor recognition sequence
NDUFA5	201304_at	0.367	NM_005000	Hs.83916	NADH dehydrogenase (ubiquinone) 1 alpha subcomplex, 5, 13kDa
TMEM38B	218772_x_at	0.366	NM_018112	Hs.411925	transmembrane protein 38B
SPHAR	206272_at	0.365	NM_006542	Hs.511754	S-phase response (cyclin-related)
SAS10	209486_at	0.364	BC004546	Hs.322901	disrupter of silencing 10
IMPA2	203126_at	0.364	NM_014214	Hs.367992	inositol(myo)-1(or 4)-monophosphatase 2
C5	205500_at	0.36	NM_001735	Hs.494997	complement component 5
CD37	204192_at	0.358	NM_001774	Hs.166556	CD37 antigen
GATM	203178_at	0.357	NM_001482	Hs.75335	glycine amidinotransferase (L-arginine:glycine amidinotransferase)
IRF8	204057_at	0.355	AI073984	Hs.137427	interferon regulatory factor 8 ; interferon regulatory factor 8
PKP4	201927_s_at	0.354	BG292559	Hs.407580	plakophilin 4
EIF2C2	222294_s_at	0.353	AW971415	Hs.449415	Eukaryotic translation initiation factor 2C, 2
TRGC2	215806_x_at	0.35	M13231	Hs.534032	T cell receptor gamma constant 2 ; T cell receptor gamma variable 9
SGK3	220038_at	0.345	NM_013257	Hs.380877	serum/glucocorticoid regulated kinase family, member 3
P2RY2	206277_at	0.344	NM_002564	Hs.339	purinergic receptor P2Y, G-protein coupled, 2
	211600_at	0.344	U20489		
H2AFV	212205_at	0.338	AA534860	Hs.488189	H2A histone family, member V
BRP44	202427_s_at	0.337	NM_015415	Hs.517768	brain protein 44
BCL2	203685_at	0.336	NM_000633	Hs.150749	B-cell CLL/lymphoma 2
C22orf9	212421_at	0.336	AB023147	Hs.369682	chromosome 22 open reading frame 9
FLJ20054	219696_at	0.335	NM_019049	Hs.518926	hypothetical protein FLJ20054
KBTBD11	204301_at	0.334	NM_014867	Hs.5333	kelch repeat and BTB (POZ) domain containing 11
RBL2	212331_at	0.333	X76061	Hs.513609	retinoblastoma-like 2 (p130)
ALCAM	201952_at	0.331	AA156721	Hs.150693	activated leukocyte cell adhesion molecule
C13orf18	44790_s_at	0.328	AI129310	Hs.98117	chromosome 13 open reading frame 18
BEX1	218332_at	0.327	NM_018476	Hs.334370	brain expressed, X-linked 1
ARL1	201657_at	0.326	BE890745	Hs.372616	ADP-ribosylation factor-like 1
TRGC2	209813_x_at	0.315	M16768	Hs.534032	T cell receptor gamma constant 2 ; T cell receptor gamma constant 2 ; T cell receptor gamma variable 9
LOC283768	213737_x_at	0.315	AI620911	Hs.146211	hypothetical LOC283768 ; similar to hypothetical protein ; similar to hypothetical protein

TUBA1	212242_at	0.313	AL565074	Hs.75318	tubulin, alpha 1 (testis specific)
HT008	218099_at	0.309	NM_018469	Hs.175414	uncharacterized hypothalamus protein HT008
CAT	201432_at	0.308	NM_001752	Hs.502302	catalase
TTC3	210645_s_at	0.3	D83077	Hs.368214	tetratricopeptide repeat domain 3
C20orf103	219463_at	0.3	NM_012261	Hs.22920	chromosome 20 open reading frame 103
DHRS9	219799_s_at	0.296	NM_005771	Hs.179608	dehydrogenase/reductase (SDR family) member 9
RNASET2	217984_at	0.295	NM_003730	Hs.529989	ribonuclease T2
LILRA2	211102_s_at	0.293	U82277	Hs.534394	leukocyte immunoglobulin-like receptor, subfamily A (with TM domain), member 2
ATP1B1	201242_s_at	0.289	BC000006	Hs.291196	ATPase, Na+/K+ transporting, beta 1 polypeptide
PTPRF	200636_s_at	0.282	NM_002840	Hs.272062	protein tyrosine phosphatase, receptor type, F
LILRA2	207857_at	0.264	NM_006866	Hs.534394	leukocyte immunoglobulin-like receptor, subfamily A (with TM domain), member 2
MEST	202016_at	0.25	NM_002402	Hs.270978	mesoderm specific transcript homolog (mouse)
WRB	202749_at	0.248	NM_004627	Hs.198308	tryptophan rich basic protein
RASGRP2	214369_s_at	0.243	AI688812	Hs.99491	RAS guanyl releasing protein 2 (calcium and DAG-regulated)
CAT	211922_s_at	0.24	AY028632	Hs.502302	catalase ; catalase
GM632	55872_at	0.235	AI493119	Hs.551552	KIAA1196 protein
PDCD4	212593_s_at	0.234	N92498	Hs.232543	programmed cell death 4 (neoplastic transformation inhibitor)
NUCB2	203675_at	0.233	NM_005013	Hs.128686	nucleobindin 2
RET	211421_s_at	0.228	M31213	Hs.350321	Ret proto-oncogene (multiple endocrine neoplasia and medullary thyroid carcinoma 1, Hirschsprung disease)
CTSG	205653_at	0.216	NM_001911	Hs.421724	cathepsin G
MS4A6A	219666_at	0.201	NM_022349	Hs.523702	membrane-spanning 4-domains, subfamily A, member 6A
C11orf32	212560_at	0.15	AV728268		chromosome 11 open reading frame 32
