# Supplementary material for: Ganoderma lucidum Polysaccharides Induce Macrophage-Like Differentiation in Human Leukemia THP-1 Cells via Caspase and p53 Activation
Source: Evid Based Complement Alternat Med. 2011 Jan 4;2011:358717. doi: 10.1093/ecam/nep107 (PMC3135330; doi:10.1093/ecam/nep107)
Supplement: Supplementary file 3 [file 358717.f3.pdf]

supplementary Table 3. Significantly genetic networks affected by F3.

ID	Molecules in networks by IPA tool analysis	Score	Focus Molecules	Top functions
	**F3 treatment for 6 hrs**			
1	**CXCL6, DUSP5, G0S2, GBP1, IFI27, IFI35, IFIT3, IFITM1,** IL-1R**, IL10RA, IL12B, IL1B, IL23A,** IRAK**, IRF1, IRF2, IRF7, IRF8, ISG15, ISGF3G, KCNAB2, MYD88, OAS1, OAS2, PLSCR1, PSMB9, PYCARD, RSAD2, RTN1, SLC11A2, SQSTM1, STAT1, STAT2, STAT4, UGCG**	50	33	Immune Response<br>Cell Signaling
2	**BCL2, BCL2A1,** Calcineurin protein(s)**, CASP1, CASP4, CASP5, CASP7,** Caspase**,** Caspase 3/7**, CAT, CCL7, CCR1, CKB, CLIC4, DUSP1, EIF4EBP1, FGR, HIP1, IFI6, IFIH1, IFNGR2, MCL1, MT1E, MTF1, PIM2, PLAUR, PMAIP1, PML, SP100, SPHK1, SRD5A1, STAT3, STK3, TNFAIP2, TNFSF10**	47	32	Cell Death
3	**BTG3, CASP10, CHST2, ECGF1, EHD1, GBP2 (includes EG:2634), HIVEP1, IFIT5,** Iκb**, IKBKE,** IKK**,** IL1/IL6/TNF**, KYNU, LAMP3, LITAF, LSS,** NF-κB**, NINJ1, OAS3, OASL, OPTN,** peptidase**, PLA2G7, RAB8A, RIPK1, SERPINB1, TNF,** Tnf receptor**, TNFAIP3, TNFAIP8, TNFRSF21, TNIP1, TRADD, TRAF1, WTAP**	40	29	Cell Death
4	Ahr-aryl hydrocarbon-Arnt**, ATF3, BCL6, BIRC3, BTG1, C3AR1, CBFA2T3, CCL8, CYP1B1, DACH1, EHD4,** ERK1/2**,** Fos-Jun**, FTH1, GCLM, HESX1, HHEX, HMOX1,** JINK1/2**, JUN,** JUN/JUNB/JUND**, JUNB, MAFF, MT2A, MXI1,** N-cor**, NFE2L2, NFE2L3, NQO1, PDCD4, PLAGL1, PTX3,** Rar**, RUNX1T1, SLC19A1**	38	28	Gene Expression<br>Cellular Development<br>Cell Death
5	Akt**, BCL3,** C8**, CACYBP,** Caspase 8/10**, CCR7, CD59, CD70, CFLAR, CXCL2, CXCL3, CXCL9, CXCL13, DDX3X, FAS, HPSE,** Hsp27**, IER3, IL8, LTB, NFKB1, NFKB2, NFKBIE (includes EG:4794), OLIG2, PDE4B,** Pdgf Ab**, PEA15, PHLDA1, REL, RUNX3, TCR, TNFR/Fas, TNFRSF9, TNFRSF1B, UXS1**	38	28	Hematological System Development and Function<br>Immune System Development and Function
	**F3 treatment for 24 hrs**			
1	**ALCAM, AMPD3, BST2, CHST2, ECGF1, ECH1, EHD1, EXT1, GBP2 (includes EG:2634),**	45	31	Cell Death

	**HNRPA0, IFIH1, IFIT5,** IL-1R, IL1/IL6/TNF, **IL10RA,** IRAK, **IRF7, IRF8, ISG15, KYNU, LAMP3, MAFF, MYD88, NINJ1, OAS3, OASL, OPTN,** peptidase, **PLA2G7, PLSCR1, PMAIP1, SHFM1, TLR7, TNF, UBE1L**			Hematological System Development and Function<br>Cellular Development
2	**ABCD3,** CD8, **CD163, CTSS, G0S2, GBP1, HLA-A, HLA-B, HLA-C, HLA-E, HLA-F, HLA-G, IL1B, IRF1, IRF2, ISGF3G, LILRB1, LILRB2,** MHC Class I, MHC I-α, **OAS1, OAS2, PROCR, PRTN3, PSMB9, RSAD2, RTN1, SLC11A2,** Tap, **TAP1, TAP2, TAPBP, TNFSF10, UAP1, UGCG**	45	31	Cell Signaling<br>Immune Response<br>Cellular Growth and Proliferation
3	Akt, **CASP10, CAT, CD40, CD83, CFLAR, CXCL13, DKC1, FAS, GRB10,** Hsp27, **IER3, IKBKE, IL8, IL7R, MUC1,** NF-κB, **NFKB1, NFKB2, NFKBIA, NFKBIE (includes EG:4794), OLIG2, PROS1, PTAFR, PTPRF, RELB, RET, SQSTM1, TANK,** Tnf receptor, **TNFAIP3, TNFRSF9, TNFRSF1B, TNIP1, TRAF1**	45	31	Cell Death
4	**ARL4C, CD37, CD53,** Cyclooxygenase, **DDX58, EBI3, ENPP2, GK, HOMER3, IFI27, IFI35, IFIT1, IFIT2, IFITM1, IL6, IL23A, INDO,** IRS, **ISG20,** JAK, **PDLIM2,** PPARα-RXRα, **PPP1R15A (includes EG:23645), PSCDBP, SEMA4A, SLC7A7,** SOCS, **SP110,** STAT, **STAT1, STAT2, STAT4,** Timp, **TTK, WARS**	38	28	Immune Response
5	**AHR,** Ahr-aryl hydrocarbon-Arnt, **BMI1,** C1q, **CCNA1, CDKN1A, CENPF,** Cyclin A, Cyclin E, **CYP1B1, DHFR, DSE,** E2f, **E2F8, EID1, IFI16, LGALS3BP, LY96, MYB, PIM1, PNN, PPM1G, PSAP, PTX3,** Rb, **RBL2,** Scf, **SKP2, SOD2,** Sphk, **STMN1, TCF7L2, TFAP2A, TNFAIP6, TOP2A**	36	27	Cell Cycle<br>Cellular Growth and Proliferation

* Bold genes are those determined by microarray analysis and identified by IPA tool. A score of > 3 is considered significant ($p < 0.001$)
